# Bromide-assisted chemoselective Heck reaction of 3-bromoindazoles under high-speed ball-milling conditions: synthesis of axitinib

**DOI:** 10.3762/bjoc.14.66

**Published:** 2018-04-06

**Authors:** Jingbo Yu, Zikun Hong, Xinjie Yang, Yu Jiang, Zhijiang Jiang, Weike Su

**Affiliations:** 1 Collaborative Innovation Center of Yangtze River Delta Region Green Pharmaceuticals, Zhejiang University of Technology, Hangzhou 310014, PR China; 2 College of Pharmaceutical Sciences, Zhejiang University of Technology, Hangzhou 310014, PR China

**Keywords:** axitinib, ball-milling, dehalogenation, Heck reaction, indazoles

## Abstract

A mechanically-activated chemoselective Heck coupling for the synthesis of 3-vinylindazoles has been developed with the aid of catalytic amounts of TBAB and NaBr as both dehalogenation restrainer and grinding auxiliary. After tuning of the chemical conditions and mechanical parameters, a series of non-activated 3-bromoindazoles and a broad scope of olefins worked well to give the corresponding coupling products in good to excellent yields. A further application of this protocol was performed in a two-step mechanochemical Heck/Migita cross coupling, which provided a highly efficient route for the synthesis of axitinib.

## Introduction

The palladium-catalyzed vinylation of alkenes in the presence of a base, known as the Heck reaction (Mizoroki–Heck reaction), is one of the most important transition-metal-catalyzed reactions [1–2], which has shown itself as a powerful synthetic tool in both academic and industrial practice [3–7]. The transformation has been enrolled as key steps for numerous synthetic routes, including the recent President Green Chemistry Award winner route of letermovir [8].

**Scheme 1:**
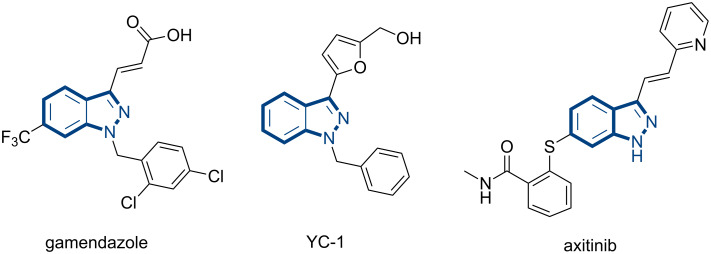
Representative pharmaceutically useful indazoles.

Hitherto, highly effective systems had been developed for the aryliodines that participated in Heck reactions with turn-over numbers of >1000 [9–10]. However, the couplings of bromo and chloro derivatives with unactivated alkenes still remain challenging. Though aryl bromides are always interesting substrates for industrial applications [11–12], possessing the characteristics of lower cost, easier to obtain and stable to store, they face the problem of dehalogenations especially under metal-catalyzed reactions [8,13–18], affecting the reaction yield and selectivity. Currently, the Heck reaction is usually carried out by adding an excess of phase-transfer catalyst such as tetrabutylammonium bromide (TBAB) or tetrabutylammonium iodide (TBAI) to increase the reaction yield under both solvent-heating [19–23] and solvent-free conditions [24–27]. Despite two proposals for the role of quaternary ammonium salts NR_4_^+^X^−^, (1) Pd(0) stablizer and (2) phase transfer were suggested [28–30], their effects and functions remain unrevealed. Hence, we want to get insight to the reaction pathway and the actual functions of NR_4_^+^X^−^ for better inhibiting the dehalogenation of aryl bromides, wherever possible. Herein, 3-bromoindazoles were chosen as model substrates not only for their low activity and easy dehalogenation properties, but also for their potential applications in the synthesis of natural products and pharmaceuticals, such as gamendazole [31–32], YC-1 [33–34] and axitinib [35–38] (Scheme 1).

**Scheme 2:**
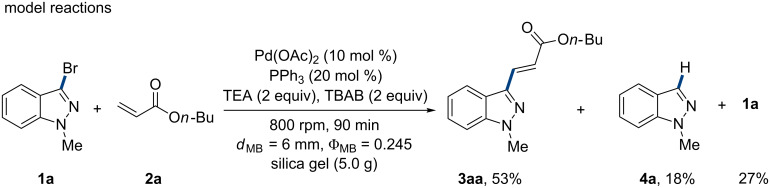
Model Heck reaction of 3-bromo-*N*-methyl-1*H*-indazole (**1a**) and *n*-butyl acrylate (**2a**). (173 stainless-steel balls (*d*_MB_ = 6 mm, Ф_MB_ = 0.245) were used. *d*_MB_ = milling ball diameter. Ф_MB_ = milling ball filling degree.)

Mechanochemistry as a burgeoning technique to promote solvent-free reactions has led to remarkable advances [39–42], particularly for cross-coupling reactions [43–45], involving Heck coupling with the aid of stoichiometric amounts of TBAB [24–27]. However, for inert and liable to dehalogenation bromo-heteroarenes, no desired response had been obtained yet. Thus, this work was going to establish a mild and chemoselective olefination of 3-bromoindazoles under ball-milling conditions (Scheme 2).

## Results and Discussion

### Optimisation of chemical conditons

At the commencement of the investigation, the cross-coupling between 3-bromo-1-methyl-1*H*-indazole (**1a**) and *n*-butyl acrylate (**2a**) was chosen as model reaction (Scheme 2). The initial attempts using our previous established conditions [24–25], gave only moderate conversion with a low yield of 53%. Noticeably, considerable amounts of 1-methyl-1*H*-indazole (**4a**) were obtained, which implicated the existence of a significant debromination process. As expected, the situation was more badly when the reaction was conducted under classic solvent-heating conditions (see Scheme S1 in Supporting Information File 1). Subsequently, the optimization was carried out to improve the performance of the reaction. Palladium catalysts and ligands were firstly screened, which showed Pd(OAc)_2_/PPh_3_ as the most efficient catalyst system (Table 1, entry 5). The catalyst loading could be reduced to 5 mol % without depriving of the product yield (Table 1, entry 23). Other Pd catalysts and phosphorous ligands displayed little effects on the reaction selectivity and product yield (Table 1, entries 1–4 and entries 6–9). As expected, without any catalyst and ligand, the reaction cannot proceed (Table 1, entry 10). For the investigated bases, triethylamine (TEA) exhibited the best result though it was reported to donate hydrides to arylpalladium species and lead to dehalogenation [46]. While different from literature report [47], no improvement was found in the reaction selectivity when replacing TEA by 1,4-diazabicyclo[2.2.2]octane (DABCO) (Table 1, entry 11). Besides, other organic or inorganic bases gave poor results (Table 1, entries 12–18).

**Table 1:** Optimisation of the reaction conditions for the olefination of 3-bromoindazoles.^a^

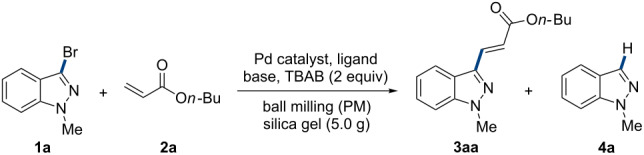				

Entry	Catalyst (mol %)	Ligand (mol %)	Base (equiv)	Yield (%) **3aa**/**4a**

1	Pd(OAc)_2_ (10)	Xantphos (20)	TEA (2)	43/24
2	Pd(OAc)_2_ (10)	dppf (20)	TEA (2)	44/21
3	Pd(OAc)_2_ (10)	dppe (20)	TEA (2)	45/19
4	Pd(OAc)_2_ (10)	P(*o*-tol)_3_ (20)	TEA (2)	32/22
5	Pd(OAc)_2_ (10)	PPh_3_ (20)	TEA (2)	53/18
6	PdCl_2_ (10)	PPh_3_ (20)	TEA (2)	47/18
7	PdCl_2_(dppf) (10)	—	TEA (2)	47/20
8	Pd_2_(dba)_3_ (10)	—	TEA (2)	37/17
9	Pd(PPh_3_)_4_ (10)	—	TEA (2)	48/18
10	—	—	TEA (2)	0/30
11	Pd(OAc)_2_ (10)	PPh_3_ (20)	DABCO (2)	51/18
12	Pd(OAc)_2_ (10)	PPh_3_ (20)	DIPEA (2)	35/23
13	Pd(OAc)_2_ (10)	PPh_3_ (20)	DCHA (2)	35/18
14	Pd(OAc)_2_ (10)	PPh_3_ (20)	DBU (2)	16/16
15	Pd(OAc)_2_ (10)	PPh_3_ (20)	*t*-BuOK (2)	40/21
16	Pd(OAc)_2_ (10)	PPh_3_ (20)	K_2_CO_3_ (2)	30/19
17	Pd(OAc)_2_ (10)	PPh_3_ (20)	NaOH (2)	30/24
18	Pd(OAc)_2_ (10)	PPh_3_ (20)	NaHCO_3_ (2)	21/16
19	Pd(OAc)_2_ (10)	PPh_3_ (20)	TEA (1.5)	53/19
20	Pd(OAc)_2_ (10)	PPh_3_ (20)	TEA (1.2)	53/19
21	Pd(OAc)_2_ (10)	PPh_3_ (20)	TEA (1.0)	45/23
22	Pd(OAc)_2_ (20)	PPh_3_ (40)	TEA (1.2)	54/20
**23**	**Pd(OAc)_2 _(5)**	**PPh_3 _(10)**	**TEA (1.2)**	**53/19**
24	Pd(OAc)_2_ (2.5)	PPh_3_ (5)	TEA (1.2)	36/24

^a^Raction conditions: **1a** (1.5 mmol), **2a** (2.25 mmol), Pd catalyst, ligand, base, TBAB (3 mmol), and silica gel (5.0 g) were placed in an 80 mL stainless steel vessel along with 173 stainless-steel balls (*d*_MB_ = 6 mm, Ф_MB_ = 0.245), milling at 800 rpm for 90 min. TEA = triethylamine. DABCO = 1,4-diazabicyclo[2.2.2]octane. DIPEA = *N*,*N*-diisopropylethylamine. DCHA = dicyclohexylamine. DBU = 1,8-diazabicyclo[5.4.0]undec-7-ene.

**Figure 1:**
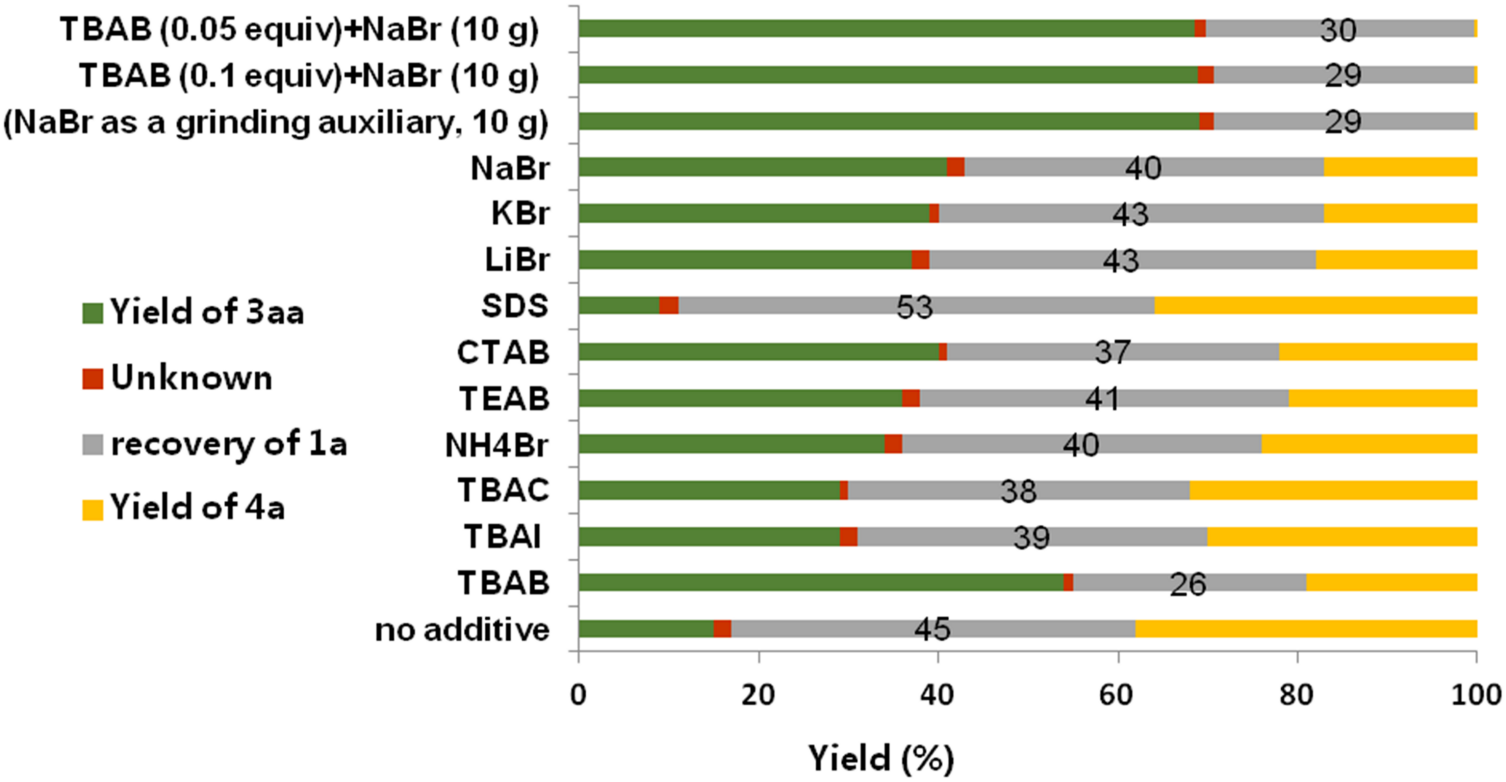
Investigation of additives in the Heck reaction: **1a** (1.5 mmol), **2a** (2.25 mmol), Pd(OAc)_2_ (5 mol %), PPh_3_ (10 mol %), TEA (1.8 mmol), additive (3.0 mmol), and silica gel (5.0 g) were placed in an 80 mL stainless steel vessel along with 173 stainless-steel balls (*d*_MB_ = 6 mm, Ф_MB_ = 0.245), milling at 800 rpm for 90 min.

Next, the additives were investigated in the coupling reactions (Figure 1, see detailed results in Supporting Information File 1, Table S1). Consistent to previous reports [3,24–27,48–52], TBAB helped to improve the product yield in this reaction, since without additive under the standard conditions, only 14% of target product **3aa** was obtained along with 38% of the dehalogenation counterpart **4a** (Table S1, entry 2). However, TBAI or tetrabutylammonium chloride (TBAC) did not provide a satisfactory result (Table S1, entries 3 and 4). It was interesting to find that using bromide salts (LiBr, NaBr, KBr and TBAB) as additives, not only the conversion rate of **1a** was increased, but also the side-product **4a** was suppressed, suggesting the bromide ion plays an important role in ameliorating the reaction selectivity (Table S1, entries 9–11). Further investigating the alkyl chain (R) of NR_4_^+^Br^−^ showed that the medium-length butyl was most efficient (Table S1, entries 5–7). In order to see whether the quaternary ammonium salt had the function of phase-transfer, other kinds of phase-transfer catalysts (SDS = sodium dodecyl sulfate) were also examined, however, this gave rise to a negative effect (Table S1, entry 8). To our delight, when we replaced the grinding auxiliary by sodium bromide, a moderate product yield (69%) and an excellent selectivity (trace of **4a**) were achieved (Table S1, entry 12). In this way, the amount of TBAB could even be reduced to 5 mol % (Table S1, entry 14).

### Influence of grinding auxiliary

In the process of ball milling, the grinding auxiliary was always found to be an efficient transfer medium between energy and reactant [53–55]. Thus, the effect of the grinding auxiliaries was also investigated. The results were shown in Table 2. Compared to using silica gel as grinding auxiliary, the reaction under neat conditions gave only 10% yield of **3aa**, but the dehalogenation was depressed (Table 2, entry 1), which showed silica gel played an important role of promoting the dehalogenations. When using a halogen salt (NaBr, KBr, NaCl) as grinding auxiliary, NaBr displayed the most efficiency (Table 2, entries 3–6), and lowering the amount of NaBr did not significantly influence the product yield (Table 2, entry 4). Other solid auxiliaries such as γ-Al_2_O_3_ and sand afforded a low yield of **3aa** with poor selectivity (Table 2, entries 7 and 8).

**Table 2:** Examination of the influence of the grinding auxiliary on the reaction outcome^a^.

Entry	Grinding auxiliary	Weight (g)	Yield (%) **3aa**/**4a**

1	—	—	10/trace
2	silica gel	5.0	53/19
3	NaBr	10.0	69/trace
4	NaBr	5.0	65/trace
5	KBr	5.0	63/trace
6	NaCl	5.0	27/5
7	γ-Al_2_O_3_ (neutral)	5.0	20/6
8	sand	5.0	30/10

^a^Influence of the grinding auxiliary on the Heck reaction: **1a** (1.5 mmol), **2a** (2.25 mmol), Pd(OAc)_2_ (5 mol %), PPh_3_ (10 mol %), TEA (1.8 mmol), TBAB (5 mol %), and grinding auxiliary were placed in an 80 mL stainless steel vessel along with 173 stainless-steel balls (*d*_MB_ = 6 mm, Ф_MB_ = 0.245), milling at 800 rpm for 90 min.

### Reaction pathway investigation

**Scheme 3:**
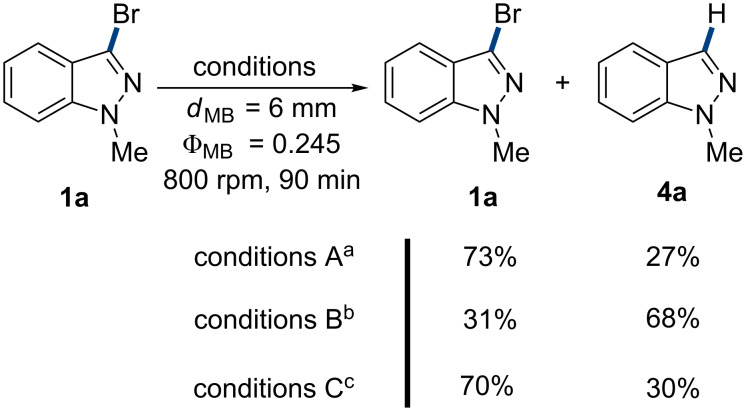
The control experiments. ^a^TEA (1.8 mmol), silica gel (5.0 g), ^b^Pd(OAc)_2_ (5 mol %), PPh_3_ (10 mol %), TEA (1.8 mmol), silica gel (5.0 g), ^c^Pd(OAc)_2_ (5 mol %), PPh_3_ (10 mol %), TEA (1.8 mmol), NaBr (10.0 g).

The control experiments were further conducted to disclose the reaction pathway (Scheme 3). In the case of taking TEA as sole reagent in the reaction, the substrate **1a** was converted to **4a** in 27% yield (conditions a), while the dehalogenation was exacerbated in the presence of Pd(OAc)_2_/PPh_3_ (conditions b). This phenomenon was in agreement with previous reports [14–16], that Pd-catalyzed homocoupling of aryl halides under alkaline heating conditions was often accompanied by dehalogenation as side reaction. When using NaBr as a grinding auxiliary instead of silica gel, the dehalogenation of **1a** was greatly inhibited (conditions c), but still gave **4a** in 30% without the presence of olefins.

**Scheme 4:**
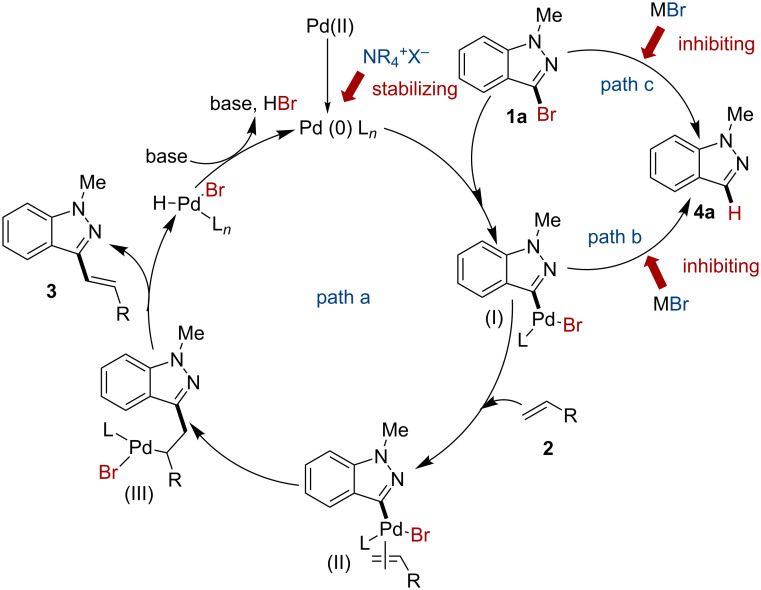
Plausible reaction pathway.

Based on the above research, the reaction pathway for this Heck reaction under mechanical ball milling conditions was proposed. As shown in path a, (Scheme 4). The reaction proceeded through Heck cross coupling, where a catalytic amount of TBAB was enough to stabilize the Pd(0). However, the oxidative addition intermediate **I** was unstable so that it was prone to produce **4a**, as depicted in path b*,* particularly in the absence of the coupling counterpart **2**. Besides, alkaline and high energy input conditions promoted the dehalogenation as shown in path c, while the addition of bromine salts helped diminishing this effect dramatically [56].

### Adjusting of mechanical parameters

**Figure 2:**
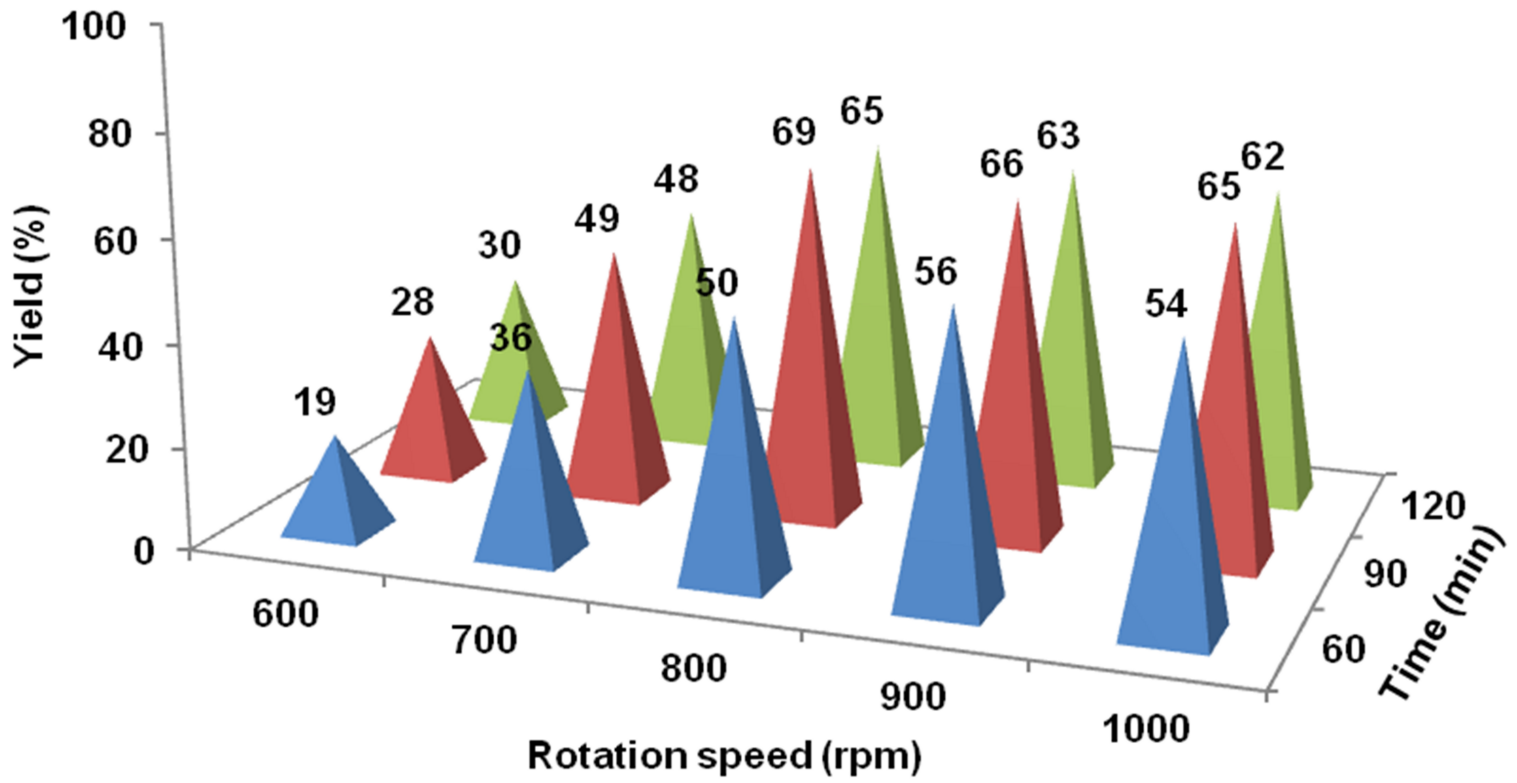
Influence of milling time and rotation speed on the Heck reaction: **1a** (1.5 mmol), **2a** (2.25 mmol), Pd(OAc)_2_ (5 mol %), PPh_3_ (10 mol %), TEA (1.8 mmol), TBAB (5 mol %), and NaBr (10.0 g) were placed in 80 mL stainless steel vessel with stainless-steel balls (*d*_MB_ = 6 mm, Ф_MB_ = 0.245).

Having identified the optimal chemical conditions for the reaction selectivity on the basis of the reaction pathway, we then focussed on the milling parameters such as rotation speed (*ν*_rot_), ball milling time (*t*), milling ball filling degree (Ф_MB_), and milling ball diameter (*d*_MB_), which usually play important roles in mechanochemistry processes [57–60]. First, the combined effect between ball-milling time and rotation speed was screened systematically (Figure 2). The results show a sharp increase of the product yield when elevating the rotation speed from 600 to 800 rpm, and a progressive increase of the yield by prolonging the reaction time from 60 to 90 min. Further increasing the rotation speed or prolonging the reaction time did not help to improve the product yield but promoted the occurrence of **4a** (see Table S2 in Supporting Information File 1), which mainly due to the redundant energy input spurred the dehalogenation.

**Figure 3:**
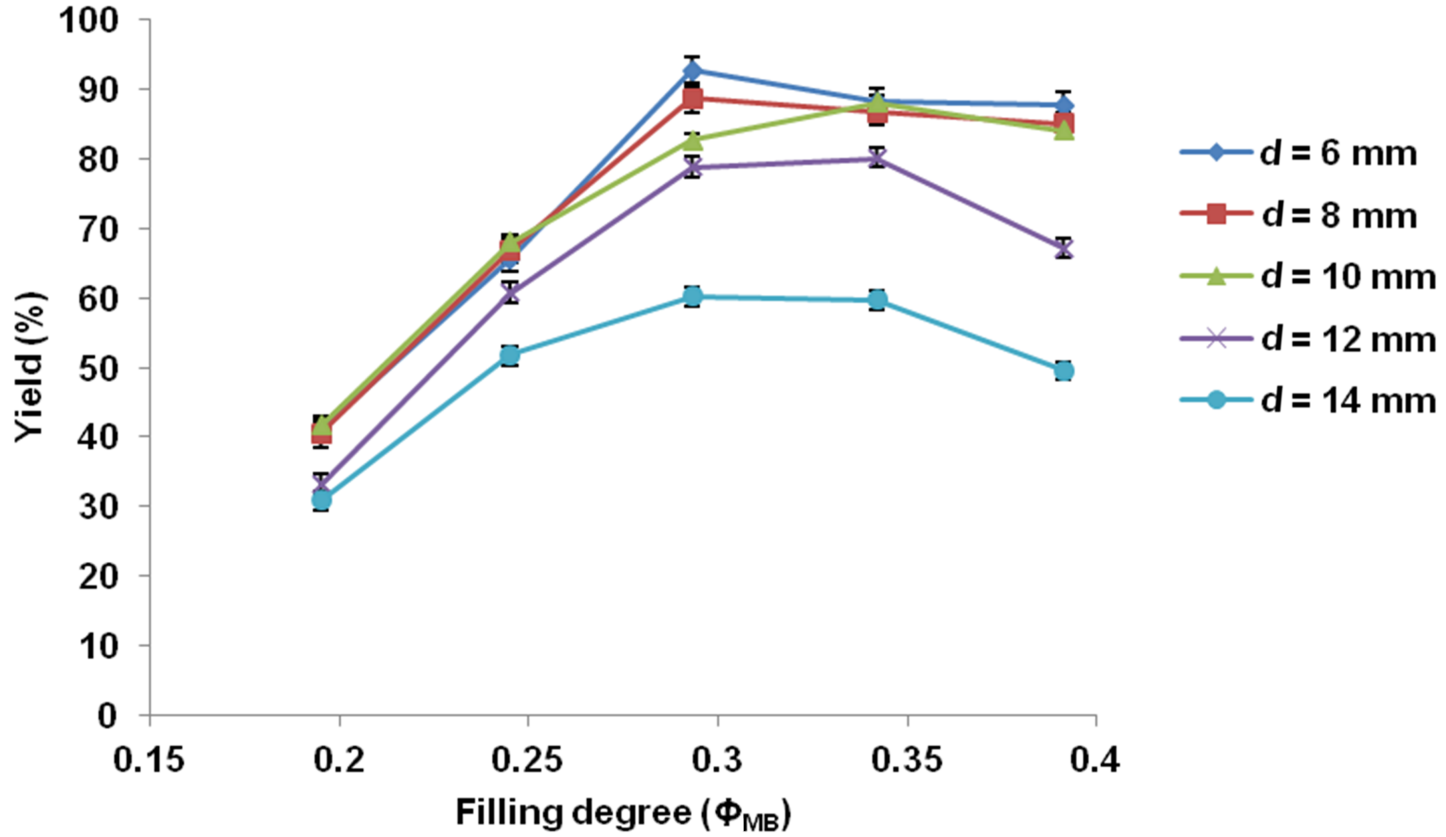
Influence of the milling ball filling degree with different size on the Heck reaction: **1a** (1.5 mmol), **2a** (2.25 mmol), Pd(OAc)_2_ (5 mol %), PPh_3_ (10 mol %), TEA (1.8 mmol), TBAB (5 mol %), and NaBr (10.0 g) were placed in 80 mL stainless steel vessel milling, milling at 800 rpm for 90 min.

Next, the combination of filling degree (Ф_MB_) and size of the milling balls (*d*_MB_) was investigated for further improving the product yield. As seen in Figure 3, the yield of **3aa** elevated sharply as the filling degree increased to around 0.3–0.35 for all types of the milling balls, and then decreased gradually due to milling vessel space limitations [24,61–62]. A maximum product yield (93%) was obtained by using 6 mm diameter milling balls (*Ф*_MB_ = 0.293), indicating small-size milling balls more beneficial for the reaction, which was in accordance with the previous studies reported by us [24] and others [57,63–64].

### Substrate scopes

**Scheme 5:**
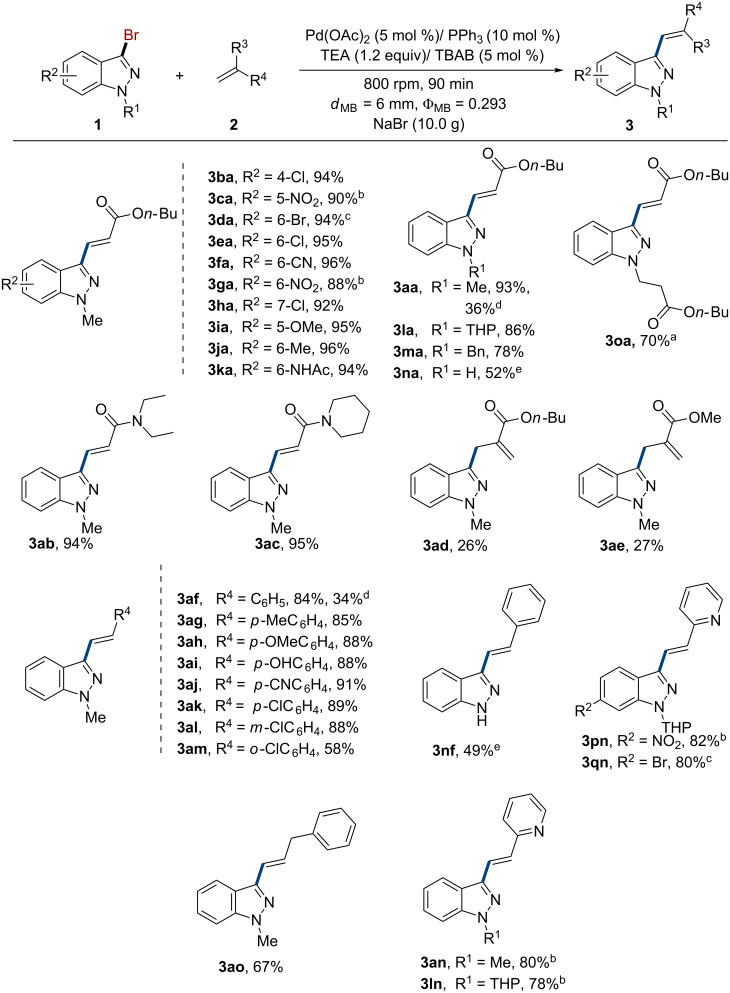
Examination of the substrate scope. Reaction conditions: **1** (1.5 mmol), **2** (2.25 mmol), Pd(OAc)_2_ (5 mol %), PPh_3_ (10 mol %), TEA (1.8 mmol), TBAB (5 mol %), and NaBr (10.0 g) were placed in an 80 mL stainless steel vessel along with 207 stainless-steel balls (*d*_MB_ = 6 mm, Ф_MB_ = 0.293), milling at 800 rpm for 90 min. ^a^*N*-Boc-3-bromoindazole was used as substrate. ^b^150 min. ^c^700 rpm. ^d^3-chloro-1-methyl-1*H*-indazole was used as substrate, *ν*_rot_ = 900 rpm. ^e^1,8-Bis(dimethylamino)naphthalene was used as base.

After a comprehensive study of the reaction pathway and the reaction conditions, an excellent product yield (93%) and selectivity (trace of **4a**) can be achieved. We then turned our efforts toward the investigation of the scope and limitations of the developed method with respect to a broad range of indazoles and olefins (Scheme 5). Pleasingly, neutral, electron-rich and electron-poor indazoles were perfectly tolerated in this reaction, affording the corresponding target product **3ba–ka** in high yields (88–96%) and excellent selectivity (the dehalogenated side-product **4** was not detected in most cases). Among which, strong electron-withdrawing substrates required long reaction times to achieve the desired results (**3ca, 3ga, 3pn**). It was suprising to find that substrate **1d** showed excellent site-selectivity under 700 rpm, giving 6-bromo-substituted product **3da** in 94% yield. Indazoles with *N*-Me, THP and Bn groups afforded good to excellent yields in the coupling reaction with *n*-butyl acrylate. However, the *N*-Boc substrate readily underwent removal of the protecting group [65], and resulted in the coupling product **3oa** (70%). Besides, *N*-unsubstituted indazole **1n** could also be tolerated in this reaction giving **3na** (52%) and **3nf** (49%) in moderate yield by using 1,8-bis(dimethylamino)naphthalene as a base. To our delight, 3-chloroindazole could also be activated in this reaction, giving the corresponding coupling products **3aa** and **3af** in considerable yields. The scope of the reaction with respect to the olefins was also extensively investigated and encompasses acrylates **2a**, acrylamides **2b** and **2c**, styrenes **2f–m** and 2-vinylpyridine (**2n**). In addition, even non-activated allylbenzene **2o** and disubstituted olefins **2d** and **2e** could participate in the reaction to deliver **3ao** (67%), **3ad** (26%) and **3ae** (27%). Finally, the steric hindrance of styrene was examined. Larger steric hindrance (**2m**) led to lower yield (58%) as compared with **2k** (89%) and **2l** (88%).

### Application in API synthesis

**Scheme 6:**
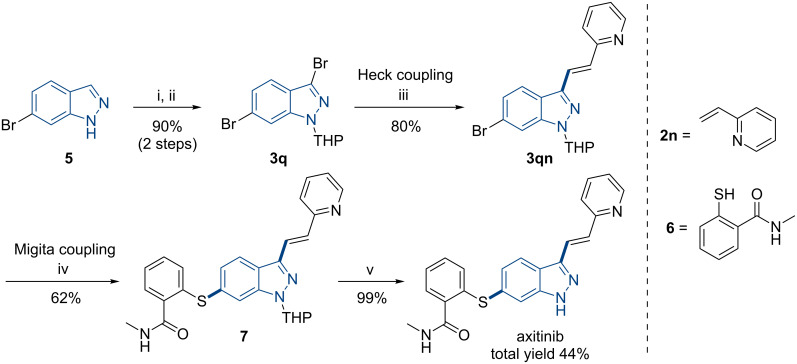
Synthesis of axitinib by mechanochemical Heck–Migita coupling. Reagents and conditions: (i) NBS, NaOH, silica gel (4.0 g), 173 stainless-steel balls (*d*_MB_ = 6 mm, Ф_MB_ = 0.245), 200 rpm, 30 min; (ii) CH_3_SO_3_H, dihydropyran, silica gel (4.0 g), 173 stainless-steel balls (*d*_MB_ = 6 mm, Ф_MB_ = 0.245), 200 rpm, 45 min; (iii) compound **2n**, Pd(OAc)_2_, PPh_3_, TBAB, TEA, NaBr (10.0 g), 207 stainless-steel balls (*d*_MB_ = 6 mm, Ф_MB_ = 0.293), 700 rpm, 90 min; (iv) compound **6**, Pd_2_(dba)_3_, Xantphos, Cs_2_CO_3_, silica gel (4.0 g), 207 stainless-steel balls (*d*_MB_ = 6 mm, Ф_MB_ = 0.293), 750 rpm, 50 min; (v) *p*-TsOH, silica gel (4.0 g), 207 stainless-steel balls (*d*_MB_ = 6 mm, Ф_MB_ = 0.293), 500 rpm, 45 min.

To demonstrate the broad utility of this method, a concise synthesis of axitinib, eutherapeutic drug for the treatment of renal cell carcinoma, was undertaken (Scheme 6). The reaction started from commercially available 6-bromo-1*H*-indazole (**5**), bromination of the 3-position and *N*-protection gave 3,6-dibromo-1-(tetrahydro-2*H*-pyran-2-yl)-1*H*-indazole (**3q**) in 90% yield. Next, sequential two-step Heck coupling and Migita coupling under ball milling conditions selectively afforded THP-axitinib **7**. Finally, deprotection of **7** with *p*-TsOH gave axitinib in a total yield of 44% (41% [66]). Comparing to previous synthetic procedures [35–36], this mechanochemical protocol provided a solvent-free, highly efficient and tractable alternative, and the residual Pd content in the axitinib was determined to be no more than 2 ppm by ICP analysis.

## Conclusion

In conclusion, a solvent-free, chemoselective Heck cross-coupling for the synthesis of 3-vinylindazoles has been developed by sophisticated tuning of the chemical and mechanical parameters under ball-milling conditions. The reaction pathway was comprehensively studied, revealing the bromide salts to play a dual role by not only suppressing the dehalogenation of 3-bromoindazoles but also assisting grinding, while a catalytic amount of TBAB was sufficient to stabilize Pd(0) and to promote the cross coupling. A series of non-activated indazoles and a broad scope of olefins were tolerated in the reaction giving high yields and excellent selectivity. Further application of this protocol was conducted in a total mechanosynthesis of axitinib in short reaction time and high efficiency. With this system, we hope to expand the pharmaceutical synthetic toolbox in mechanochemistry.

## Supporting Information

File 1 Reaction optimization studies, details of experimental procedures, characterization and copies of ^1^H and ^13^C NMR spectra of prepared compounds.
