# Supplementary material for: Bromide-assisted chemoselective Heck reaction of 3-bromoindazoles under high-speed ball-milling conditions: synthesis of axitinib
Source: Beilstein J Org Chem. 2018 Apr 6;14:786–95. doi: 10.3762/bjoc.14.66 (PMC5905281; doi:10.3762/bjoc.14.66)
Supplement: File 1 — Reaction optimization studies, details of experimental procedures, characterization and copies of 1H and 13C NMR spectra of prepared compounds. [file Beilstein_J_Org_Chem-14-786-s001.pdf]

Supporting Information

for

Bromide-assisted chemoselective Heck reaction of 3-bromoindazoles under high-speed ball-milling conditions: synthesis of axitinib

Jingbo Yu[1], Zikun Hong[2], Xinjie Yang[1], Yu Jiang[1], Zhijiang Jiang[1] and Weike Su[1,*]

Address: [1]Collaborative Innovation Center of Yangtze River Delta Region Green Pharmaceuticals, Zhejiang University of Technology, Hangzhou 310014, PR China and [2]College of Pharmaceutical Sciences, Zhejiang University of Technology, Hangzhou 310014, PR China

Email: Weike Su* - pharmlab@zjut.edu.cn

*Corresponding author

Reaction optimization studies, details of experimental procedures, characterization and copies of ^{1}H and ^{13}C NMR spectra of prepared compounds

Table of contents

1. Reaction optimization studies **S2**

2. Experimental section **S5**

3. Characterization data **S9**

4. Copies of ^{1}H NMR and ^{13}C NMR spectra **S26**

5. References **S63**

1. Reaction optimization studies

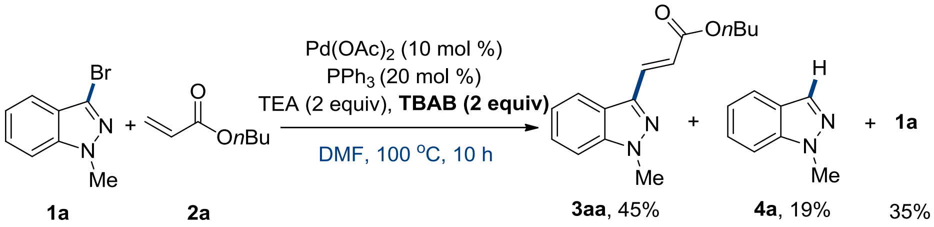

Scheme S1: Model Heck reaction of **1a** and **2a** under solvent heating conditions

Table S1: The influences of the additives on the reaction selectivity

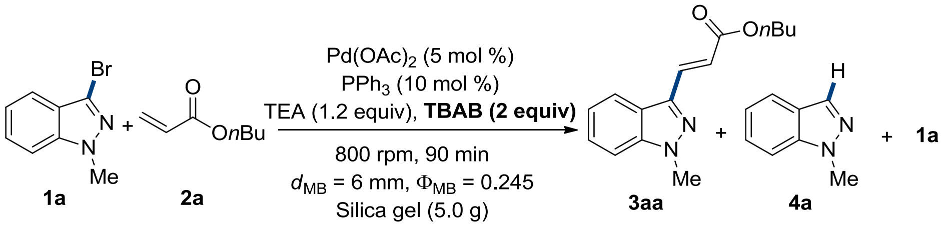

Entry	Modification from standard conditions	Yield (%) **3aa / 4a / 1a**
1	none (standard conditions[a])	54 / 19 / 26
2	without TBAB	14 / 38 / 45
3	TBAI instead of TBAB as additive	29 / 30 / 39
4	TBAC instead of TBAB as additive	29 / 32 / 38
5	NH_4Br instead of TBAB as additive	34 / 24 / 40
6	TEAB instead of TBAB as additive	36 / 21 / 41
7	CTAB instead of TBAB as additive	40 / 22 / 37
8	SDS instead of TBAB as additive	9 / 36 / 53
9	LiBr instead of TBAB as additive	38 / 17 / 43
10	KBr instead of TBAB as additive	39 / 17 / 43
11	NaBr instead of TBAB as additive	41 / 17 / 40
12	NaBr (10 g) instead of silica gel as grinding auxiliary	69 / trace / 29
13[b]	NaBr (10 g) instead of silica gel as grinding auxiliary	69 / trace / 29
**14[c]**	**NaBr (10 g) instead of silica gel as grinding auxiliary**	**69 / trace / 30**

[a]The reaction standard conditions: **1a** (1.5 mmol), **2a** (2.25 mmol), $Pd(OAc)_2$ (5 mol %), PPh_3 (10 mol %), TEA (1.8 mmol), TBAB (3.0 mmol), and silica gel (5.0 g) were placed in 80 mL stainless steel vessel along with 173 stainless-steel balls (d_{MB} = 6 mm, Φ_{MB} = 0.245), milling at 800 rpm for 90 min. [b]TBAB (0.15 mmol). [c]TBAB (0.075 mol).

Table S2: The influences of the milling time and rotation speed on the reaction

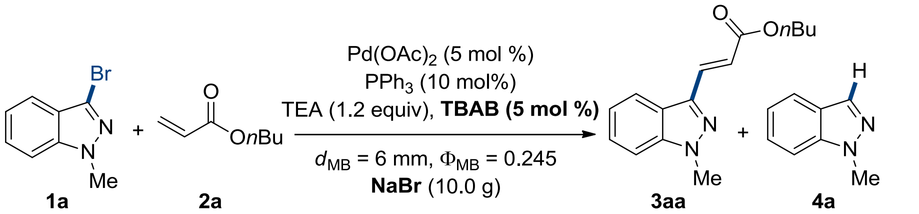

Rotation speed (rpm)	Time (min)	Yield of **3aa** (%)	Yield of **4a** (%)	Recovery rate of **1a** (%)
	60	19	0	79
600	90	28	0	71
	120	30	0	68
	60	36	trace	63
700	90	49	trace	50
	120	48	trace	49
	60	50	trace	45
**800**	**90**	**69**	**trace**	**30**
	120	65	trace	29
	60	56	2	39
900	90	66	2	28
	120	63	3	29
	60	54	2	38
1000	90	65	3	27
	120	62	3	27

The reaction conditions: **1a** (1.5 mmol), **2a** (2.25 mmol), $Pd(OAc)_2$ (5 mol %), PPh_3 (10 mol %), TEA (1.8 mmol), TBAB (5 mol %), and NaBr (10.0 g) were placed in 80 mL stainless steel vessel along with 173 stainless-steel balls (d_{MB} = 6 mm, Φ_{MB} = 0.245), milling at specific rotation speed for specific time.

Table S3: The influences of the milling ball filling degree and milling ball diameter on the reaction

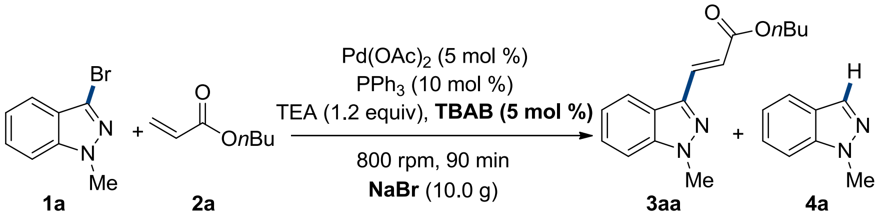

Diameter (d_{MB})	Filling degree (Φ_{MB})	The number of balls	Yield of **3aa** (%)	Yield of **4a** (%)	Recovery rate of **1a** (%)
	0.195	139	41	trace	57
	0.245	173	66	trace	30
**6 mm**	**0.293**	**207**	**93**	**trace**	**4**
	0.342	241	88	trace	10
	0.391	275	88	trace	10
	0.195	56	40	trace	58
	0.245	70	67	trace	31
8 mm	0.293	84	89	trace	7
	0.342	98	87	trace	12
	0.391	112	85	trace	13
	0.195	29	42	trace	58
	0.245	36	68	trace	30
10 mm	0.293	43	83	trace	16
	0.342	50	88	trace	10
	0.391	57	84	trace	14
	0.195	17	33	trace	65
	0.245	21	61	trace	37
12 mm	0.293	25	79	trace	19
	0.342	29	80	trace	18
	0.391	33	67	trace	30
	0.195	10	31	trace	68
	0.245	13	52	trace	48
14 mm	0.293	16	60	trace	38
	0.342	19	60	trace	39
	0.391	22	50	trace	49

The reaction conditions: **1a** (1.5 mmol), **2a** (2.25 mmol), $Pd(OAc)_2$ (5 mol%), PPh_3 (10 mol %), TEA (1.8 mmol), TBAB (5 mol %), and NaBr (10.0 g) were placed in 80 mL stainless steel vessel milling at 800 rpm for 90 min.

2. Experimental section

General information

All reagents were purchased from commercial sources and were used as received, unless otherwise indicated. All used silica gel is 300 mesh (Qingdao Haiyang Chemical Co., Ltd.). TLC analysis was performed using precoated glass plates. A high-energy ball mill (Fritsch GmbH Planet Mill pulverisette 7) was employed. All of the HSBM reactions were performed in 80 mL stainless-steel grinding vessels and milled with stainless-steel balls. The weight of a stainless steel ball (d_{MB} = 6 mm) is 0.870 g. Melting points (mp) were obtained on a digital melting point apparatus (OptiMelt MPA100) and are uncorrected. NMR spectra were recorded with a 500 (or 600) MHz spectrometer for ^{1}H and 126 (or 151) MHz for ^{13}C, and TMS was used as an internal standard. Mass spectra were recorded with a HRMS-ESI-Q-TOF (Agilent 6210 TOF LC/MS or micrOTOF-Q II) and a low-resolution MS instrument (Finnigan Trace DSQ) using an ESI ion source. For each compound purified by column chromatography, isocratic elution method was used.

General procedure for the Heck coupling reaction

A mixture of the substrate **1** (1.5 mmol), alkene **2** (2.25 mmol), $Pd(OAc)_2$ (0.075 mmol), PPh_3 (0.15 mmol), TEA (1.8 mmol), TBAB (0.075 mmol), and NaBr (10.0 g) was placed in a stainless-steel vessel, along with 207 stainless-steel balls (d_{MB} = 6 mm, Φ_{MB} = 0.293). The reaction mixture was then

ball-milled at 800 rpm for 90 min. At the end of the experiment, the reaction mixture was scratched off from the vessel and directly purified by column chromatography on silica gel (petroleum ether/EtOAc) to give the desired product **3**.

Preparation of 3,6-dibromo-1-(tetrahydro-2*H*-pyran-2-yl)-1*H*-indazole (3q). A mixture of the substrate **5** (10 mmol), *N*-bromosuccinimide (11 mmol), NaOH (12 mmol), and silica gel (4.0 g) was placed in an 80 mL stainless-steel vessel, along with 173 stainless-steel balls (d_{MB} = 6 mm, Φ_{MB} = 0.245). The reaction mixture was then ball-milled at 200 rpm for 30 min. At the end of the experiment, all the reaction mixture was scratched off from the vessel and directly purified by column chromatography on silica gel eluting with (petroleum ether/EtOAc 1:1) to afford 2.755 g (98%) of 3,6-dibromo-1*H*-indazole as a white solid. Next, a mixture of the substrate 3,6-dibromo-1*H*-indazole (9 mmol), 3,4-dihydropyran (10 mmol), CH_3SO_3H (2.5 mmol), and silica gel (4.0 g) was placed in a 80 mL stainless-steel vessel, along with 173 stainless-steel balls (d_{MB} = 6 mm, Φ_{MB} = 0.245). The reaction mixture was then ball-milled at 200 rpm for 45 min. At the end of the experiment, the reaction mixture was scratched off from the vessel and directly purified by column chromatography on silica gel eluting with (petroleum ether/EtOAc 10:1) to provide 2.947 g (92%) of **3q**.

Preparation of (*E*)-6-bromo-3-(2-(pyridin-2-yl)vinyl)-1-(tetrahydro-2*H*-pyran-2-yl)-1*H*-indazole (3qn).

A mixture of the **3q** (3.581 g, 10 mmol), alkenes **2n** (1.296 mL, 12 mmol), $Pd(OAc)_2$ (112.0 mg, 0. 5 mmol), PPh_3 (262.0 mg, 1.0 mmol), TEA (1.380 mL, 12 mmol), TBAB (161.0 mg, 0. 5 mmol), and NaBr (15.0 g) was placed in a 120 mL stainless-steel vessel, along with 310 stainless-steel balls (d_{MB} = 6 mm, Φ_{MB} = 0.293). The reaction mixture was then ball-milled with rotational speed of 700 rpm for 90 min. At the end of the experiment, the reaction mixture was scratched off from the vessel and directly purified by column chromatography on silica gel (petroleum ether/EtOAc 10:1) to give 2.945 g (77% [1]) of **3qn**.

Preparation of (*E*)-*N*-methyl-2-((3-(2-(pyridin-2-yl)vinyl)-1-(tetrahydro-2*H*-pyran-2-yl)-1*H*-indazol-6-yl)thio)benzamide (7).

A mixture of **3qn** (2 or 10 mmol), **6** (2.4 or 12 mmol), $Pd_2(dba)_3$ (0.01 or 0.05 mmol), Xantphos (0.02 or 0.1 mmol), $CsCO_3$ (2.4 or 12 mmol), and silica gel (4.0 or 6.0 [1] g) was placed in an 80 mL (120 mL) stainless-steel vessel, along with 207 (310) stainless-steel balls (d_{MB} = 6 mm, Φ_{MB} = 0.293). The reaction mixture was then ball-milled at 750 rpm for 50 min. At the end of the experiment, the reaction mixture was scratched off from the vessel and directly purified by column chromatography on silica gel (petroleum ether/EtOAc 6:1) to afford 0.580 or 2.807 [1] g (62% or 60% [1]) of **7**.

Preparation of (*E*)-*N*-methyl-2-((3-(2-(pyridin-2-yl)vinyl)-1*H*-indazol-6-yl)thio)benzamide (axitinib).

A mixture of **7** (5 mmol), *p*-TsOH (20 mmol), and silica gel (4.0 g) was placed in a 80 mL stainless-steel vessel, along with 207 stainless-steel balls (d_{MB} = 6 mm, Φ_{MB} = 0.293). The reaction mixture was then ball-milled at 500 rpm for 45 min. At the end of the experiment, the reaction mixture was scratched off from the vessel and directly purified by column chromatography on silica gel eluting with (petroleum ether/EtOAc 1:1) to provide 1.917 g (99%) of **axitinib**.

3. Characterization data

Butyl (*E*)-3-(1-methyl-1*H*-indazol-3-yl)acrylate (3aa). White solid (359 mg, 93% yield). mp: 42–44 °C (petroleum ether/EtOAc 15:1). ^{1}H NMR (500 MHz, $CDCl_3$) *δ* 7.99 (d, *J* = 16.0 Hz, 1H), 7.94 (d, *J* = 8.5 Hz, 1H), 7.42–7.47 (m, 2H), 7.25–7.28 (m, 1H), 6.76 (d, *J* = 16.0 Hz, 1H), 4.24 (t, *J* = 7.0 Hz, 2H), 4.11 (s, 3H), 1.68–1.74 (m, 2H), 1.43–1.50 (m, 2H), 0.98 (t, *J* = 7.0 Hz, 3H). ^{13}C NMR (151 MHz, $CDCl_3$) *δ* 167.3, 141.3, 139.7, 135.8, 126.7, 122.6, 122.1, 120.7, 119.0, 109.6, 64.4, 35.9, 30.8, 19.3, 13.8. MS (ESI) (*m/z*): $[M+H]^+$ 259.1. HRMS (ESI) (*m/z*): $[M+H]^+$ calcd for $C_{15}H_{19}N_2O_2$, 259.1441; found 259.1449.

Butyl (*E*)-3-(4-chloro-1-methyl-1*H*-indazol-3-yl)acrylate (3ba). White solid (412 mg, 94% yield). mp: 63–66 °C (petroleum ether/EtOAc 20:1). ^{1}H NMR (500 MHz, $CDCl_3$) *δ* 7.92 (d, *J* = 16.2 Hz, 1H), 7.80 (d, *J* = 8.5 Hz, 1H), 7.38 (d, *J* = 7.5 Hz, 1H), 7.14 (t, *J* = 8.0 Hz, 1H), 6.75 (d, *J* = 16.2 Hz, 1H), 4.43 (s, 3H), 4.24 (t, *J* = 6.5 Hz, 2H), 1.68–1.74 (m, 2H), 1.42–1.50 (m, 2H), 0.97 (t, *J* = 7.5 Hz, 3H). ^{13}C NMR (126 MHz, $CDCl_3$) *δ* 167.0, 139.5, 137.5, 134.6, 127.7, 125.4, 122.7, 119.7, 119.3, 116.7, 64.5, 39.6, 30.8, 19.2, 13.7. MS (ESI) (*m/z*): $[M+H]^+$ 293.1. HRMS (ESI) (*m/z*): $[M+H]^+$ calcd for $C_{15}H_{18}ClN_2O_2$, 293.1051; found 293.1052.

Butyl (*E*)-3-(1-methyl-5-nitro-1*H*-indazol-3-yl)acrylate (3ca). Yellow solid (410 mg, 90% yield). mp: 117–119 °C (petroleum ether/EtOAc 8:1). ^{1}H NMR

(600 MHz, $CDCl_3$) δ 8.90 (s, 1H), 8.33 (dd, J = 9.0, 2.0 Hz, 1H), 7.94 (d, J = 16.2 Hz, 1H), 7.50 (d, J = 9.0 Hz, 1H), 6.83 (d, J = 16.2 Hz, 1H), 4.27 (t, J = 6.6 Hz, 2H), 4.18 (s, 3H), 1.71–1.76 (m, 2H), 1.45–1.51 (m, 2H), 0.99 (t, J = 7.2 Hz, 3H). ^{13}C NMR (151 MHz, $CDCl_3$) δ 166.6, 143.1, 142.9, 142.4, 133.7, 121.9, 121.6, 121.4, 118.3, 110.0, 64.8, 36.4, 30.7, 19.2, 13.8. MS (ESI) (*m/z*): $[M+H]^+$ 304.1. HRMS (ESI) (*m/z*): $[M+H]^+$ calcd for $C_{15}H_{18}N_3O_4$, 304.1292; found 304.1283.

Butyl (*E*)-3-(6-bromo-1-methyl-1*H*-indazol-3-yl)acrylate (3da). White solid (474 mg, 94% yield). mp: 76–78 °C (petroleum ether/EtOAc 9:1). ^{1}H NMR (600 MHz, $CDCl_3$) δ 7.79 (d, J = 16.2 Hz, 1H), 7.59 (d, J = 9.0 Hz, 1H), 7.47 (s, 1H), 7.41 (d, J = 9.0 Hz, 1H), 6.55 (d, J = 16.2 Hz, 1H), 4.24 (t, J = 6.6 Hz, 2H), 4.07 (s, 3H), 1.69–1.73 (m, 2H), 1.43–1.49 (m, 2H), 0.98 (t, J = 7.2 Hz, 3H). ^{13}C NMR (151 MHz, $CDCl_3$) δ 166.8, 144.2, 141.3, 134.0, 124.5, 120.9, 120.2, 120.0, 119.8, 109.9, 64.6, 36.1, 30.8, 19.2, 13.8. MS (ESI) (*m/z*): $[M+H]^+$ 337.3. HRMS (ESI) (*m/z*): $[M+H]^+$ calcd for $C_{15}H_{18}ClN_2O_2$, 337.0546; found 337.0534.

Butyl (*E*)-3-(6-chloro-1-methyl-1*H*-indazol-3-yl)acrylate (3ea). Yellow solid (417 mg, 95% yield). mp: 51–54 °C (petroleum ether/EtOAc 20:1). ^{1}H NMR (600 MHz, $CDCl_3$) δ 7.92 (d, J = 16.2 Hz, 1H), 7.83 (d, J = 8.4 Hz, 1H), 7.42 (s, 1H), 7.22 (d, J = 8.4 Hz, 1H), 6.72 (d, J = 16.2 Hz, 1H), 4.24 (t, J = 6.6 Hz, 2H),

4.07 (s, 3H), 1.68–1.73 (m, 2H), 1.42–1.49 (m, 2H), 0.97 (t, *J* = 7.2 Hz, 3H). ^{13}C NMR (151 MHz, $CDCl_3$) δ 167.0, 141.7, 139.9, 135.0, 133.2, 123.0, 121.6, 121.1, 119.7, 109.4, 64.5, 36.0, 30.8, 19.2, 13.8. MS (ESI) (*m/z*): $[M+H]^+$ 293.1. HRMS (ESI) (*m/z*): $[M+H]^+$ calcd for $C_{15}H_{18}ClN_2O_2$, 293.1051; found 293.1063.

Butyl (*E*)-3-(6-cyano-1-methyl-1*H*-indazol-3-yl)acrylate (3fa). White solid (408 mg, 96% yield). mp: 115–117 °C (petroleum ether/EtOAc 20:1). ^{1}H NMR (600 MHz, $CDCl_3$) δ 8.02 (d, *J* = 8.4 Hz, 1H), 7.94 (d, *J* = 16.2 Hz, 1H), 7.82 (s, 1H), 7.47 (d, *J* = 8.4 Hz, 1H), 6.77 (d, *J* = 16.2 Hz, 1H), 4.25 (t, *J* = 6.6 Hz, 2H), 4.16 (s, 3H), 1.69–1.74 (m, 2H), 1.43-1.49 (m, 2H), 0.98 (t, *J* = 7.2 Hz, 3H). ^{13}C NMR (151 MHz, $CDCl_3$) δ 166.8, 140.2, 140.0, 134.1, 124.5, 124.0, 121.9, 120.5, 118.9, 114.9, 110.1, 64.7, 36.3, 30.8, 19.2, 13.8. MS (ESI) (*m/z*): $[M+H]^+$ 284.1. HRMS (ESI) (*m/z*): $[M+H]^+$ calcd for $C_{16}H_{18}N_3O_2$, 284.1394; found 284.1406.

Butyl (*E*)-3-(1-methyl-6-nitro-1*H*-indazol-3-yl)acrylate (3ga). Yellow solid (398 mg, 88% yield). mp: 139–142 °C (petroleum ether/EtOAc 8:1). ^{1}H NMR (600 MHz, DMSO) δ 8.78 (d, *J* = 1.8 Hz, 1H), 8.37 (d, *J* = 8.4 Hz, 1H), 8.02 (dd, *J* = 9.0, 1.8 Hz, 1H), 7.86 (d, *J* = 16.2 Hz, 1H), 6.80 (d, *J* = 16.2 Hz, 1H), 4.24 (s, 3H), 4.19 (t, *J* = 6.6 Hz, 2H), 1.64–1.68 (m, 2H), 1.38–1.44 (m, 2H), 0.94 (t, *J* = 7.2 Hz, 3H). ^{13}C NMR (151 MHz, DMSO) δ 166.5, 146.5, 140.4, 139.5,

134.5, 125.1, 122.4, 119.9, 116.7, 108.2, 64.4, 37.0, 30.7, 19.2, 14.1. MS (ESI) (*m/z*): $[M+Na]^+$ 326.1. HRMS (ESI) (*m/z*): $[M+Na]^+$ calcd for $C_{15}H_{17}N_3NaO_4$, 326.1111; found 326.1098.

Butyl (*E*)-3-(7-chloro-1-methyl-1*H*-indazol-3-yl)acrylate (3ha). White solid (403 mg, 92% yield). mp: 59–61 °C (petroleum ether/EtOAc 20:1). ^{1}H NMR (600 MHz, $CDCl_3$) δ 8.46 (d, J = 16.2 Hz, 1H), 7.29–7.30 (m, 2H), 7.19-7.20 (m, 1H), 6.86 (d, J = 16.2 Hz, 1H), 4.23 (t, J = 6.6 Hz, 2H), 4.10 (s, 3H), 1.68–1.73 (m, 2H), 1.42–1.49 (m, 2H), 0.97 (t, J = 7.2 Hz, 3H). ^{13}C NMR (151 MHz, $CDCl_3$) δ 167.2, 142.3, 139.1, 134.1, 127.0 (2C), 122.4, 120.7, 119.3, 108.3, 64.3, 36.2, 30.8, 19.2, 13.8. MS (ESI) (*m/z*): $[M+H]^+$ 293.1. HRMS (ESI) (*m/z*): $[M+H]^+$ calcd for $C_{15}H_{18}ClN_2O_2$, 293.1051; found 293.1052.

Butyl (*E*)-3-(5-methoxy-1-methyl-1*H*-indazol-3-yl)acrylate (3ia). Yellow oil (410 mg, 95% yield) (petroleum ether/EtOAc 16:1). ^{1}H NMR (600 MHz, $CDCl_3$) δ 7.97 (d, J = 16.2 Hz, 1H), 7.32 (d, J = 9.0 Hz, 1H), 7.21 (s, 1H), 7.11 (d, J = 9.0 Hz, 1H), 6.67 (d, J = 16.2 Hz, 1H), 4.24 (t, J = 6.6 Hz, 2H), 4.08 (s, 3H), 3.90 (s, 3 H), 1.69–1.74 (m, 2H), 1.43–1.49 (m, 2H), 0.98 (t, J = 7.2 Hz, 3H). ^{13}C NMR (151 MHz, $CDCl_3$) δ 167.5, 155.8, 138.8, 137.2, 135.8, 123.2, 118.9, 117.9, 110.6, 99.9, 64.4, 55.8, 36.1, 30.8, 22.0, 19.3, 13.8. MS (ESI) (*m/z*): $[M+H]^+$ 289.2. HRMS (ESI) (*m/z*): $[M+H]^+$ calcd for $C_{16}H_{21}N_2O_3$, 289.1547; found 289.1556.

Butyl (*E*)-3-(1,6-dimethyl-1*H*-indazol-3-yl)acrylate (3ja). Yellow oil (393 mg, 96% yield) (petroleum ether/EtOAc 18:1). ^{1}H NMR (600 MHz, $CDCl_3$) δ 7.95 (d, J = 16.2 Hz, 1H), 7.81 (d, J = 8.4 Hz, 1H), 7.19 (s, 1H), 7.10 (d, J = 8.4 Hz, 1H), 6.72 (d, J = 16.2 Hz, 1H), 4.23 (t, J = 6.6 Hz, 2H), 4.06 (s, 3H), 2.52 (s, 3H), 1.68–1.73 (m, 2H), 1.43–1.49 (m, 2H), 0.97 (t, J = 7.2 Hz, 3H). ^{13}C NMR (151 MHz, $CDCl_3$) δ 167.3, 141.9, 139.5, 137.2, 136.0, 124.3, 120.7, 120.3, 118.8, 109.0, 64.4, 35.8, 30.8, 22.0, 19.3, 13.8. MS (ESI) (*m/z*): $[M+H]^+$ 273.2. HRMS (ESI) (*m/z*): $[M+H]^+$ calcd for $C_{16}H_{21}N_2O_2$, 273.1598; found 273.1604.

Butyl (*E*)-3-(6-acetamido-1-methyl-1*H*-indazol-3-yl)acrylate (3ka). White solid (445 mg, 94% yield). mp: 103–106 °C (petroleum ether/EtOAc 3:1). ^{1}H NMR (500 MHz, $CDCl_3$) δ 8.24 (s, 1 H), 7.92 (d, J = 16.5 Hz, 1H), 7.83 (brs, 1H), 7.78 (d, J = 8.5 Hz, 1H), 6.91 (dd, J = 8.5, 1.5 Hz, 1H), 6.69 (d, J = 16.5 Hz, 1H), 4.24 (t, J = 7.0 Hz, 2H), 4.05 (s, 3H), 2.25 (s, 3H), 1.67–1.73 (m, 2H), 1.42–1.50 (m, 2H), 0.97 (t, J = 7.5 Hz, 3H). ^{13}C NMR (151 MHz, $CDCl_3$) δ 169.1, 167.4, 141.9, 139.5, 137.1, 135.8, 120.9, 119.1, 118.9, 115.7, 99.8, 64.6, 35.9, 30.8, 24.7, 19.2, 13.8. MS (ESI) (*m/z*): $[M+H]^+$ 316.2. HRMS (ESI) (*m/z*): $[M+H]^+$ calcd for $C_{17}H_{22}N_3O_3$, 316.1656; found 316.1663.

Butyl (*E*)-3-(1-(tetrahydro-2*H*-pyran-2-yl)-1*H*-indazol-3-yl)acrylate (3la). White solid (422 mg, 86% yield). mp: 59–62 °C (petroleum ether/EtOAc 18:1).

^{1}H NMR (600 MHz, $CDCl_3$) δ 8.01 (d, J = 16.2 Hz, 1H), 7.94 (d, J = 8.4 Hz, 1H), 7.63 (d, J = 8.4 Hz, 1H), 7.44 (t, J = 7.8 Hz, 1H), 7.28 (d, J = 7.8 Hz, 1H), 6.80 (d, J = 16.2 Hz, 1H), 5.76 (dd, J = 9.0, 2.4 Hz, 1H), 4.24 (t, J = 6.6 Hz, 2H), 4.00–4.05 (m, 1H), 3.73–3.78 (m, 1H), 2.55–2.61 (m, 1H), 2.17–2.19 (m, 1H), 2.05–2.09 (m, 1H), 1.66–1.80 (m, 5H), 1.43–1.49 (m, 2H), 0.98 (t, J = 7.2 Hz, 3H). ^{13}C NMR (151 MHz, $CDCl_3$) δ 167.2, 140.9, 140.6, 136.0, 126.9, 123.2, 122.6, 120.7, 120.0, 110.8, 85.6, 67.5, 64.5, 30.8, 29.3, 25.1, 22.4, 19.3, 13.8. MS (ESI) (*m/z*): $[M+Na]^+$ 351.5. HRMS (ESI) (*m/z*): $[M+Na]^+$ calcd for $C_{19}H_{24}N_2NaO_3$, 351.1679; found 351.1661.

Butyl (*E*)-3-(1-benzyl-1*H*-indazol-3-yl)acrylate (3ma). Yellow oil (390 mg, 78% yield) (petroleum ether/EtOAc 20:1). ^{1}H NMR (600 MHz, $CDCl_3$) δ 8.02 (d, J = 16.2 Hz, 1H), 7.95 (d, J = 8.4 Hz, 1H), 7.36–7.39 (m, 2H), 7.27–7.31 (m, 4H), 7.21 (d, J = 7.2 Hz, 2H), 6.79 (d, J = 16.2 Hz, 1H), 5.61 (s, 2H), 4.24 (t, J = 6.6 Hz, 2H), 1.68–1.73 (m, 2H), 1.43–1.49 (m, 2H), 0.97 (t, J = 7.2 Hz, 3H). ^{13}C NMR (151 MHz, $CDCl_3$) δ 167.2, 141.0, 140.2, 136.1, 135.9, 128.9 (2C), 128.0, 127.2 (2C), 126.9, 123.0, 122.2, 120.9, 119.4, 110.0, 64.5, 53.4, 30.8, 19.3, 13.8. MS (ESI) (*m/z*): $[M+Na]^+$ 357.4. HRMS (ESI) (*m/z*): $[M+Na]^+$ calcd for $C_{21}H_{22}N_2NaO_2$, 357.1573; found 357.1556.

Butyl (*E*)-3-(1*H*-indazol-3-yl)acrylate (3na). Yellow solid (189 mg, 52% yield). mp: 125–128°C (petroleum ether/EtOAc 15:1). ^{1}H NMR (600 MHz,

$CDCl_3$) δ 8.03 (d, J = 16.2 Hz, 1H), 7.98 (d, J = 8.4 Hz, 1H), 7.54 (d, J = 8.4 Hz, 1H), 7.45 (t, J = 7.2 Hz, 1H), 7.29 (t, J = 7.2 Hz, 1 H), 6.82 (d, J = 16.2 Hz, 1H), 4.26 (t, J = 6.6 Hz, 2H), 1.70–1.75 (m, 2H), 1.44–1.50 (m, 2H), 0.98 (t, J = 7.2 Hz, 3H). ^{13}C NMR (151 MHz, $CDCl_3$) δ 167.3, 141.7, 141.5, 135.9, 127.3, 122.4, 121.7, 120.6, 120.1, 110.4, 64.6, 30.8, 19.2, 13.8. MS (ESI) (*m/z*): $[M+H]^+$ 245.1. HRMS (ESI) (*m/z*): $[M+H]^+$ calcd for $C_{14}H_{17}N_2O_2$, 245.1285; found 245.1289.

Butyl (*E*)-3-(1-(3-butoxy-3-oxopropyl)-1*H*-indazol-3-yl)acrylate (3oa). Yellow oil (391 mg, 70% yield) (petroleum ether/EtOAc 20:1). ^{1}H NMR (600 MHz, $CDCl_3$) δ 7.97 (d, J = 16.2 Hz, 1H), 7.92 (d, J = 8.4 Hz, 1H), 7.52 (d, J = 8.4 Hz, 1H), 7.44 (t, J = 7.8 Hz, 1H), 7.26 (t, J = 7.8 Hz, 1H), 6.76 (d, J = 16.2 Hz, 1H), 4.68 (t, J = 6.6 Hz, 2H), 4.24 (t, J = 6.6 Hz, 2H), 4.04 (t, J = 6.6 Hz, 2H), 3.01 (t, J = 6.6 Hz, 2H), 1.69–1.74 (m, 2H), 1.44–1.53 (m, 4H), 1.24–1.29 (m, 2H), 0.98 (t, J = 7.2 Hz, 3H), 0.87 (t, J = 7.2 Hz, 3H). ^{13}C NMR (151 MHz, $CDCl_3$) δ 171.1, 167.2, 141.0, 140.3, 135.7, 126.8, 122.6, 122.2, 120.7, 119.3, 109.8, 64.9, 64.5, 44.5, 34.3, 30.8, 30.5, 19.2, 19.0, 13.8, 13.6. MS (ESI) (*m/z*): $[M+Na]^+$ 395.4. HRMS (ESI) (*m/z*): $[M+Na]^+$ calcd for $C_{21}H_{28}N_2NaO_4$, 395.1941; found 395.1953.

(*E*)-*N,N*-Diethyl-3-(1-methyl-1*H*-indazol-3-yl)acrylamide (3ab). Yellow solid (361 mg, 94% yield). mp: 106–109 °C (lit 103–105 °C [2]) (petroleum

ether/EtOAc 4:1). ^{1}H NMR (600 MHz, $CDCl_3$) δ 8.02 (d, J = 15.6 Hz, 1H), 7.88 (d, J = 8.4 Hz, 1H), 7.40–7.45 (m, 2H), 7.22–7.26 (m, 2H, including d, J = 15.6 Hz, 1H), 4.11 (s, 3H), 3.54 (q, J = 7.2 Hz, 4H), 1.22–1.31 (m, 6H). ^{13}C NMR (151 MHz, $CDCl_3$) δ 165.8, 141.1, 140.3, 132.3, 126.6, 123.0, 121.6, 120.3, 118.2, 109.4, 42.4, 41.1, 35.8, 15.2,13.3. MS (ESI) (*m/z*): $[M+Na]^+$ 280.2. HRMS (ESI) (*m/z*): $[M+Na]^+$ calcd for $C_{15}H_{19}N_3NaO$, 280.1420; found 280.1407.

(*E*)-3-(1-Methyl-1*H*-indazol-3-yl)-1-(piperidin-1-yl)prop-2-en-1-one (3ac). Yellow solid (383 mg, 95% yield). mp: 107–110 °C (petroleum ether/EtOAc 4:1). ^{1}H NMR (500 MHz, $CDCl_3$) δ 7.97 (d, J = 15.5 Hz, 1H), 7.88 (d, J = 8.0 Hz, 1H), 7.39–7.45 (m, 2H), 7.33 (d, J = 15.5 Hz, 1H), 7.22–7.26 (m, 1H), 4.11 (s, 3H), 3.68 (brs, 4H), 1.67–1.71 (m, 2H), 1.61–1.65 (m, 4H). ^{13}C NMR (151 MHz, CD_3OD) δ 165.9, 141.2, 139.8, 132.7, 126.7, 122.2, 121.8, 120.0, 117.7, 109.5, 46.9, 43.3, 34.6, 26.5, 25.5, 24.2. MS (ESI) (*m/z*): $[M+Na]^+$ 292.4. HRMS (ESI) (*m/z*): $[M+Na]^+$ calcd for $C_{16}H_{19}N_3NaO$, 292.1420; found 292.1406.

Butyl 2-((1-methyl-1*H*-indazol-3-yl)methyl)acrylate (3ad). Yellow oil (107 mg, 26% yield) (petroleum ether/EtOAc 15:1). ^{1}H NMR (600 MHz, $CDCl_3$) δ 7.66 (d, J = 7.8 Hz, 1H), 7.34–7.39 (m, 2H), 7.11 (t, J = 7.2 Hz, 1H), 6.28 (s, 1H), 5.52 (s, 1H), 4.16 (t, J = 6.6 Hz, 3H), 4.03 (s, 3H), 4.00 (s, 2H), 1.60–1.64 (m, 2H), 1.32–1.38 (m, 2H), 0.90 (t, J = 7.2 Hz, 3H). ^{13}C NMR (151 MHz,

$CDCl_3$) δ 165.9, 140.9, 139.9, 137.3, 125.3, 125.2, 121.8, 119.6, 118.9, 107.9, 63.7, 34.2, 29.6, 28.4, 18.1, 12.7. MS (ESI) (*m/z*): $[M+Na]^+$ 295.1. HRMS (ESI) (*m/z*): $[M+Na]^+$ calcd for $C_{16}H_{20}N_2NaO_2$, 295.1417; found 295.1405.

Methyl 2-((1-methyl-1*H*-indazol-3-yl)methyl)acrylate (3ae). Yellow oil (92 mg, 27% yield) (petroleum ether/EtOAc 15:1). ^{1}H NMR (600 MHz, $CDCl_3$) δ 7.66 (d, *J* = 8.4 Hz, 1H), 7.34–7.39 (m, 2H), 7.11 (t, *J* = 7.2 Hz, 1H), 6.28 (s, 1H), 5.54–5.55 (m, 1H), 4.03 (s, 3H), 4.00 (s, 2H), 3.77 (s, 3H). ^{13}C NMR (151 MHz, $CDCl_3$) δ 166.3, 140.8, 140.0, 125.6, 125.2, 121.7, 119.6, 118.9, 107.9, 60.0, 34.2, 28.3. MS (ESI) (*m/z*): $[M+Na]^+$ 253.2. HRMS (ESI) (*m/z)*: $[M+Na]^+$ calcd for $C_{13}H_{14}N_2NaO_2$, 253.0947; found 253.0944.

(*E*)-1-Methyl-3-styryl-1*H*-indazole (3af). Yellow solid (295 mg, 84% yield). mp: 73–76 °C (lit 72–75 °C [3]) (petroleum ether/EtOAc 12:1). ^{1}H NMR (500 MHz, $CDCl_3$) δ 7.98 (d, *J* = 8.5 Hz, 1H), 7.57 (d, *J* = 7.5 Hz, 2H), 7.48 (d, *J* =16.5 Hz, 1H), 7.32–7.45 (m, 5H, including d, *J* = 16.5 Hz, 1H), 7.26 (t, *J* = 7.0 Hz, 1H), 7.20 (t, *J* = 7.0 Hz, 1H), 4.03 (s, 3H). ^{13}C NMR (126 MHz, $CDCl_3$) δ 142.1, 141.2, 137.4, 130.2, 128.7 (2C), 127.7, 126.5, 126.4 (2C), 122.0, 121.0, 120.9, 120.0, 109.2, 35.5. MS (ESI) (*m/z*): $[M+H]^+$ 235.3. HRMS (ESI) (*m/z*): $[M+H]^+$ calcd for $C_{16}H_{15}N_2$, 235.1230; found 235.1221.

(*E*)-1-Methyl-3-(4-methylstyryl)-1*H*-indazole (3ag). Yellow solid (315 mg, 85% yield). mp: 92–93 °C (petroleum ether/EtOAc 18:1). ^{1}H NMR (600 MHz, $CDCl_3$) *δ* 8.01 (d, *J* = 8.4 Hz, 1H), 7.45–7.50 (m, 3H, including d, *J* = 16.2, 1H), 7.36–7.43 (m, 3H, including d, *J* = 16.2, 1H), 7.17–7.24 (m, 3H), 4.08 (s, 3H), 2.37 (s, 3H). ^{13}C NMR (151 MHz, $CDCl_3$) *δ* 142.3, 141.3, 137.7, 134.7, 130.3, 129.5 (2C), 126.5, 126.4 (2C), 122.0, 121.1, 120.9, 119.0, 109.2, 35.5, 21.3. MS (ESI) (*m/z*): $[M+H]^+$ 249.3. HRMS (ESI) (*m/z*): $[M+H]^+$ calcd for $C_{17}H_{17}N_2$, 249.1386; found 249.1397.

(*E*)-3-(4-Methoxystyryl)-1-methyl-1*H*-indazole (3ah). Yellow solid (349 mg, 88 % yield). mp: 57–59 °C (lit 56-58 °C [4]) (petroleum ether/EtOAc 12:1). ^{1}H NMR (600 MHz, $CDCl_3$) *δ* 8.00 (d, *J* = 7.8 Hz,1H), 7.52 (d, *J* = 9.0 Hz, 2H), 7.36–7.47 (m, 3H, including d, *J* = 16.8 Hz, 1H), 7.29 (d, *J* = 16.8 Hz, 1H), 7.21 (t, *J* = 7.2 Hz, 1H), 6.92 (d, *J* = 8.4 Hz, 2H), 4.07 (s, 3H), 3.84 (s, 3H). ^{13}C NMR (151 MHz, CDCl3) δ 159.4, 142.3, 141.2, 130.2, 129.9, 127.7 (2C), 126.4, 121.9, 121.0, 120.7, 117.8, 114.2 (2C), 109.2, 55.3, 35.5. MS (ESI) (*m/z*): $[M+H]^+$ 265.3. HRMS (ESI) (*m/z*): $[M+Na]^+$, calcd for $C_{17}H_{16}N_2NaO$, 287.1155; found 287.1170.

(*E*)-4-(2-(1-Methyl-1*H*-indazol-3-yl)vinyl)phenol (3ai). Yellow solid (331 mg, 88% yield). mp: 214–216 °C (petroleum ether/EtOAc 4:1). ^{1}H NMR (600 MHz, DMSO) *δ* 9.61 (s, 1H), 8.14 (d, *J* = 7.8 Hz, 1H), 7.62 (d, *J* = 8.4 Hz, 1H), 7.53

(d, *J* = 8.4 Hz, 3H), 7.39–7.44 (m, 2H, including d, *J* = 16.8 Hz, 1H), 7.29 (d, *J* = 16.8 Hz, 1H), 7.21 (t, *J* = 7.8 Hz, 1H), 6.80 (d, *J* = 8.4 Hz, 2H), 4.04 (s, 3H). ^{13}C NMR (600 MHz, DMSO) δ 157.9, 141.9, 141.3, 129.9, 128.7, 128.3 (2C), 126.7, 121.7, 121.3, 121.1, 117.1, 116.1 (2C), 110.3, 35.8. MS (ESI) (*m/z*): $[M+H]^+$ 251.1. HRMS (ESI) (m/z): $[M+H]^+$ calcd for $C_{16}H_{15}N_2O$, 251.1179; found 251.1168.

(*E*)-4-(2-(1-Methyl-1*H*-indazol-3-yl)vinyl)benzonitrile (3aj). White solid (353 mg, 91% yield). mp:157–159 °C (petroleum ether/EtOAc 9:1). ^{1}H NMR (600 MHz, $CDCl_3$) δ 7.98 (d, *J* = 7.8 Hz, 1H), 7.62–7.66 (m, 4H), 7.53 (d, *J* = 16.2 Hz, 1H), 7.41–7.47 (m, 3H, including d, *J* = 16.2 Hz, 1H), 7.26 (t, *J* = 7.2 Hz, 1H), 4.11 (s, 3H). ^{13}C NMR (151 MHz, $CDCl_3$) δ 141.9, 141.3, 141.1, 132.5 (2C), 127.8, 126.7 (2C), 123.6, 122.1, 121.4, 120.6, 119.1, 110.5, 109.5, 35.7. MS (ESI) (*m/z*): $[M+H]^+$ 260.2. HRMS (ESI) (*m/z*): $[M+H]^+$ calcd for $C_{17}H_{14}N_3$, 260.1182; found 260.1191.

(*E*)-3-(4-Chlorostyryl)-1-methyl-1*H*-indazole (3ak). Yellow solid (359 mg, 89% yield). mp: 102–103 °C (petroleum ether/EtOAc 14:1). ^{1}H NMR (600 MHz, $CDCl_3$) δ 7.98 (d, *J* = 7.8 Hz, 1H), 7.50 (d, *J* = 8.4 Hz, 2H), 7.37–7.45 (m, 4H, including d, *J* = 16.8 Hz, 2H), 7.34 (d, *J* = 8.4 Hz, 2H), 7.23 (t, *J* = 7.2 Hz, 1H), 4.08 (s, 3H). ^{13}C NMR (151 MHz, $CDCl_3$) δ 141.8, 141.3, 136.0, 133.3, 128.9 (2C), 128.8, 127.6 (2C), 126.6, 122.0, 121.1, 120.9, 120.6, 109.3, 35.6. MS

(ESI) (*m/z*): $[M+H]^+$ 269.1. HRMS (ESI) (*m/z*): $[M+H]^+$ calcd for $C_{16}H_{14}ClN_2$, 269.0840; found 269.0852.

(*E*)-3-(3-Chlorostyryl)-1-methyl-1*H*-indazole (3al). Yellow solid (353 mg, 88% yield). mp: 98–99 °C (petroleum ether/EtOAc 20:1). ^{1}H NMR (600 MHz, $CDCl_3$) δ 7.98 (d, *J* = 8.4 Hz, 1H), 7.56 (s, 1H), 7.38–7.45 (m, 5H), 7.30 (t, *J* = 7.8 Hz, 1H), 7.22–7.25 (m, 2H), 4.08 (s, 3H). ^{13}C NMR (151 MHz, $CDCl_3$) δ 141.6, 141.3, 139.4, 134.7, 129.9, 128.5, 127.6, 126.6, 126.3, 124.6, 122.0, 121.4, 121.2, 120.8, 109.3, 35.6. MS (ESI) (*m/z*): $[M+H]^+$ 269.4. HRMS (ESI) (*m/z*): $[M+H]^+$ calcd for $C_{16}H_{14}ClN_2$, 269.0840; found 269.0827.

(*E*)-3-(2-Chlorostyryl)-1-methyl-1*H*-indazole (3am). Yellow solid (232 mg, 58% yield). mp: 87–89 °C (petroleum ether/EtOAc 20:1). ^{1}H NMR (600 MHz, $CDCl_3$) δ 8.07 (d, *J* = 8.4 Hz, 1H), 7.88 (d, *J* = 16.2 Hz, 1H), 7.77 (d, *J* = 8.4 Hz, 1H), 7.39–7.45 (m, 4H, including d, *J* = 16.2 Hz, 1H), 7.24–7.30 (m, 2H), 7.21 (td, *J* = 8.4, 1.8 Hz, 1H), 4.09 (s, 3H). ^{13}C NMR (151 MHz, $CDCl_3$) δ 142.1, 141.4, 135.5, 133.4, 129.9, 128.6, 127.0, 126.6, 126.3, 126.1, 123.0, 121.9, 121.3, 121.2, 109.3, 35.6. MS (ESI) (*m/z*): $[M+H]^+$ 269.2. HRMS (ESI) (*m/z*): $[M+H]^+$ calcd for $C_{16}H_{14}ClN_2$, 269.0840; found 269.0825 .

(*E*)-1-Methyl-3-(2-(pyridin-2-yl)vinyl)-1*H*-indazole (3an). Yellow soild (281 mg, 80% yield). mp: 86–89 °C (petroleum ether/EtOAc 5:1). ^{1}H NMR (600 MHz, $CDCl_3$) δ 8.64 (d, *J* = 4.8 Hz, 1H), 8.08 (d, *J* = 8.4 Hz, 1H), 7.93 (d, *J*

=16.2 Hz, 1H), 7.69 (td, *J* = 7.8, 1.8 Hz, 1H), 7.58 (d, *J* = 16.2 Hz, 1H), 7.48 (d, *J* = 7.8 Hz, 1H), 7.40–7.45 (m, 2H), 7.24 (t, *J* = 7.2 Hz, 1H), 7.16–7.18 (m, 1H), 4.11 (s, 3H). ^{13}C NMR (151 MHz, $CDCl_3$) δ 155.8, 149.7, 141.5, 141.3, 136.6, 129.3, 126.5, 123.7, 122.5, 122.1, 121.9, 121.2, 121.0, 109.3, 35.7. MS (ESI) (*m/z*): $[M+H]^+$ 236.4. HRMS (ESI) (*m/z*): $[M+H]^+$ calcd for $C_{15}H_{14}N_3$, 236.1182; found 236.1177.

(*E*)-1-Methyl-3-(3-phenylprop-1-en-1-yl)-1*H*-indazole (3ao). Yellow oil (250 mg, 67% yield) (petroleum ether/EtOAc 16:1). ^{1}H NMR (600 MHz, $CDCl_3$) δ 7.71 (d, *J* = 7.8 Hz, 1H), 7.33–7.38 (m, 4H), 7.25–7.28 (m, 2H, d, *J* = 16.2 Hz, 1H), 7.19 (t, *J* = 7.2 Hz, 1H),7.10 (t, *J* = 7.2 Hz, 1H), 6.59 (d, *J* = 16.2 Hz, 1H), 6.45–6.50 (m, 1H), 4.03 (s, 3H), 3.91 (dd, *J* = 6.6, 1.2 Hz, 2H). ^{13}C NMR (151 MHz, $CDCl_3$) δ 143.0, 141.1, 137.4, 131.5, 128.5 (2C), 127.3, 127.2, 126.3, 126.2 (2C), 122.8, 120.7, 119.9, 109.0, 35.3, 31.2. MS (ESI) (*m/z*): $[M+H]^+$ 249.4. HRMS (ESI) (*m/z*): $[M+Na]^+$ calcd for $C_{17}H_{16}N_2Na$, 271.1206; found 271.1218.

(*E*)-3-(2-(Pyridin-2-yl)vinyl)-1-(tetrahydro-2*H*-pyran-2-yl)-1*H*-indazole (3ln). Yellow solid (358 mg, 78% yield). mp: 99–102 °C (petroleum ether/EtOAc 10:1). ^{1}H NMR (600 MHz, $CDCl_3$) δ 8.63 (d, *J* = 4.2 Hz, 1H), 8.07 (d, *J* = 8.4 Hz, 1H), 7.92 (d, *J* = 16.2 Hz, 1H), 7.69 (t, *J* = 7.8 Hz, 1H), 7.58–7.64 (m, 2H, including d, *J* = 16.2 Hz, 1H), 7.51 (d, *J* = 7.8 Hz, 1H), 7.43

(t, *J* = 7.8 Hz, 1H), 7.25–7.27 (m, 1H), 7.16–7.18 (m, 1H), 5.75 (dd, *J* = 9.6, 2.4 Hz, 1H), 4.04–4.09 (m, 1H), 3.74–3.79 (m, 1H), 2.59–2.66 (m, 1H), 2.18–2.22 (m, 1H), 2.08–2.10 (m, 1H), 1.75–1.83 (m, 2H), 1.67–1.68 (m, 1H). ^{13}C NMR (151 MHz, $CDCl_3$) *δ* 155.8, 149.6, 142.5, 140.8, 136.6, 130.3, 126.7, 124.0, 123.1, 122.2, 121.9, 121.7, 121.1, 110.4, 85.5, 67.6, 29.5, 25.1, 22.6. MS (ESI) (*m/z*): $[M+H]^+$ 306.1. HRMS (ESI) (*m/z*): $[M+H]^+$ calcd for $C_{19}H_{20}N_3O$, 306.1601; found 306.1597.

(*E*)-3-Styryl-1*H*-indazole (3nf). Yellow solid (161 mg, 49% yield). mp: 165–169 °C (lit 170 °C [5]) (petroleum ether/EtOAc 15:1). ^{1}H NMR (600 MHz, $CDCl_3$) δ 8.05 (d, *J* = 8.4 Hz, 1H), 7.60 (d, *J* = 7.8 Hz, 2H), 7.55 (d, *J* = 16.8 Hz, 1H), 7.46–7.52 (m, 1H, including d, *J* = 16.8 Hz, 1H), 7.38–7.43 (m, 3H), 7.30 (t, *J* = 7.2 Hz, 1H), 7.25 (t, *J* = 7.2 Hz, 1H). ^{13}C NMR (151 MHz, $CDCl_3$) *δ* 144.0, 141.6, 137.2, 131.3, 128.8 (2C), 128.0, 127.0, 126.6 (2C), 121.4, 121.3, 120.9, 120.1, 110.3. MS (ESI) (*m/z*): $[M+H]^+$ 221.1. HRMS (ESI) (*m/z*): $[M+H]^+$ calcd for $C_{15}H_{13}N_2$, 221.1073; found 221.1072.

(*E*)-6-Nitro-3-(2-(pyridin-2-yl)vinyl)-1-(tetrahydro-2*H*-pyran-2-yl)-1*H*-indazole (3pn). Yellow solid (430 mg, 82% yield). mp: 177–180 °C (petroleum ether/EtOAc 8:1). ^{1}H NMR (600 MHz, $CDCl_3$) *δ* 8.65 (d, *J* = 4.8 Hz, 1H), 8.56 (d, *J* = 1.8 Hz, 1H), 8.09–8.14 (m, 2H), 7.92 (d, *J* = 16.2 Hz, 1H), 7.71 (td, *J* = 7.8, 1.8 Hz, 1H), 7.62 (d, *J* = 16.2 Hz, 1H), 7.48 (d, *J* = 7.8 Hz, 1H), 7.20–7.22

(m, 1H), 5.83 (dd, *J* = 9.0, 3.0 Hz, 1H), 4.05–4.09 (m, 1H), 3.80–3.84 (m, 1H), 2.56–2.62 (m, 1H), 2.14–2.22 (m, 2H), 1.77–1.86 (m, 2H), 1.71–1.75 (m, 1H). ^{13}C NMR (151 MHz, $CDCl_3$) δ 155.0, 149.8, 146.6, 142.7, 139.5, 136.7, 131.4, 126.3, 122.6, 122.3, 122.2, 121.6, 116.5, 107.4, 86.1, 67.6, 29.4, 25.0, 22.2. MS (ESI) (*m/z*): $[M+H]^+$ 350.8. HRMS (ESI) (*m/z*): $[M+H]^+$ calcd for $C_{19}H_{19}N_4O_3$, 351.1452; found 351.1461.

(*E*)-6-Bromo-3-(2-(pyridin-2-yl)vinyl)-1-(tetrahydro-2*H*-pyran-2-yl)-1*H*-indazole (3qn). yellow solid (460 mg, 80% yield): mp 128–131 °C (petroleum ether/EtOAc 10:1). ^{1}H NMR (600 MHz, $CDCl_3$) δ 8.63 (d, *J* = 4.8 Hz, 1H), 7.78 (d, *J* = 16.2 Hz, 1H), 7.68–7.71 (m, 2H), 7.58 (d, *J* = 8.4 Hz, 1H), 7.52 (d, *J* = 8.4 Hz, 1H), 7.42 (d, *J* = 7.8 Hz, 1H), 7.28 (d, *J* = 16.2 Hz, 1H), 7.17–7.19 (m, 1H), 5.69 (dd, *J* = 9.3, 2.7 Hz, 1H), 4.03–4.06 (m, 1H), 3.73–3.77 (m, 1H), 2.51–2.58 (m, 1H), 2.14–2.17 (m, 1H), 2.07–2.10 (m, 1H), 1.72–1.80 (m, 2H), 1.65–1.70 (m, 1H). ^{13}C NMR (151 MHz, $CDCl_3$) δ 155.2, 149.7, 141.1, 136.7, 136.6, 132.6, 129.4, 124.4, 122.4 (2C), 122.0, 120.9, 120.6, 109.6, 85.7, 67.5, 29.3, 25.0, 22.4. MS (ESI) (*m/z*): $[M+H]^+$ 384.1. HRMS (ESI) (*m/z*): $[M+H]^+$ calcd for $C_{19}H_{19}BrN_3O$, 384.0706; found 384.0712.

1-Methyl-1*H*-indazole (4a). White solid. mp: 58–60 ^{o}C (lit 57–59 °C [6]) (petroleum ether/EtOAc 10:1). ^{1}H NMR (600 MHz, $CDCl_3$) δ 7.98 (s, 1H), 7.72 (d, *J* = 7.8 Hz, 1H), 7.37 (d, *J* = 3.6 Hz, 2H), 7.11–7.16 (m, 1H), 4.06 (s, 3H).

^{13}C NMR (151 MHz, $CDCl_3$) δ 139.9, 132.7, 126.2, 124.0, 121.1, 120.4, 108.9, 35.5. MS (ESI) (*m/z*): $[M+H]^+$ 133.1.

3,6-Dibromo-1-(tetrahydro-2*H*-pyran-2-yl)-1*H*-indazole (3q). White solid: mp 119–121 °C (petroleum ether/EtOAc 15:1). ^1H NMR (500 MHz, $CDCl_3$) δ 7.78 (s, 1H), 7.46 (d, *J* = 9.0 Hz, 1H), 7.33 (dd, *J* = 8.5, 1.5 Hz, 1H), 5.62 (dd, *J* = 9.0, 2.5 Hz, 1H), 4.00–4,04 (m, 1H), 3.70–3.76 (m, 1H), 2.45–2.53 (m, 1H), 2.11–2.17 (m, 1H), 2.03–2.09 (m, 1H), 1.63–1.76 (m, 3H). ^{13}C NMR (151 MHz, $CDCl_3$) δ 141.2, 125.7, 123.4, 122.5, 122.1, 121.6, 113.6, 85.8, 67.4, 29.3, 25.0, 22.2. MS (ESI) (*m/z*): $[M+H]^+$ 358.9. HRMS (ESI) (*m/z*): $[M+H]^+$ calcd for $C_{12}H_{13}Br_2N_2O$, 358.9389; found 358.9386.

(*E*)-*N*-Methyl-2-((3-(2-(pyridin-2-yl)vinyl)-1-(tetrahydro-2*H*-pyran-2-yl)-1*H*-indazol-6-yl)thio)benzamide (7). Yellow solid: mp 140–143 °C (lit 142–143 °C [7, 8]) (petroleum ether/EtOAc 6:1). ^1H NMR (600 MHz, $CDCl_3$) δ 8.62 (d, *J* = 4.8 Hz, 1H), 7.78 (d, *J* = 16.8 Hz, 2H), 7.70 (t, *J* = 7.2 Hz, 1H), 7.62 (d, *J* = 8.4 Hz, 1H), 7.57 (d, *J* = 7.2 Hz, 1H), 7.48 (d, *J* = 8.4 Hz, 1H), 7.42 (d, *J* = 7.8 Hz, 1H), 7.27 (d, *J* = 16.2 Hz, 1H), 7.16–7.22 (m, 3H), 7.13 (d, *J* = 7.8 Hz, 1H), 6.80 (s, 1H), 5.75 (dd, *J* = 9.0, 2.4 Hz, 1H), 4.04 (d, *J* = 11.4 Hz, 1H), 3.76–3.80 (m, 1H), 3.04 (d, *J* = 10.8 Hz, 3H), 2.49–2.55 (m, 1H), 2.09–2.15 (m, 2H), 1.67–1.79 (m, 3H). ^{13}C NMR (151 MHz, $CDCl_3$) δ 168.8, 155.3, 149.7, 141.3, 137.3, 136.7, 136.4, 136.1, 133.9, 132.7, 130.9, 130.5, 129.2, 128.5,

126.7, 122.3, 121.0, 120.8, 110.0, 86.1, 67.5, 29.3, 26.8, 25.0, 22.3. MS (ESI) (*m/z*): $[M+H]^+$ 471.2. HRMS (ESI) (*m/z*): $[M+H]^+$ calcd for $C_{27}H_{27}N_4O_2S$, 471.1849; found 471.1863.

(*E*)-*N*-Methyl-2-((3-(2-(pyridin-2-yl)vinyl)-1*H*-indazol-6-yl)thio)benzamide (axitinib). White solid: mp 224–227 °C (lit 222–225 °C [7, 8]) (petroleum ether/EtOAc 1:1). ^{1}H NMR (600 MHz, DMSO) *δ* 13.73 (s, 1H), 8.60 (d, *J* = 4.2 Hz, 1H), 8.52–8.54 (m, 1H), 7.79–7.87 (m, 3H, including d, *J* = 16.2 Hz, 1H), 7.56–7.59 (m, 2H), 7.52–7.54 (m, 1H), 7.46 (d, *J* = 8.4 Hz, 1H), 7.42 (d, *J* = 16.2 Hz, 1H), 7.27–7.29 (m, 1H), 7.18–7.21 (m, 2H), 6.69–6.72 (m, 1H), 2.85 (d, *J* = 4.2 Hz, 3H). ^{13}C NMR (151 MHz, DMSO) *δ* 168.2, 155.3, 150.0, 142.5, 137.3, 136.8, 136.3, 135.7, 135.0, 132.6, 130.7, 129.4, 128.2, 127.9, 125.6, 125.0, 123.0, 122.9, 120.6, 120.3, 110.7, 26.7. MS (ESI) (*m/z*): $[M+H]^+$ 387.2. HRMS (ESI) (*m/z*): $[M+H]^+$ calcd for $C_{22}H_{19}N_4OS$, 387.1274; found 387.1273.

4. Copies of ^{1}H NMR and ^{13}C NMR Spectra

Butyl (*E*)-3-(1-methyl-1*H*-indazol-3-yl)acrylate (3aa).

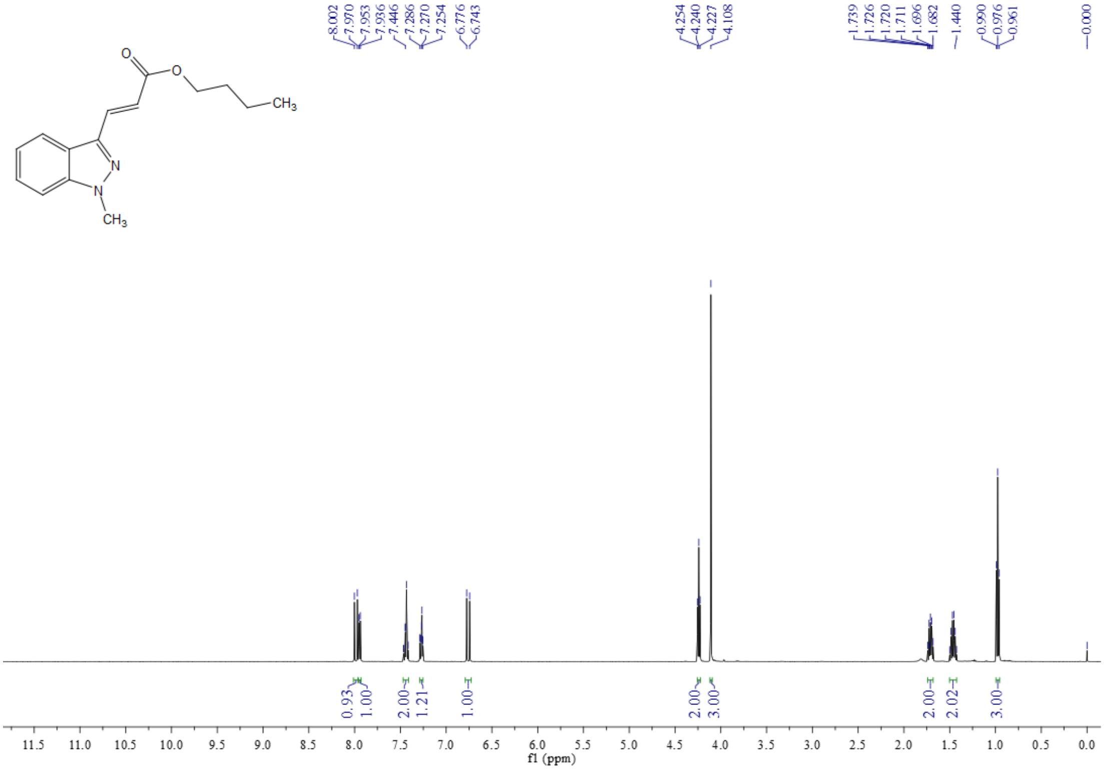

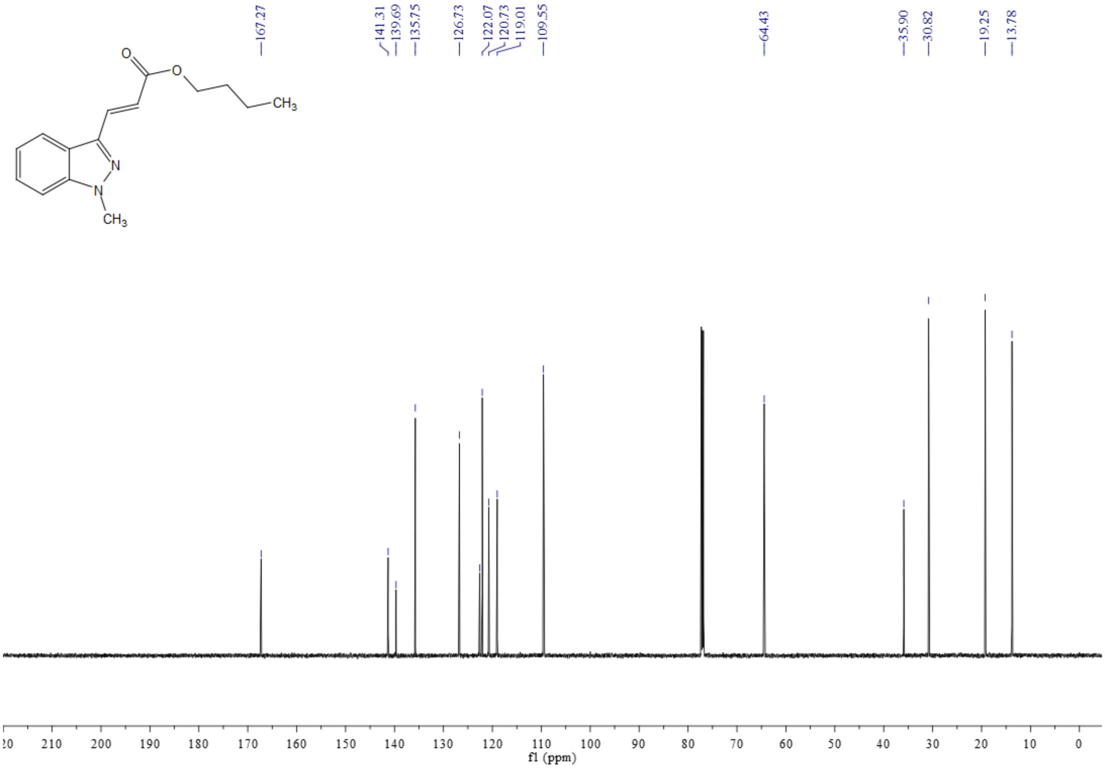

Butyl (*E*)-3-(4-chloro-1-methyl-1*H*-indazol-3-yl)acrylate (3ba).

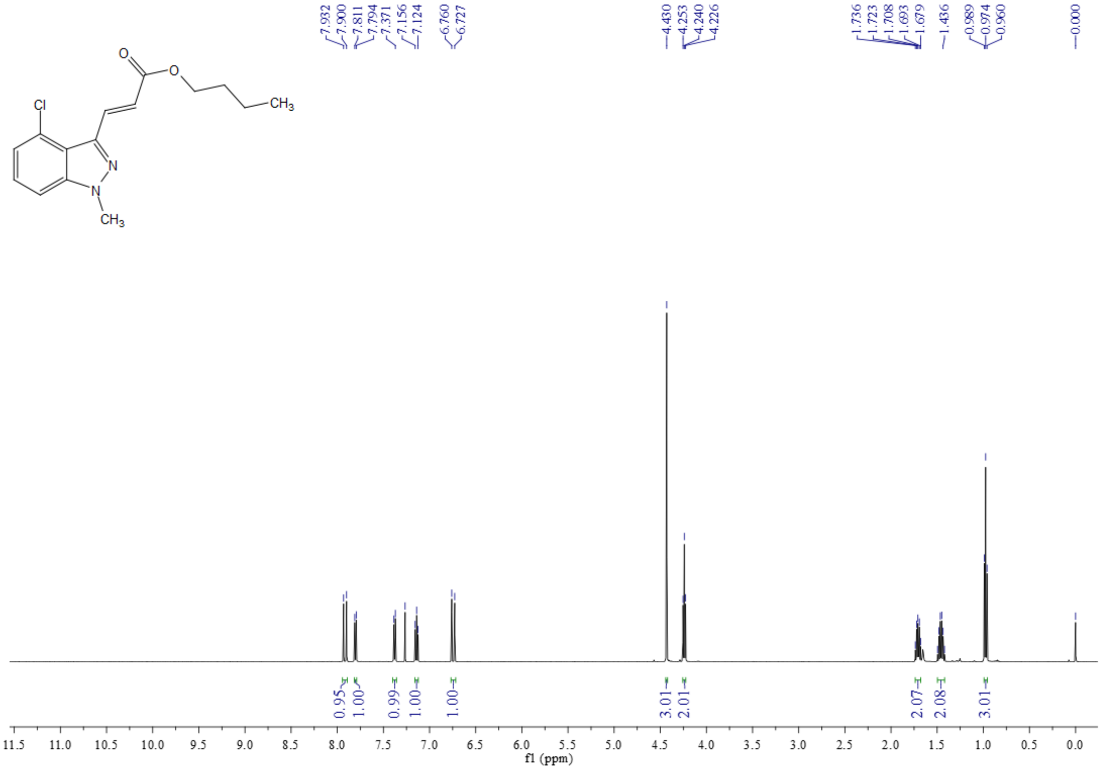

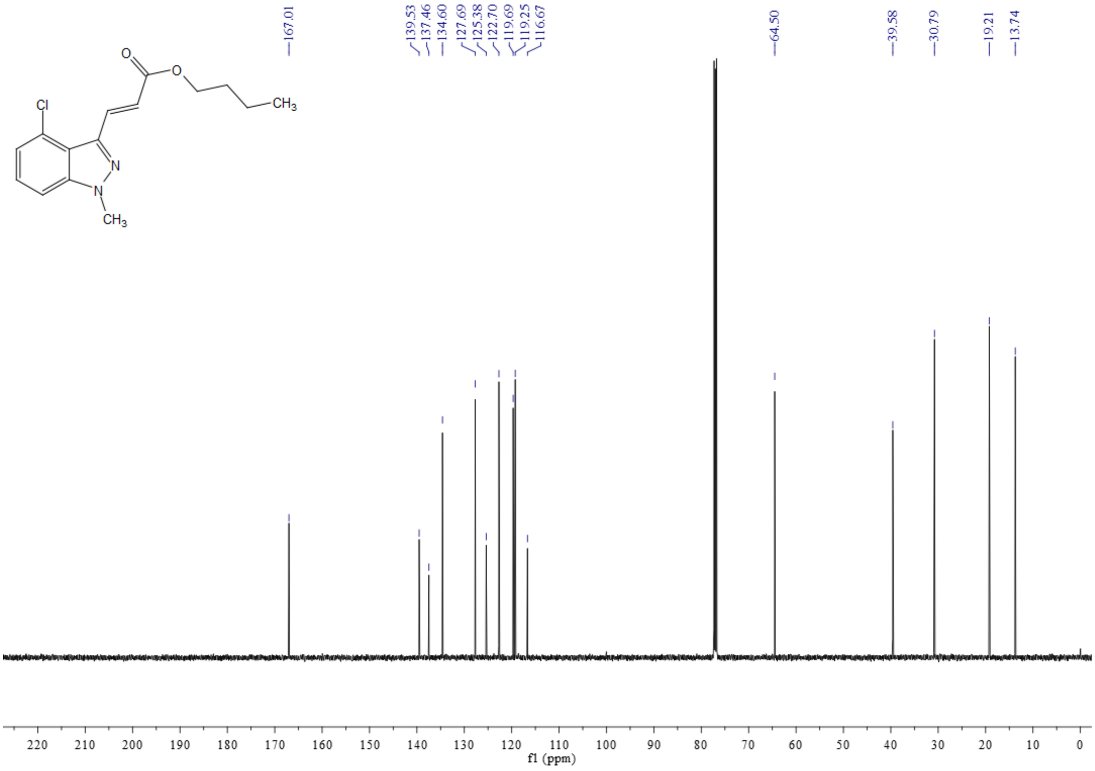

Butyl (*E*)-3-(1-methyl-5-nitro-1*H*-indazol-3-yl)acrylate (3ca).

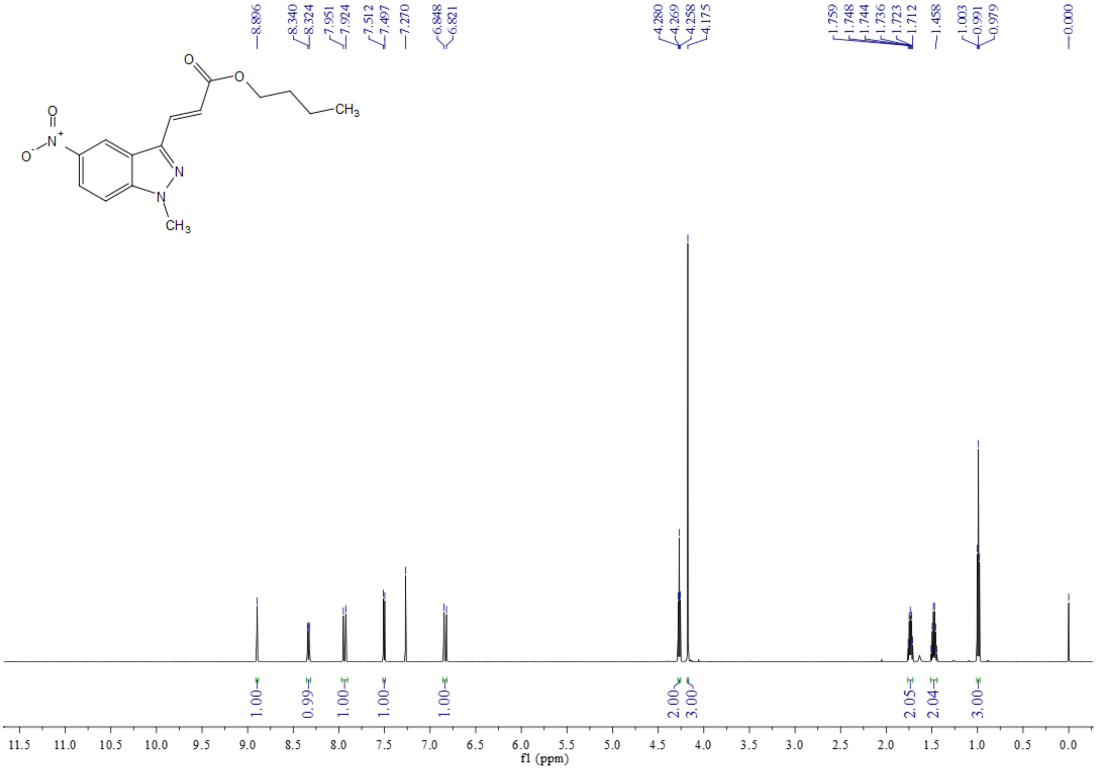

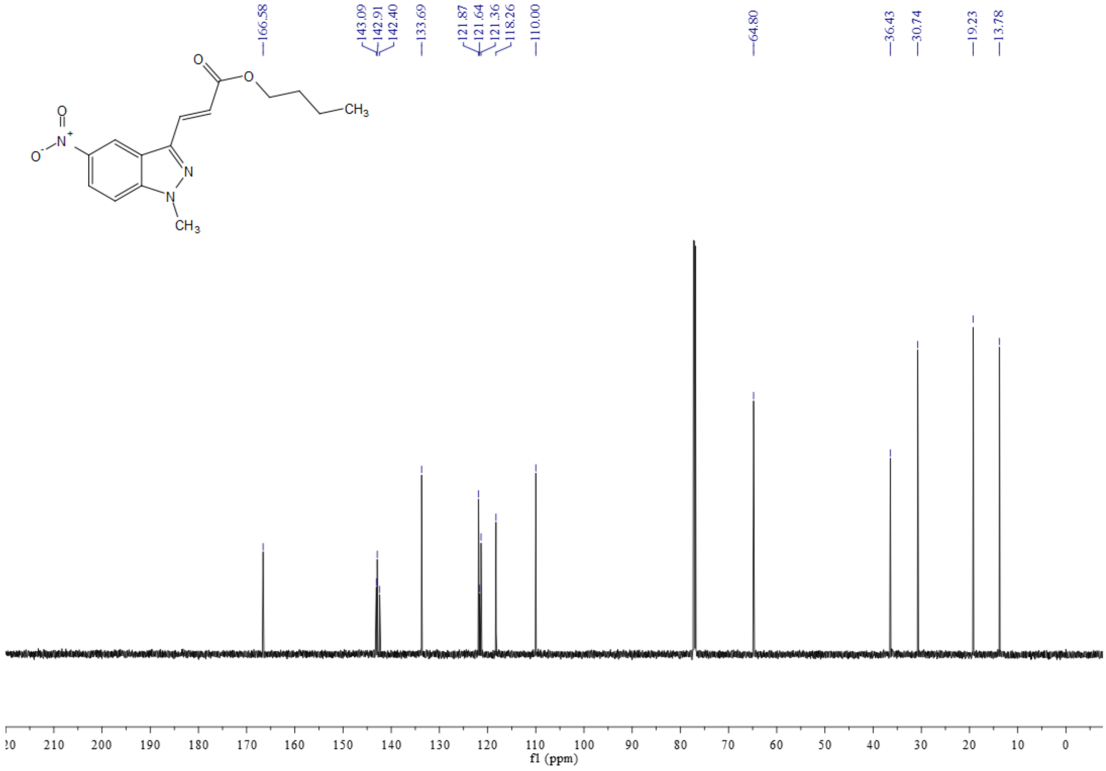

Butyl (*E*)-3-(6-bromo-1-methyl-1*H*-indazol-3-yl)acrylate (3da).

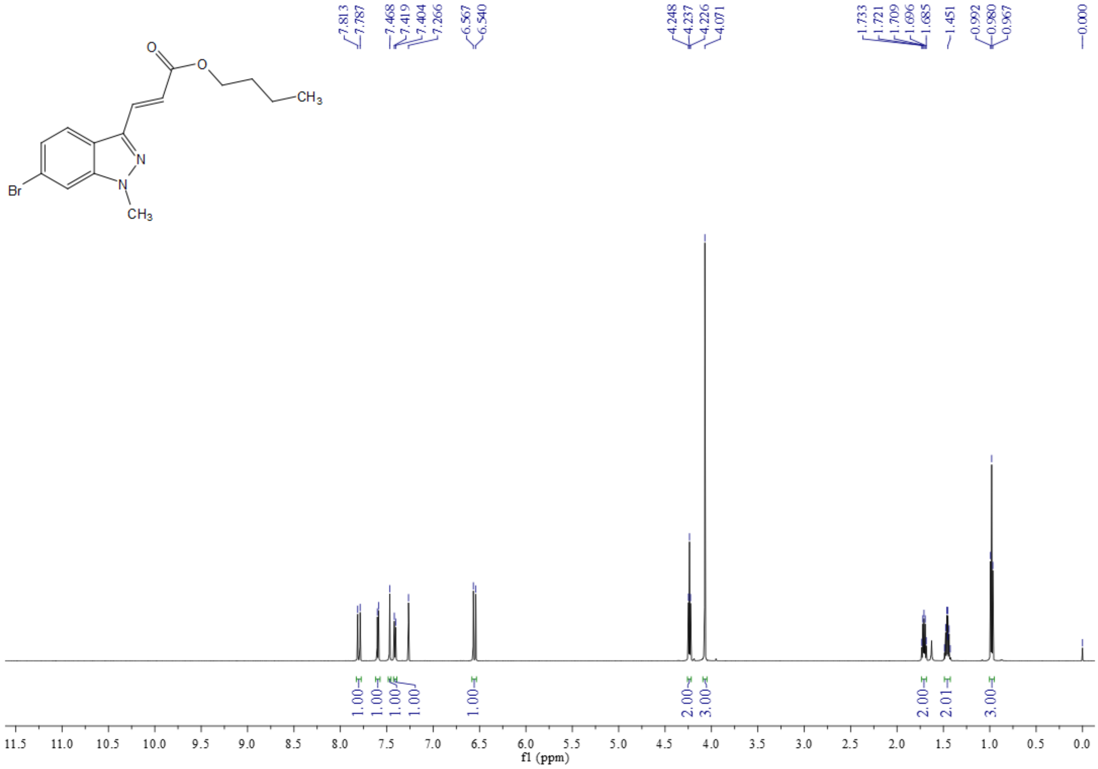

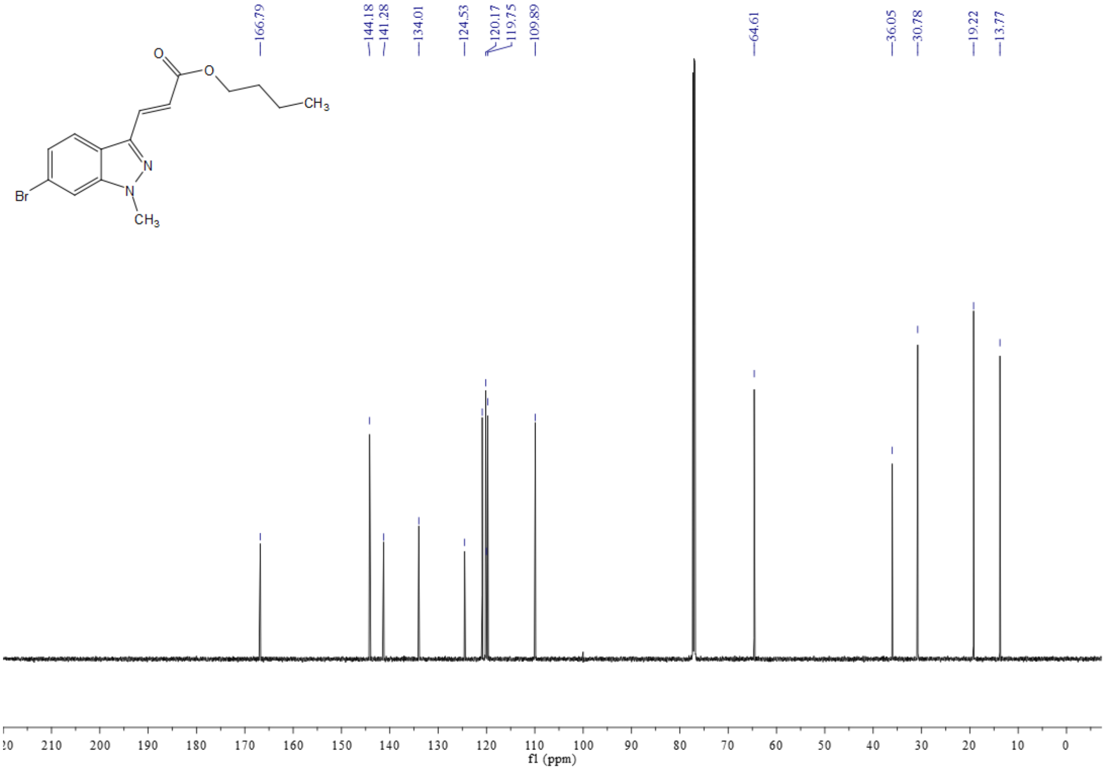

Butyl (*E*)-3-(6-chloro-1-methyl-1*H*-indazol-3-yl)acrylate (3ea).

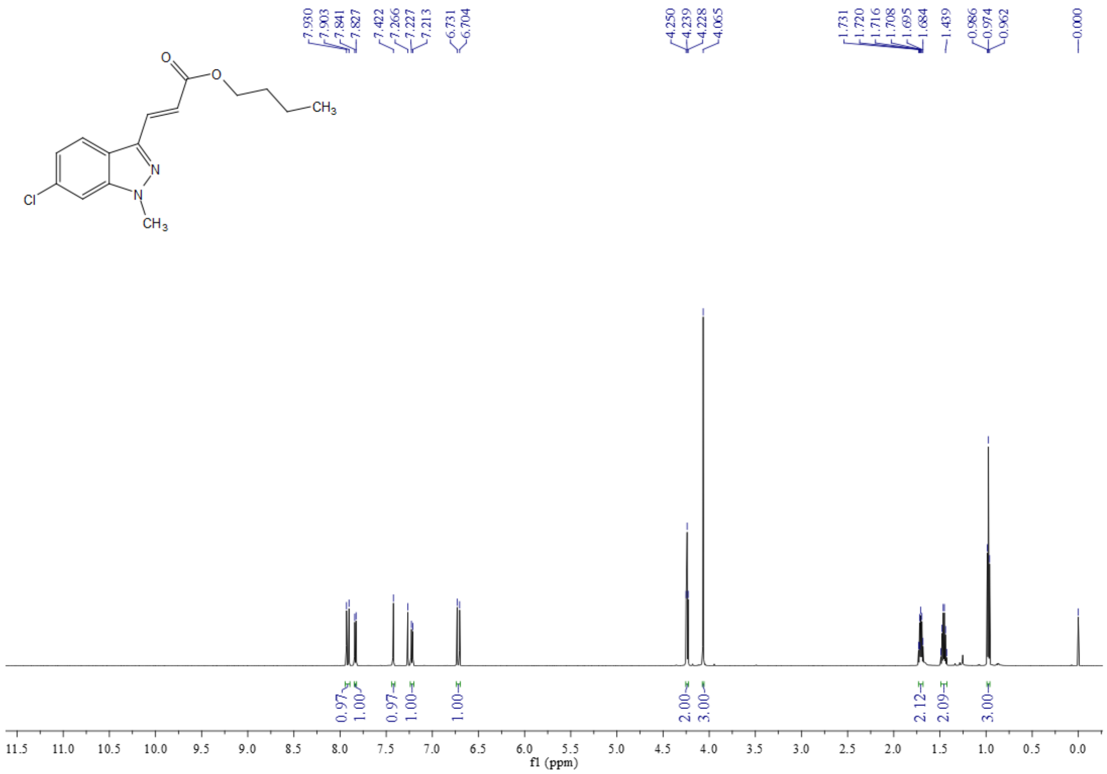

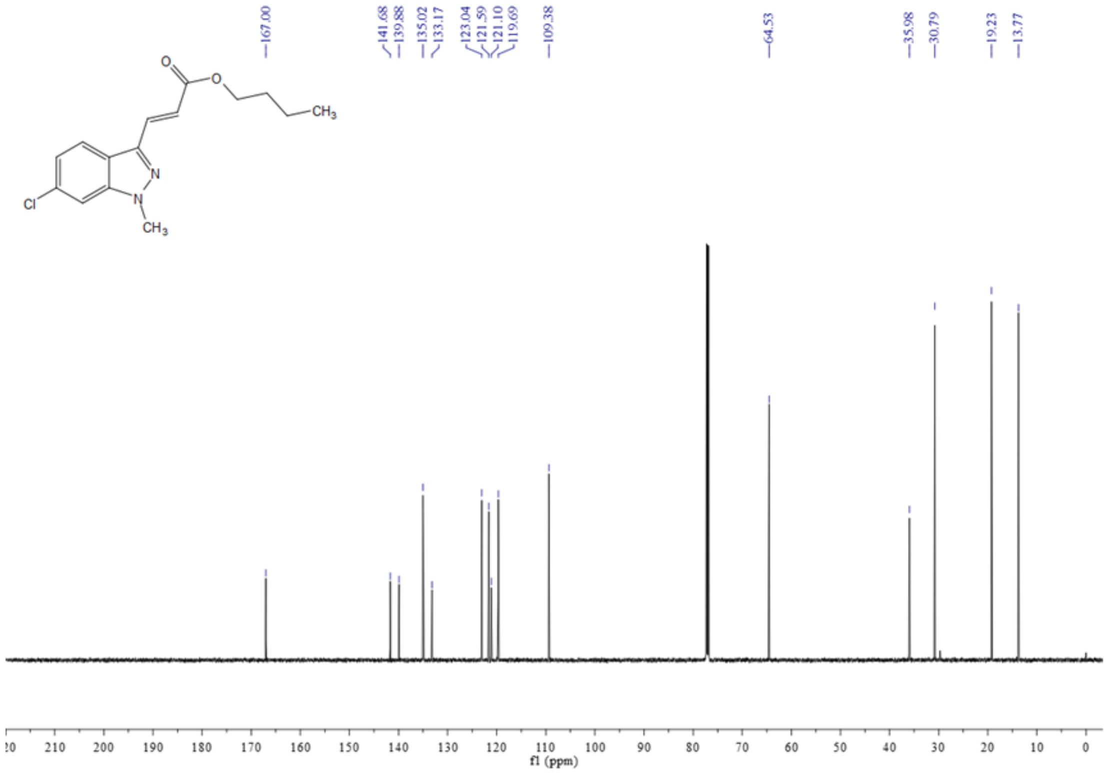

Butyl (*E*)-3-(6-cyano-1-methyl-1*H*-indazol-3-yl)acrylate (3fa).

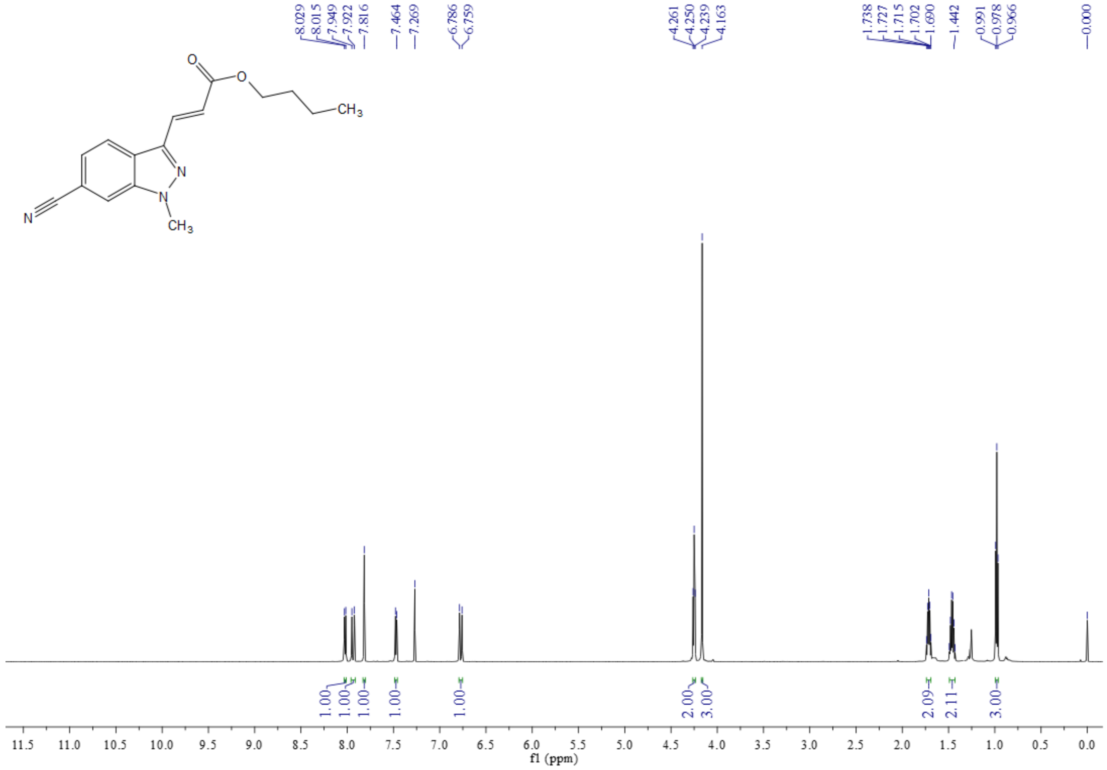

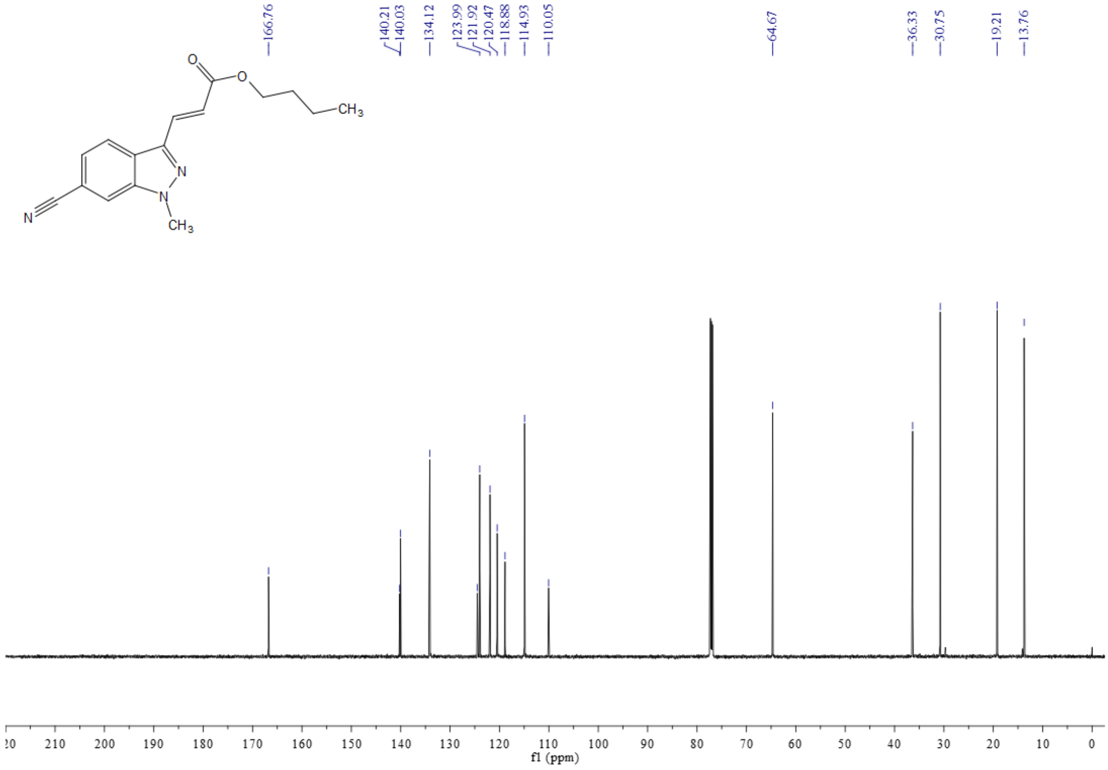

Butyl (*E*)-3-(1-methyl-6-nitro-1*H*-indazol-3-yl)acrylate (3ga).

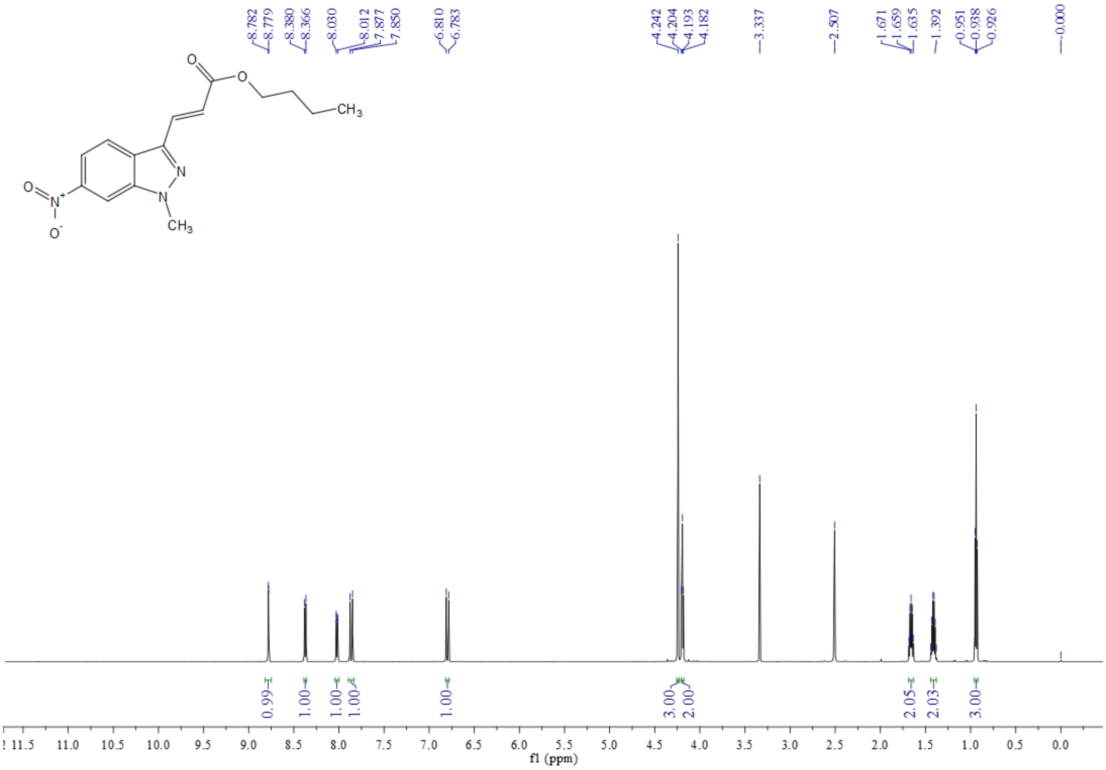

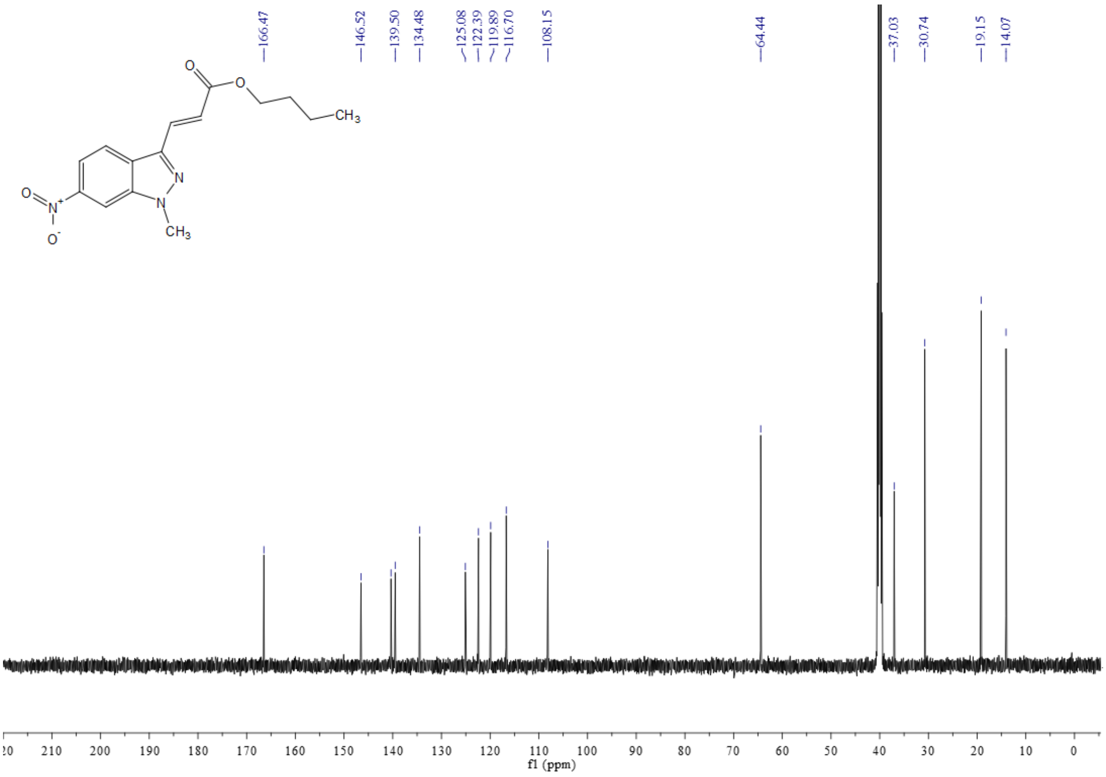

Butyl (*E*)-3-(7-chloro-1-methyl-1*H*-indazol-3-yl)acrylate (3ha).

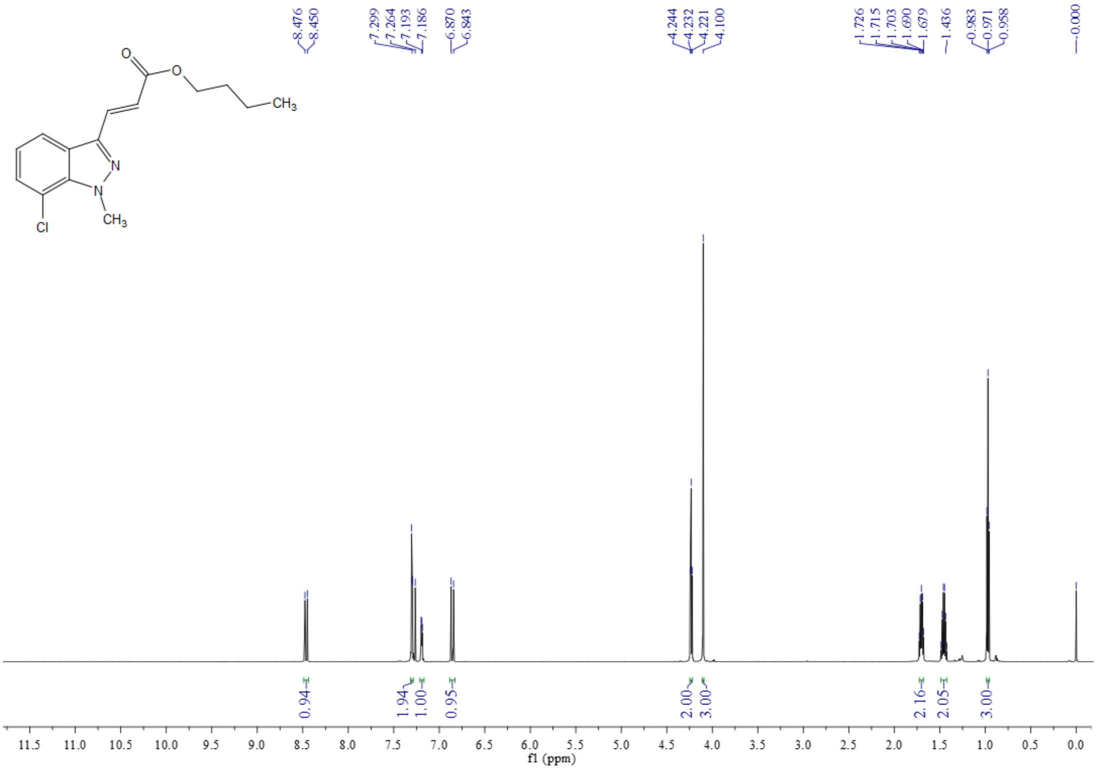

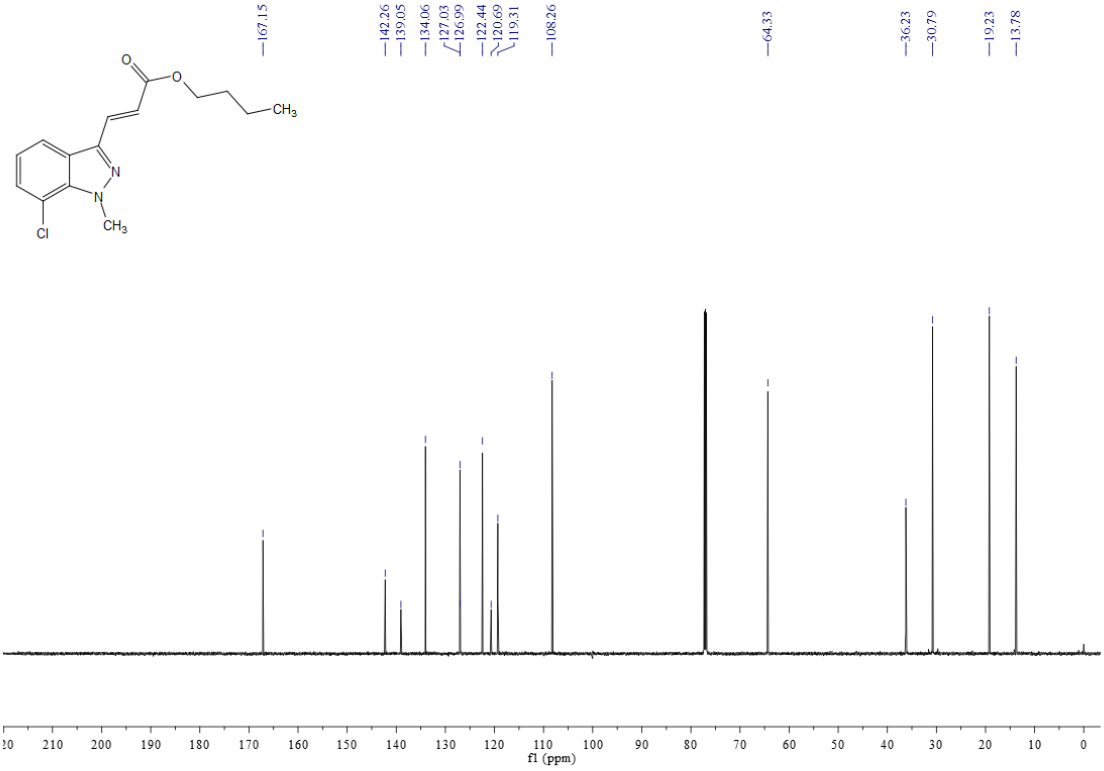

Butyl (*E*)-3-(5-methoxy-1-methyl-1*H*-indazol-3-yl)acrylate (3ia).

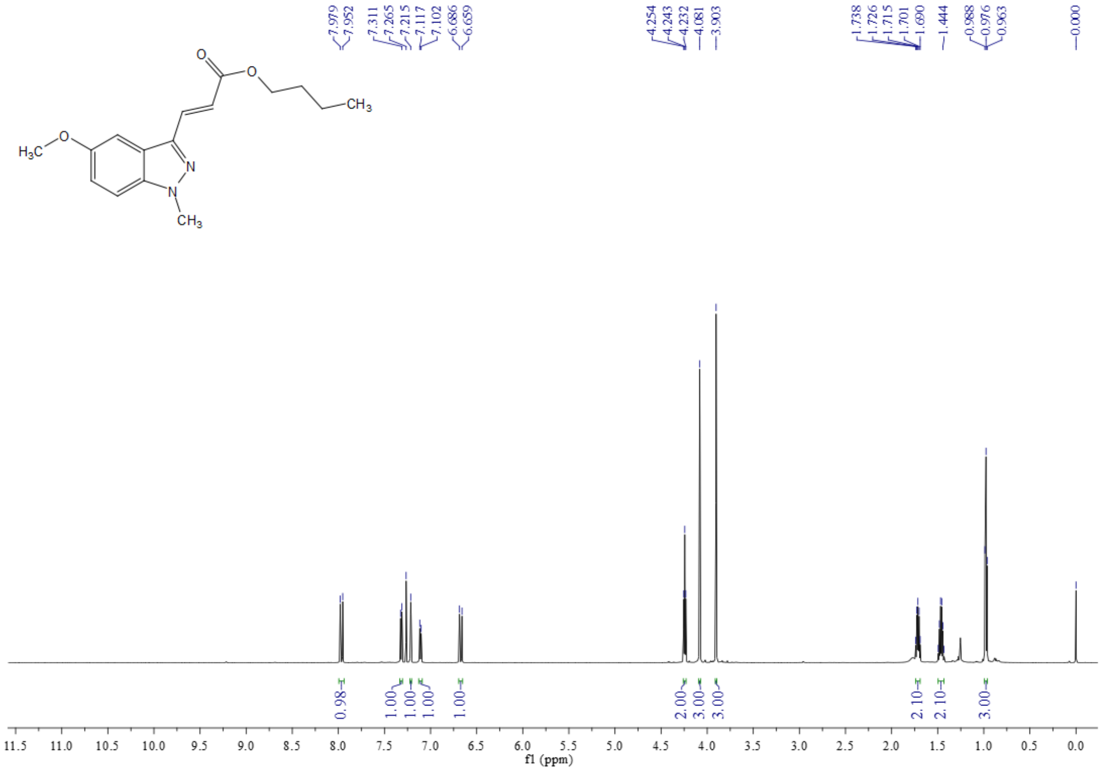

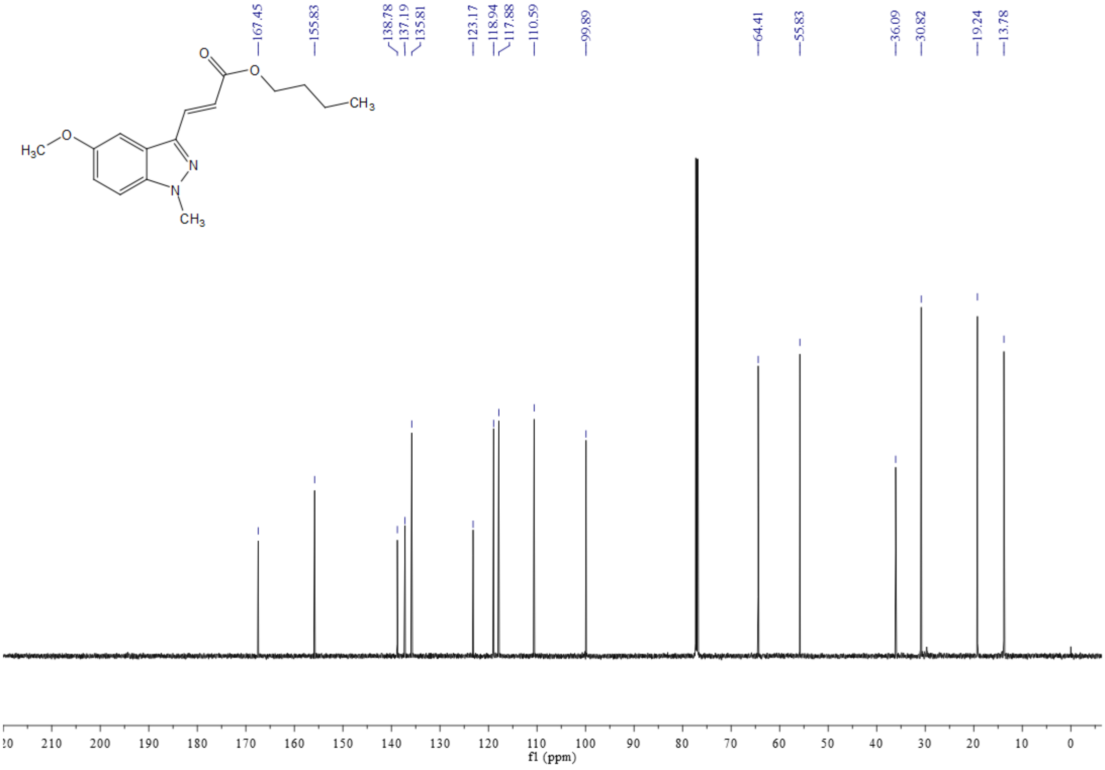

Butyl (*E*)-3-(1,6-dimethyl-1*H*-indazol-3-yl)acrylate (3ja).

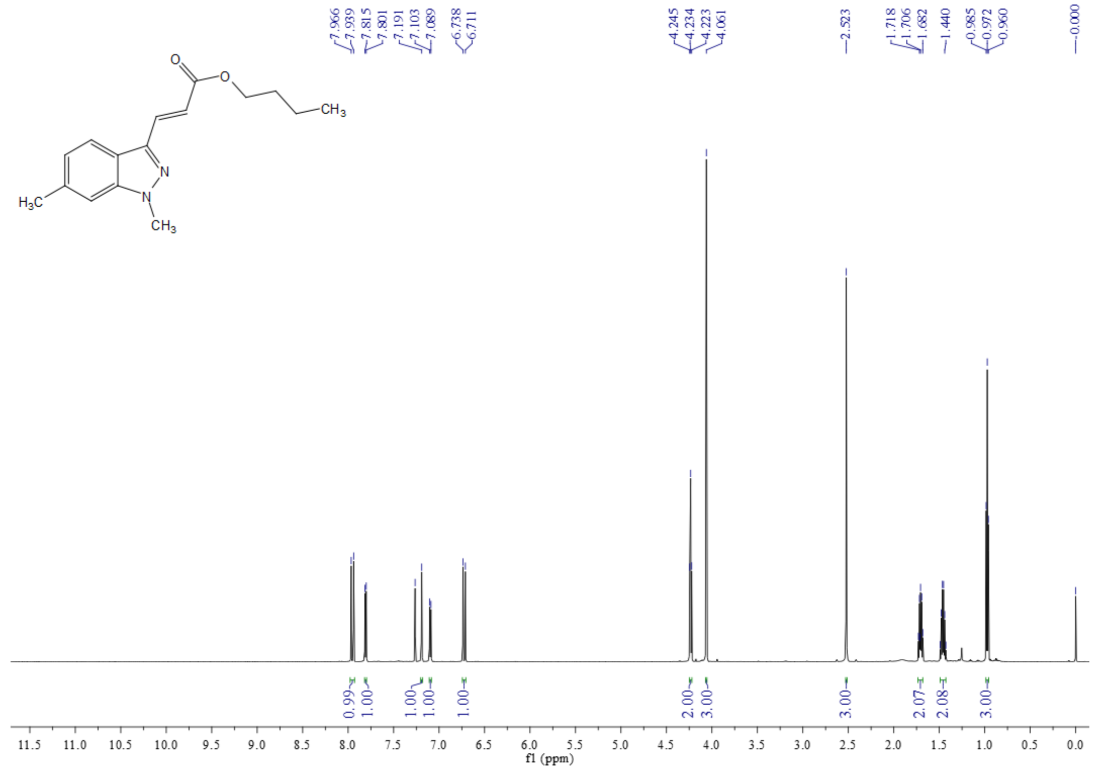

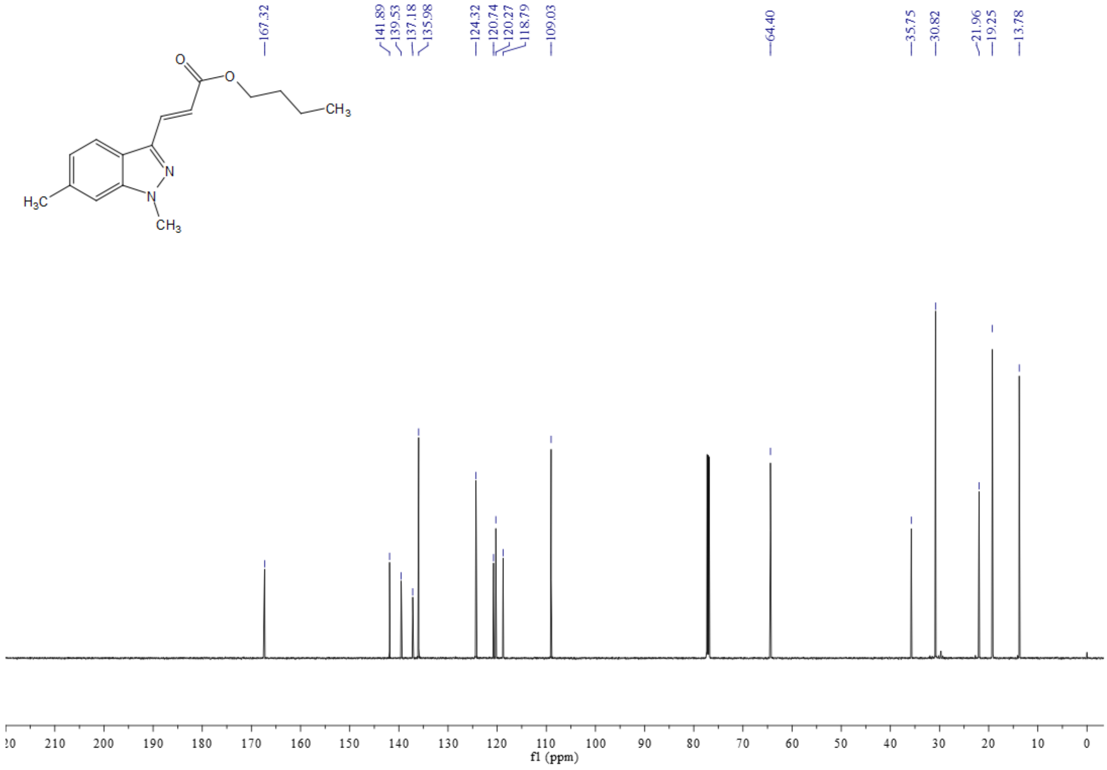

Butyl (*E*)-3-(6-acetamido-1-methyl-1*H*-indazol-3-yl)acrylate (3ka).

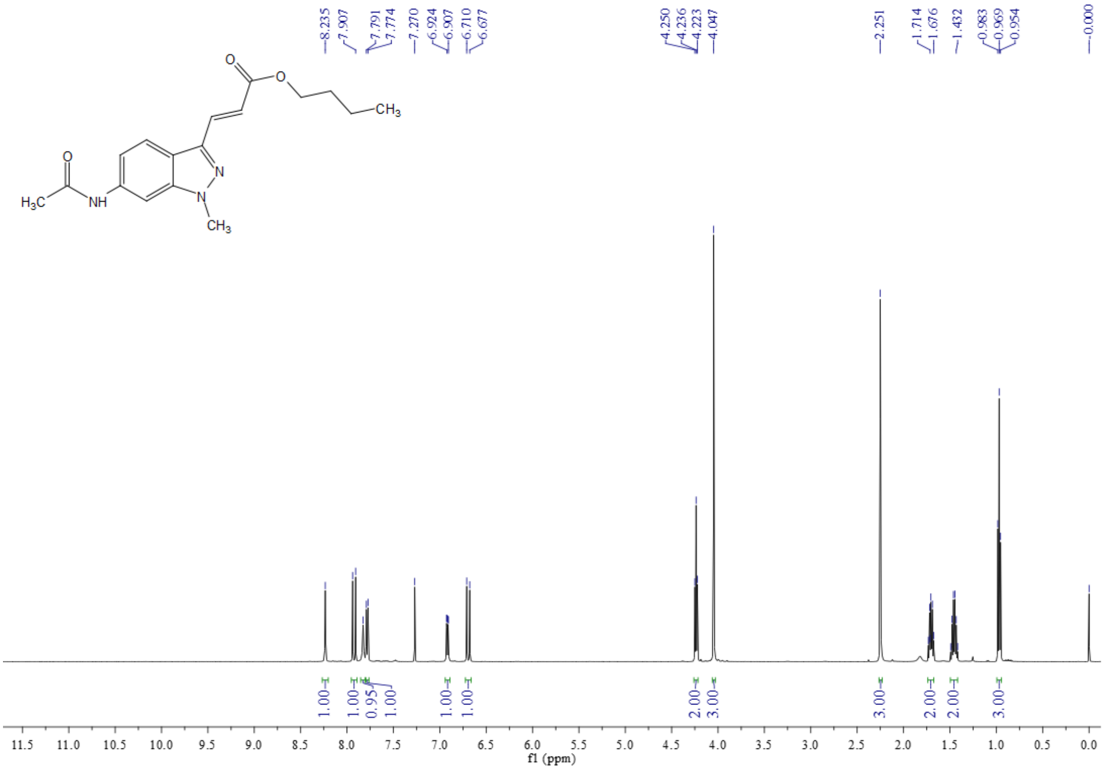

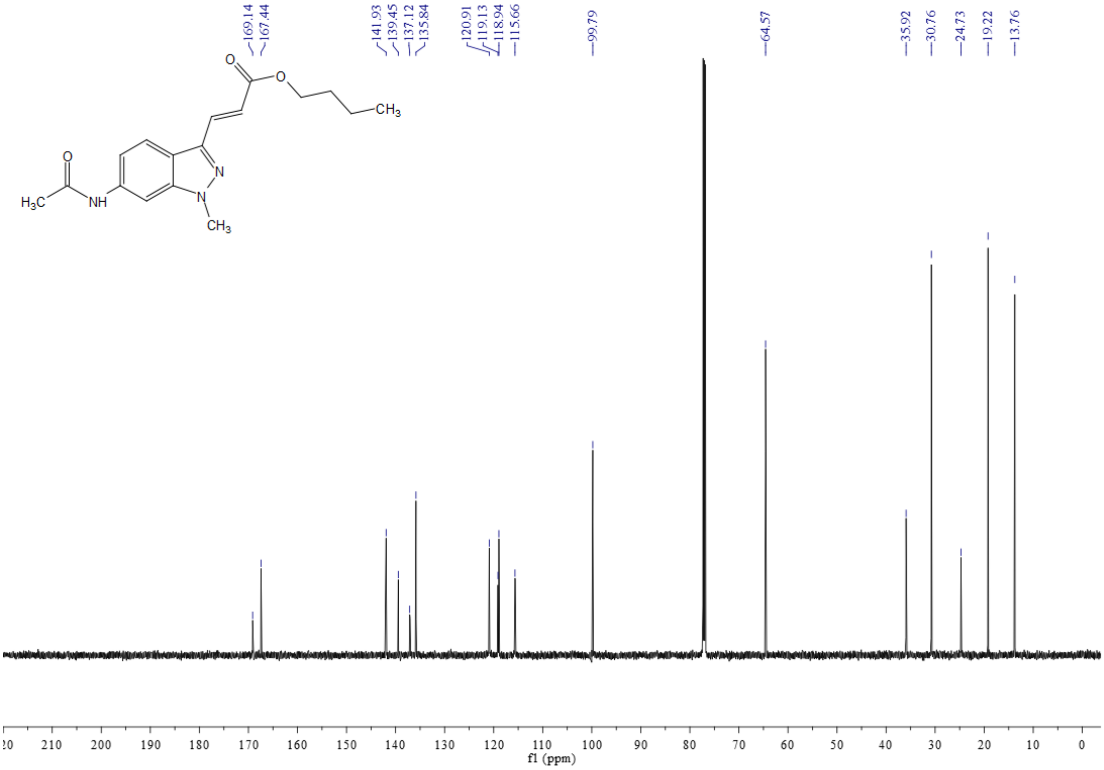

Butyl (*E*)-3-(1-(tetrahydro-2*H*-pyran-2-yl)-1*H*-indazol-3-yl)acrylate (3la).

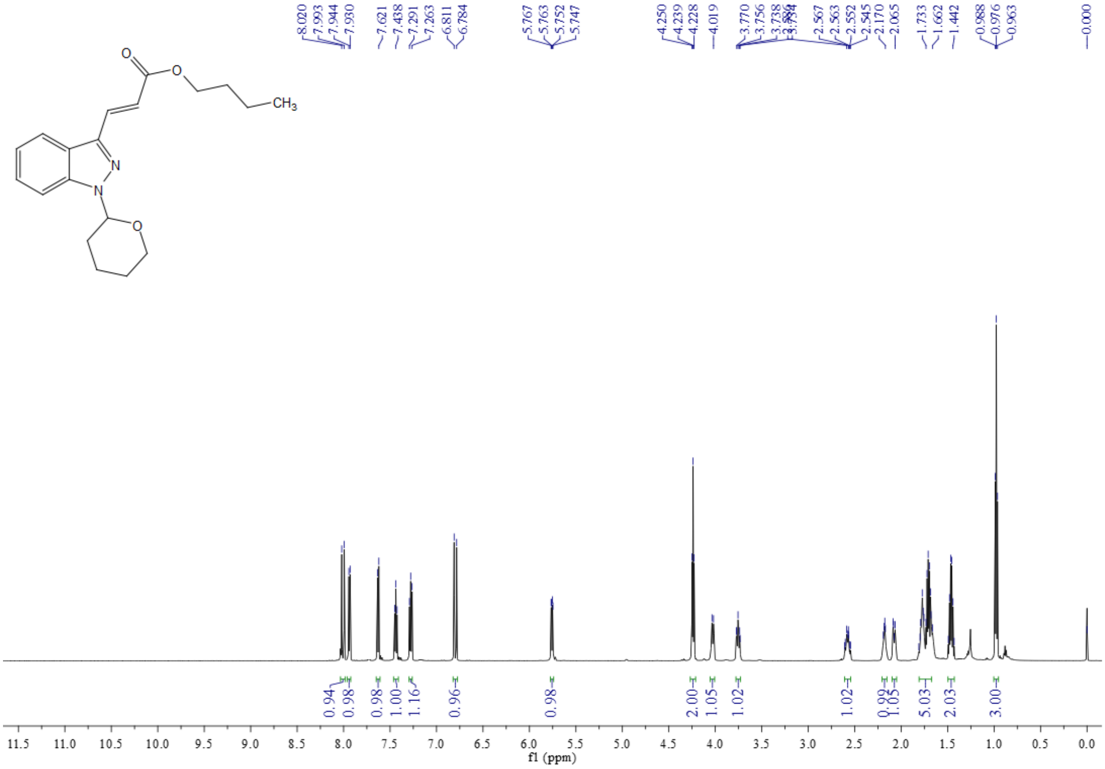

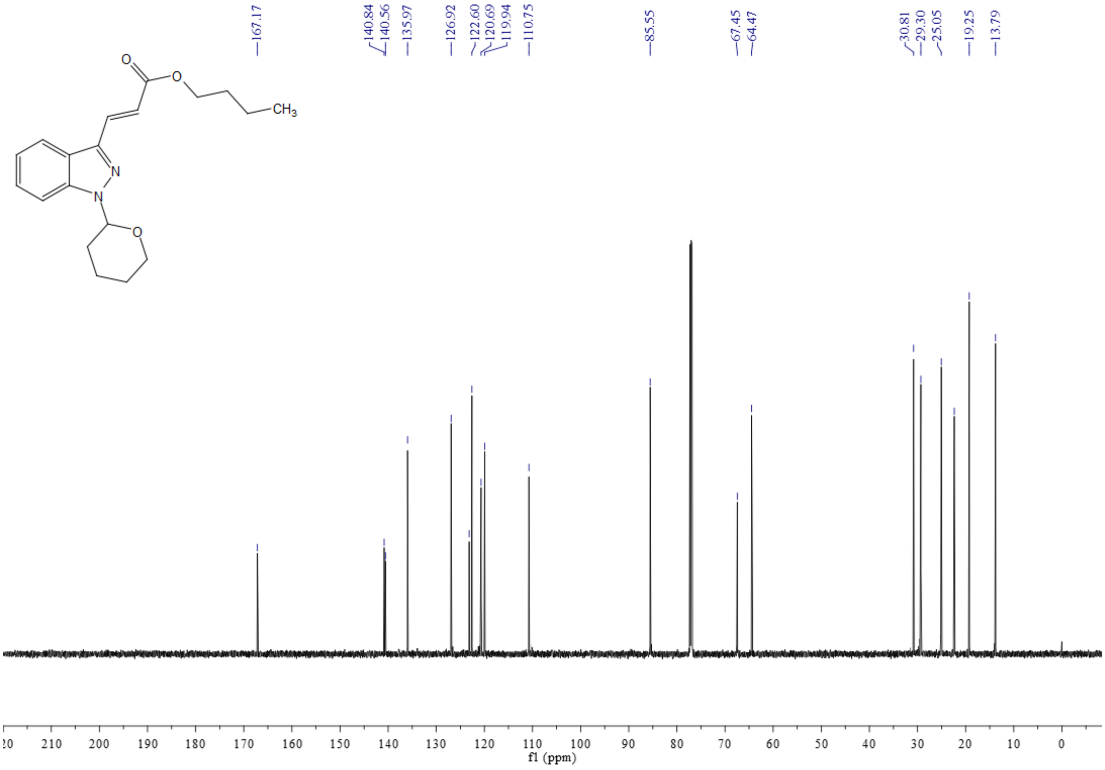

Butyl (*E*)-3-(1-benzyl-1*H*-indazol-3-yl)acrylate (3ma).

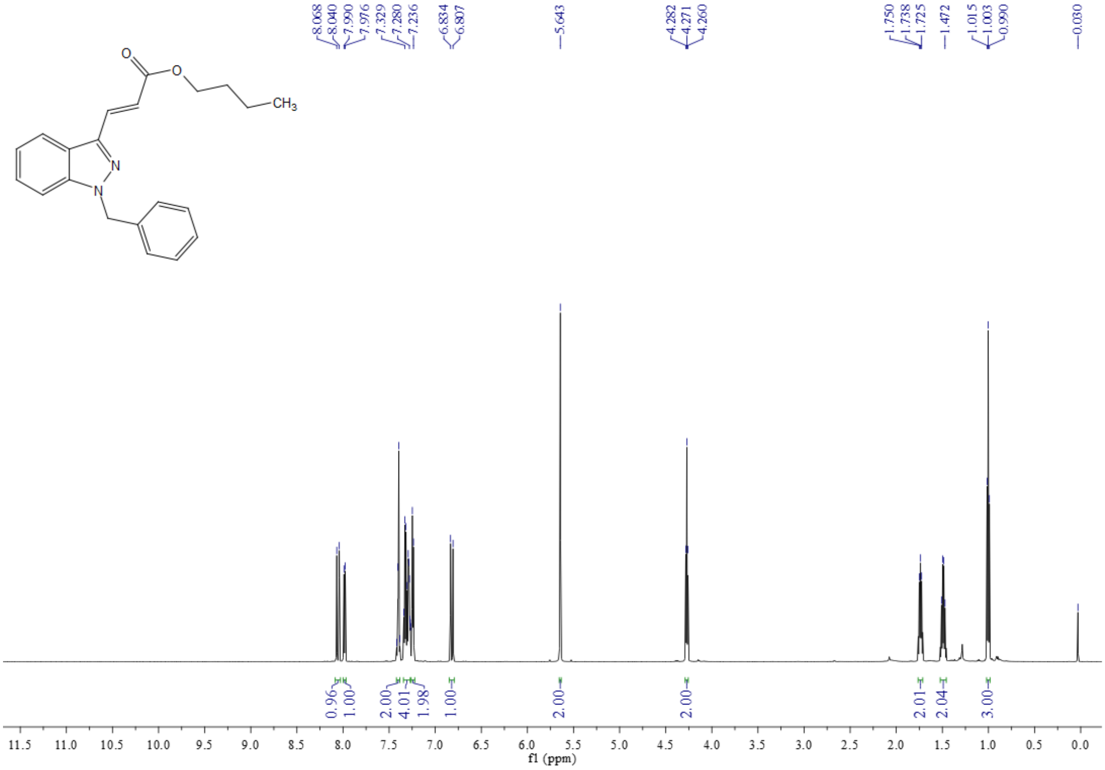

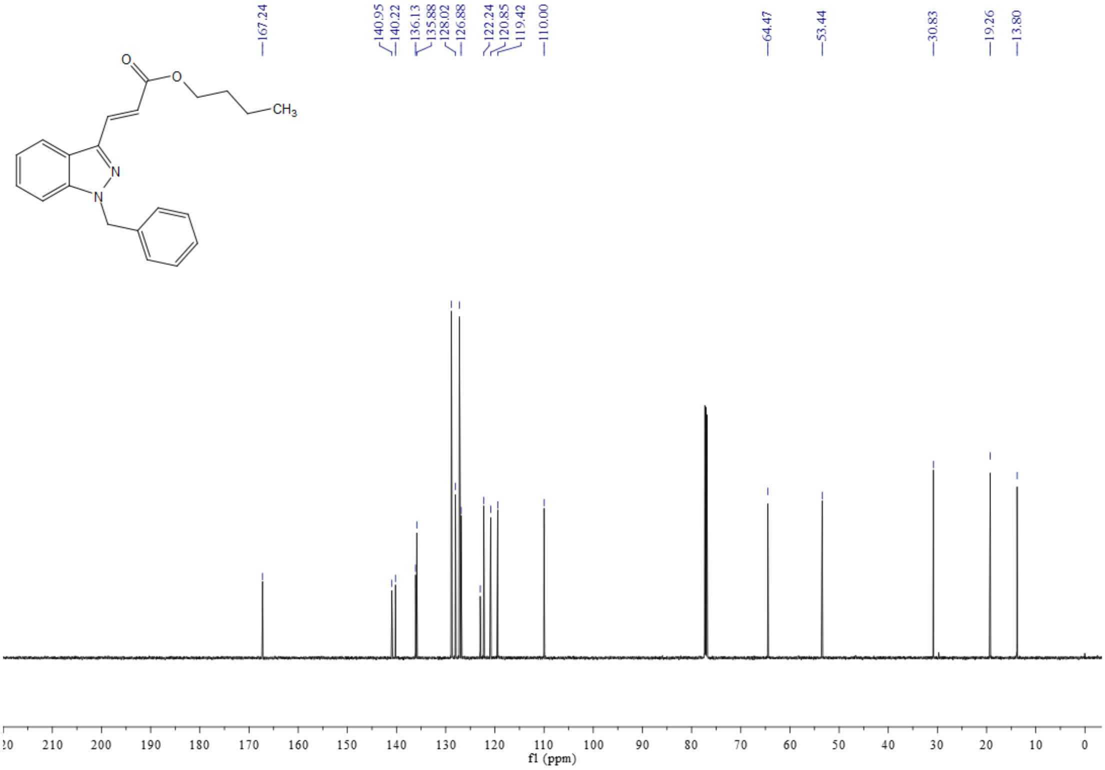

Butyl (*E*)-3-(1*H*-indazol-3-yl)acrylate (3na).

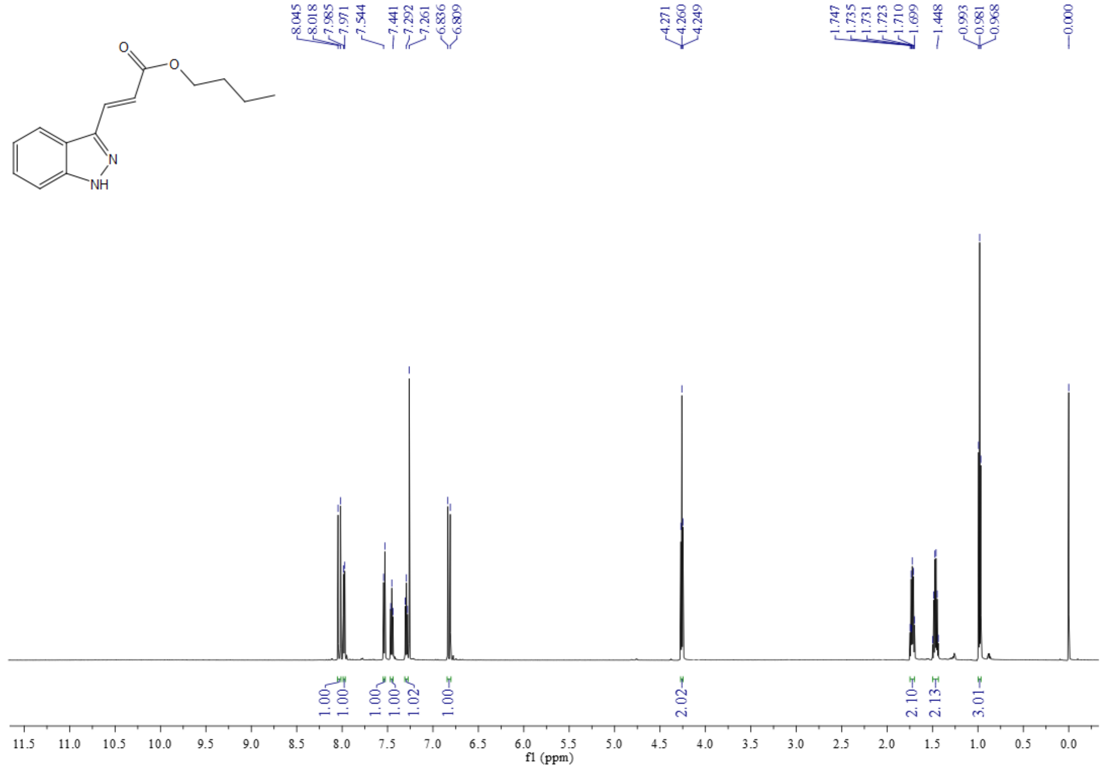

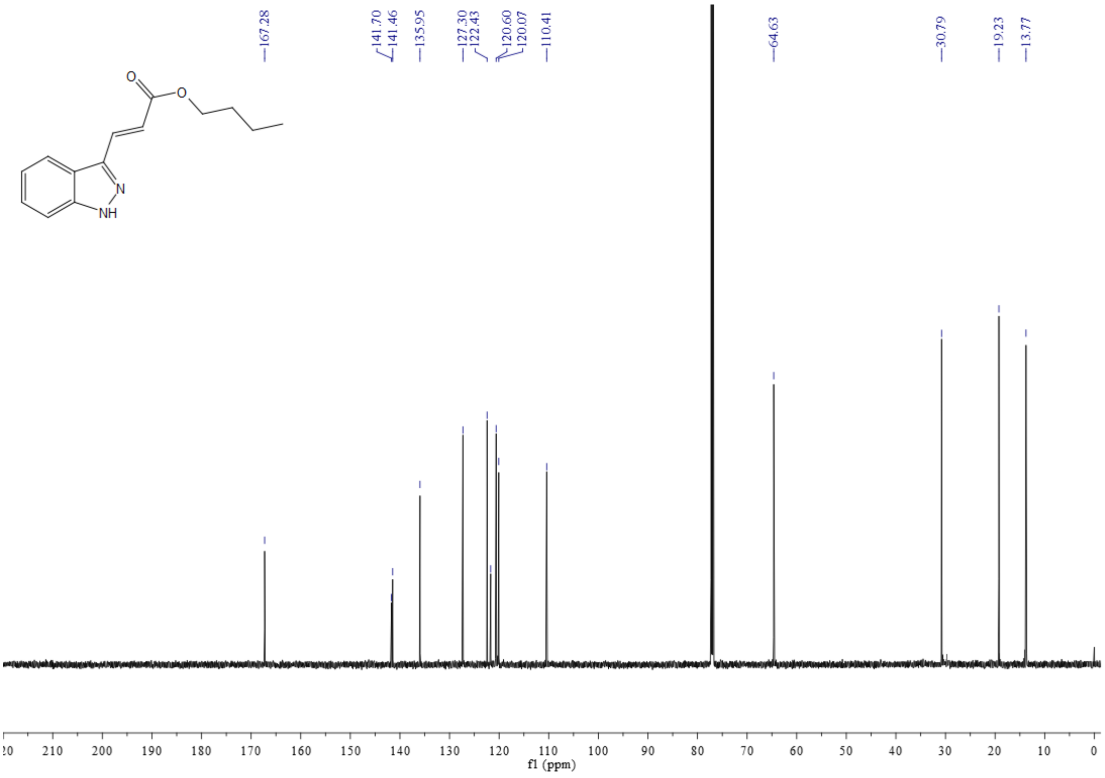

Butyl (*E*)-3-(1-(3-butoxy-3-oxopropyl)-1*H*-indazol-3-yl)acrylate (3oa).

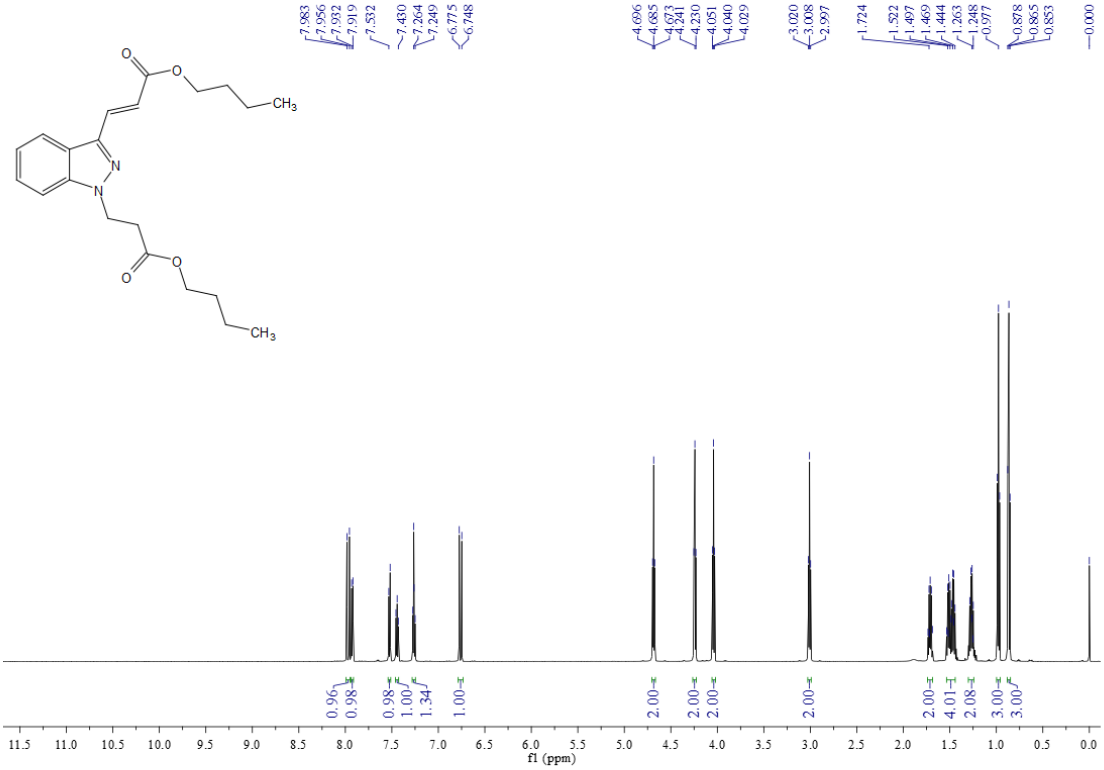

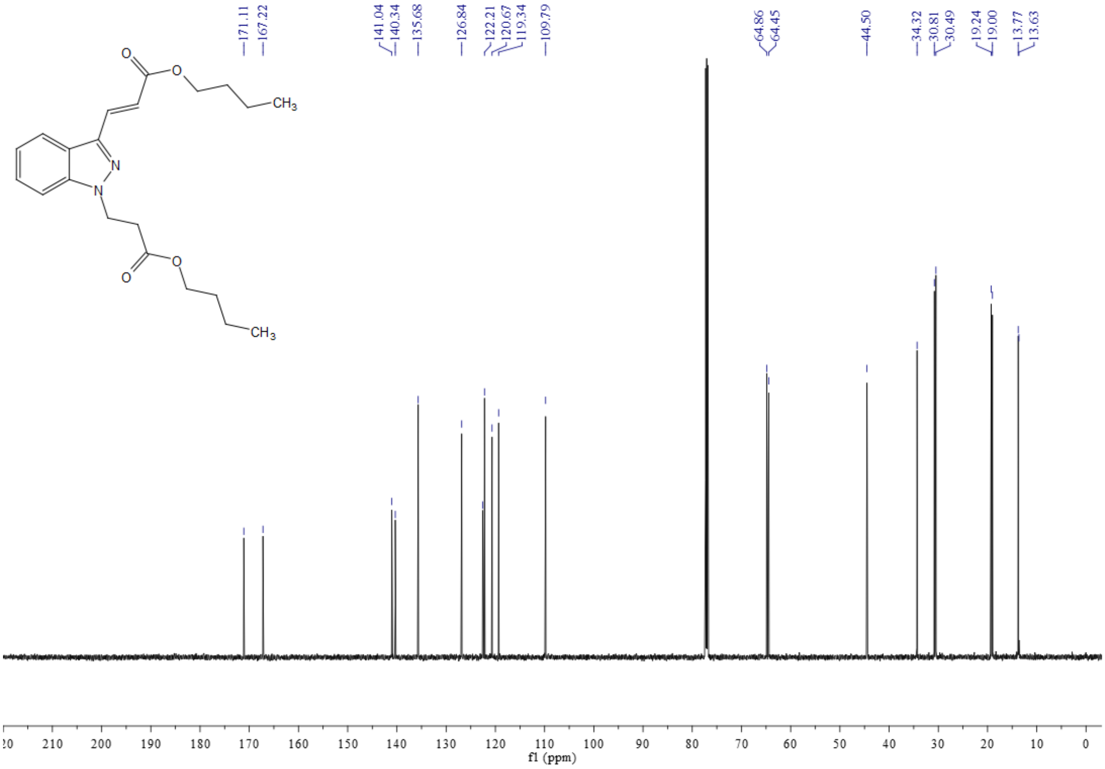

(*E*)-*N,N*-Diethyl-3-(1-methyl-1*H*-indazol-3-yl)acrylamide (3ab).

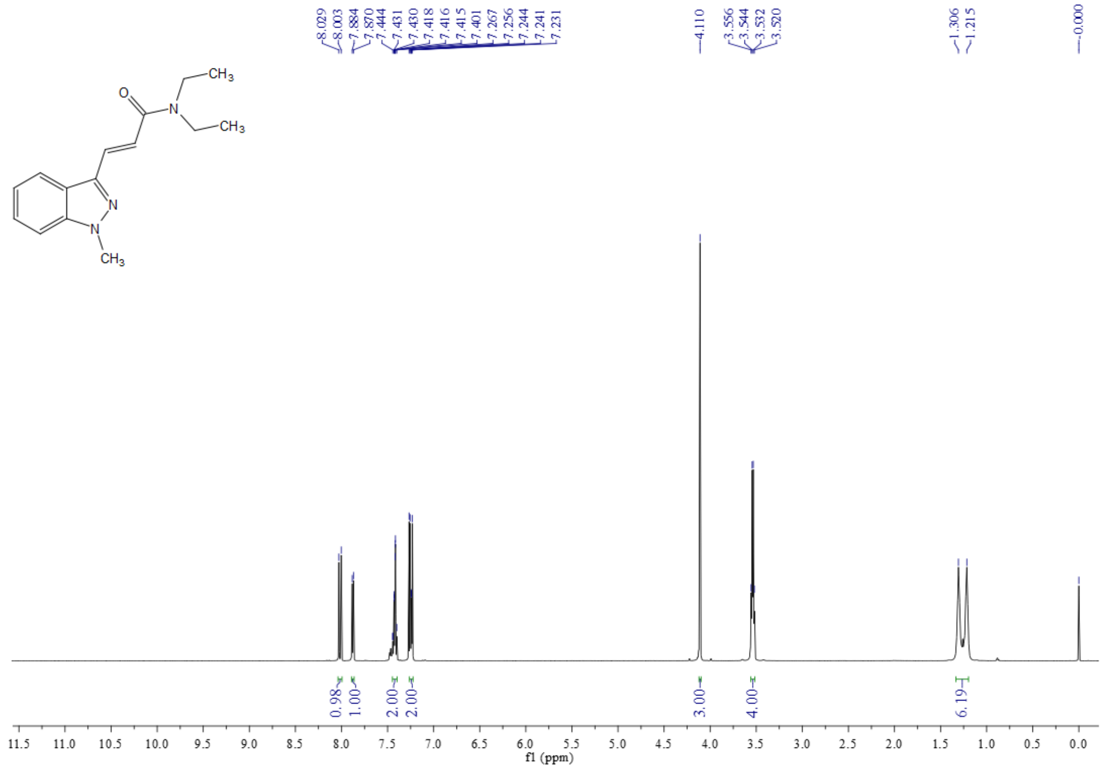

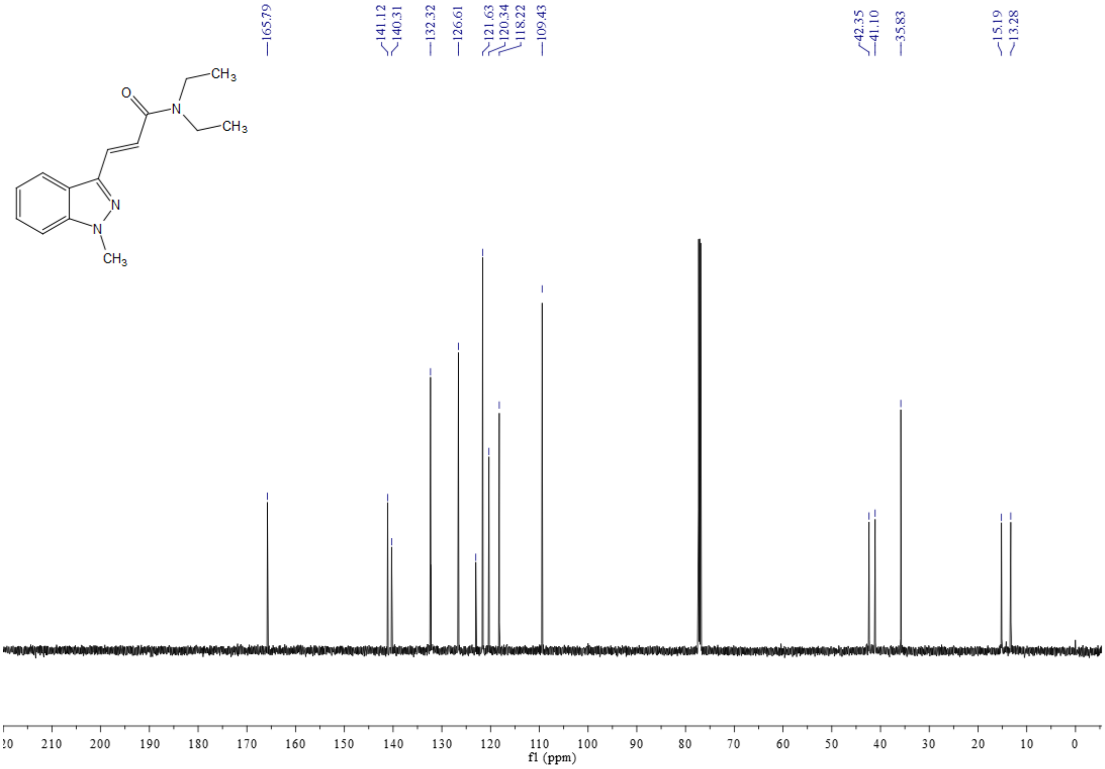

(*E*)-3-(1-Methyl-1*H*-indazol-3-yl)-1-(piperidin-1-yl)prop-2-en-1-one (3ac).

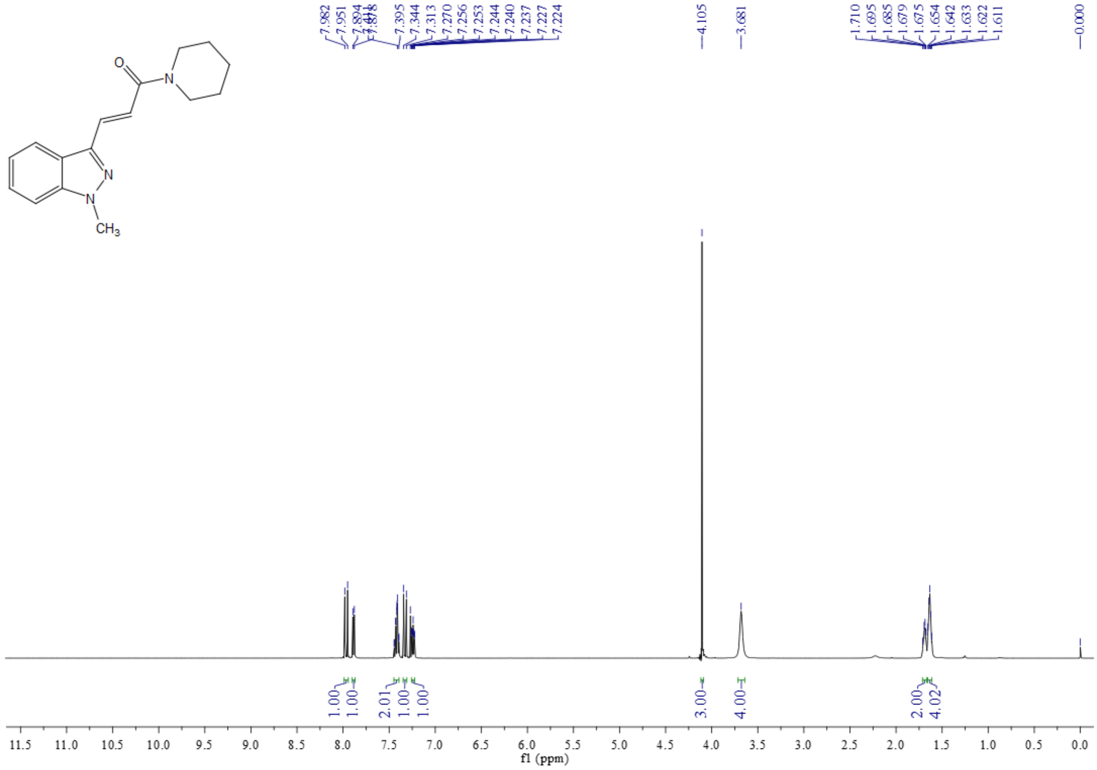

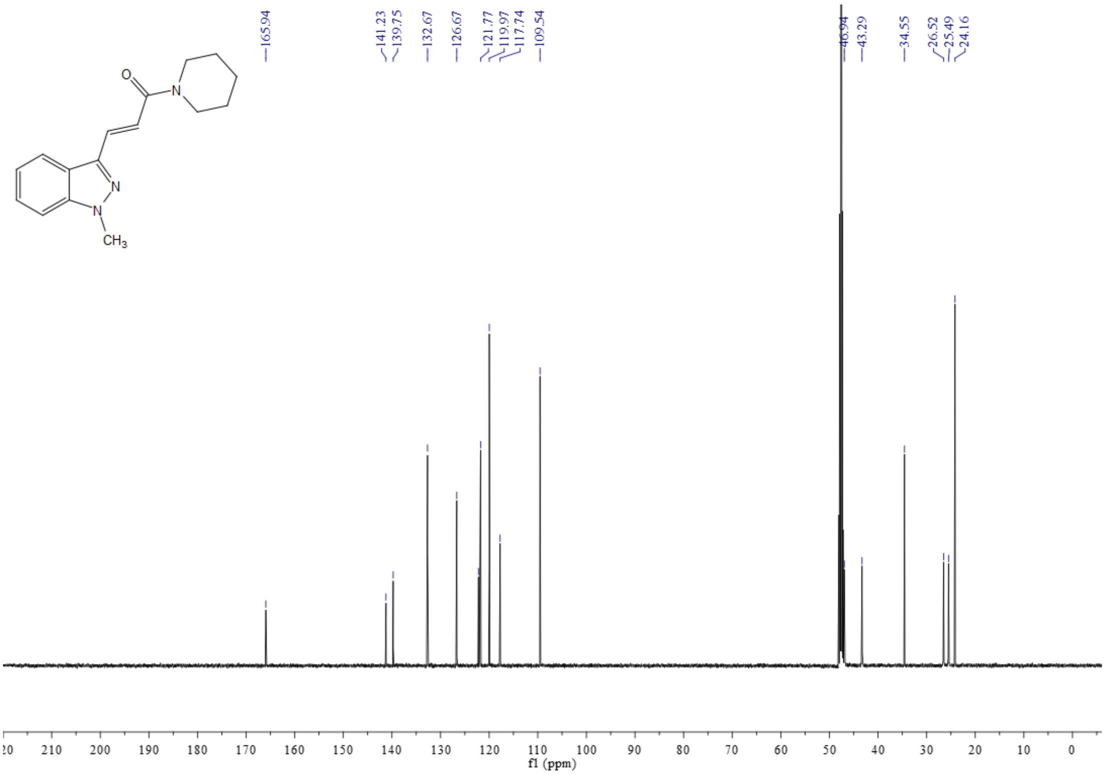

Butyl 2-((1-methyl-1*H*-indazol-3-yl)methyl)acrylate (3ad).

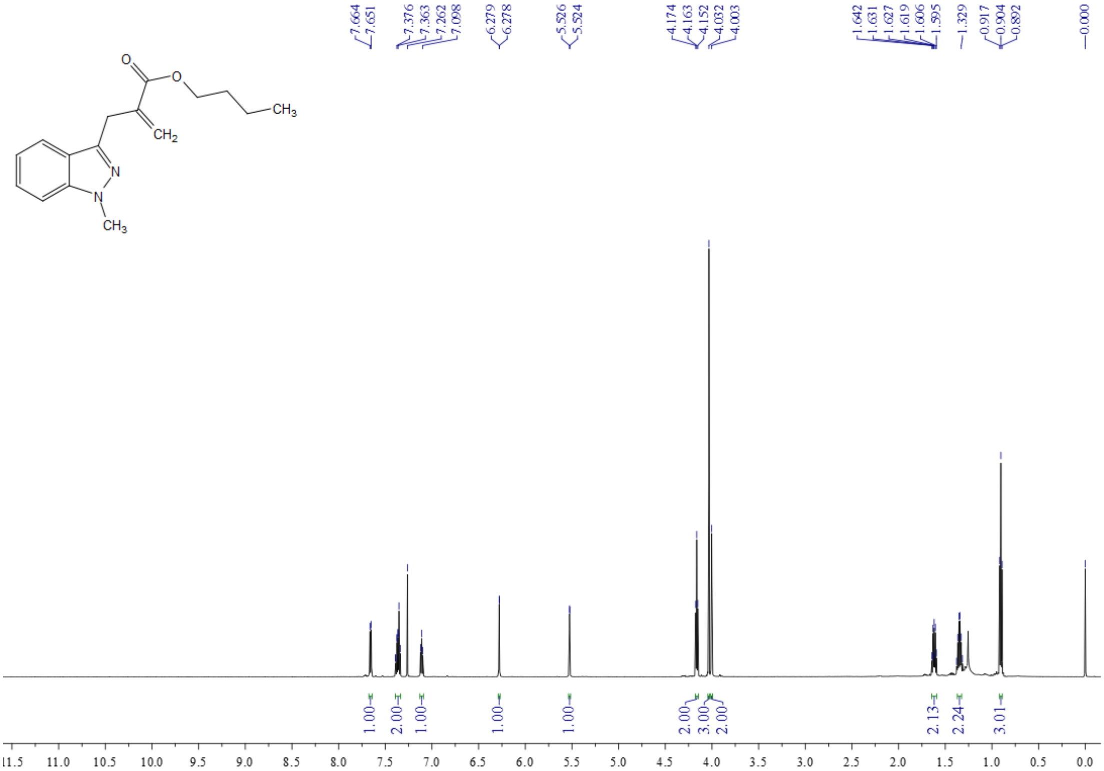

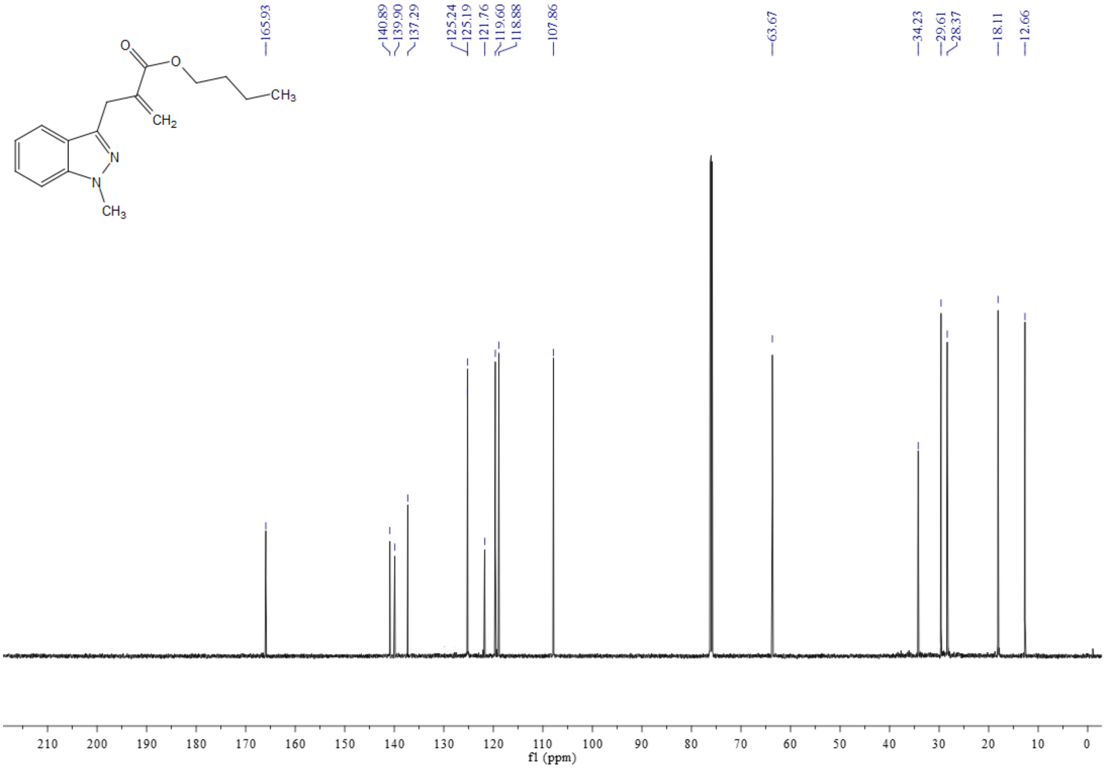

Methyl 2-((1-methyl-1*H*-indazol-3-yl)methyl)acrylate (3ae).

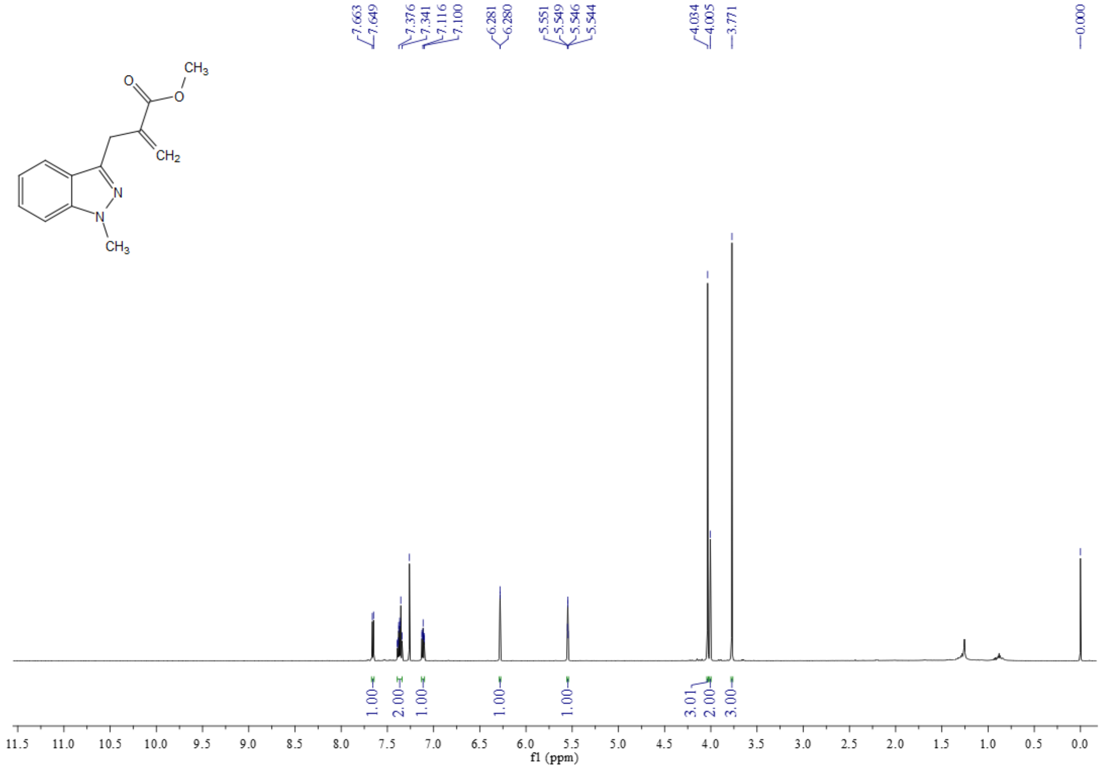

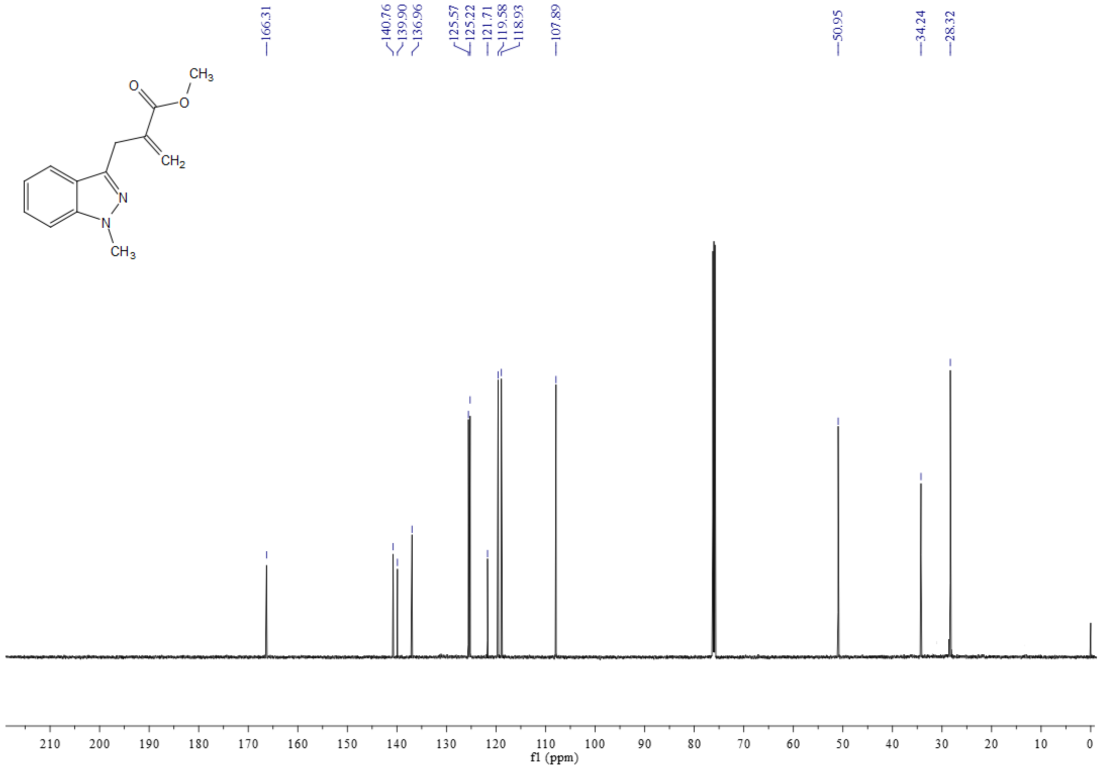

(*E*)-1-Methyl-3-styryl-1*H*-indazole (3af).

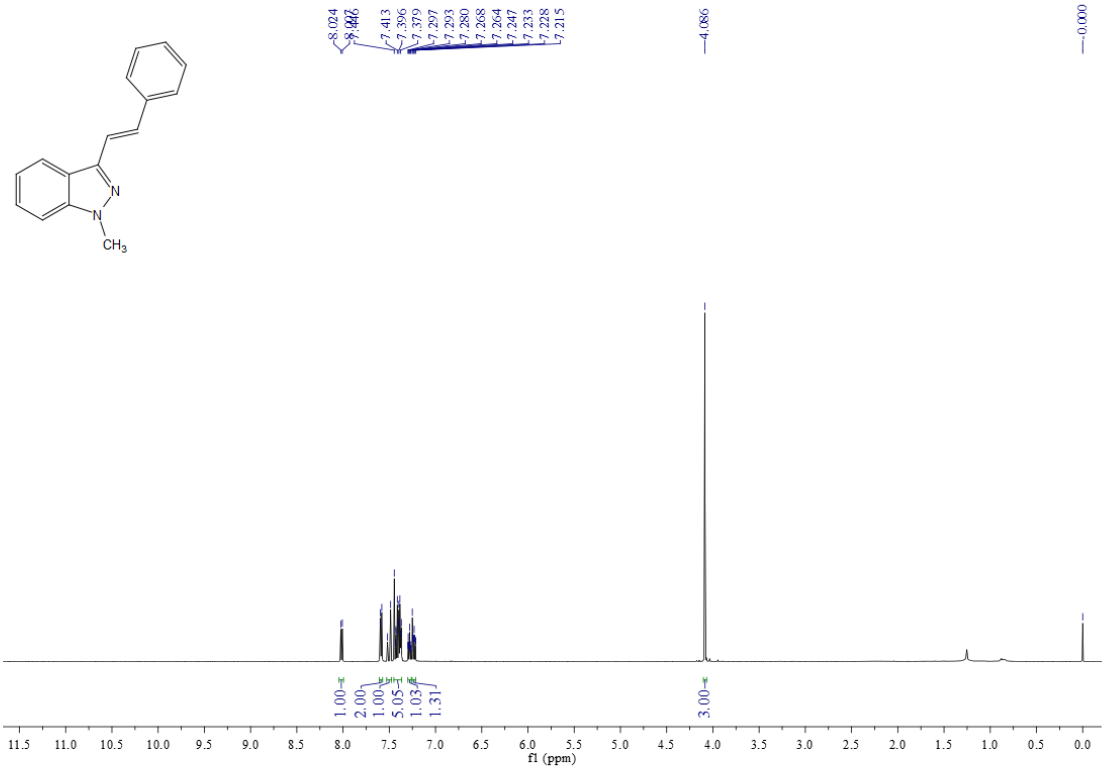

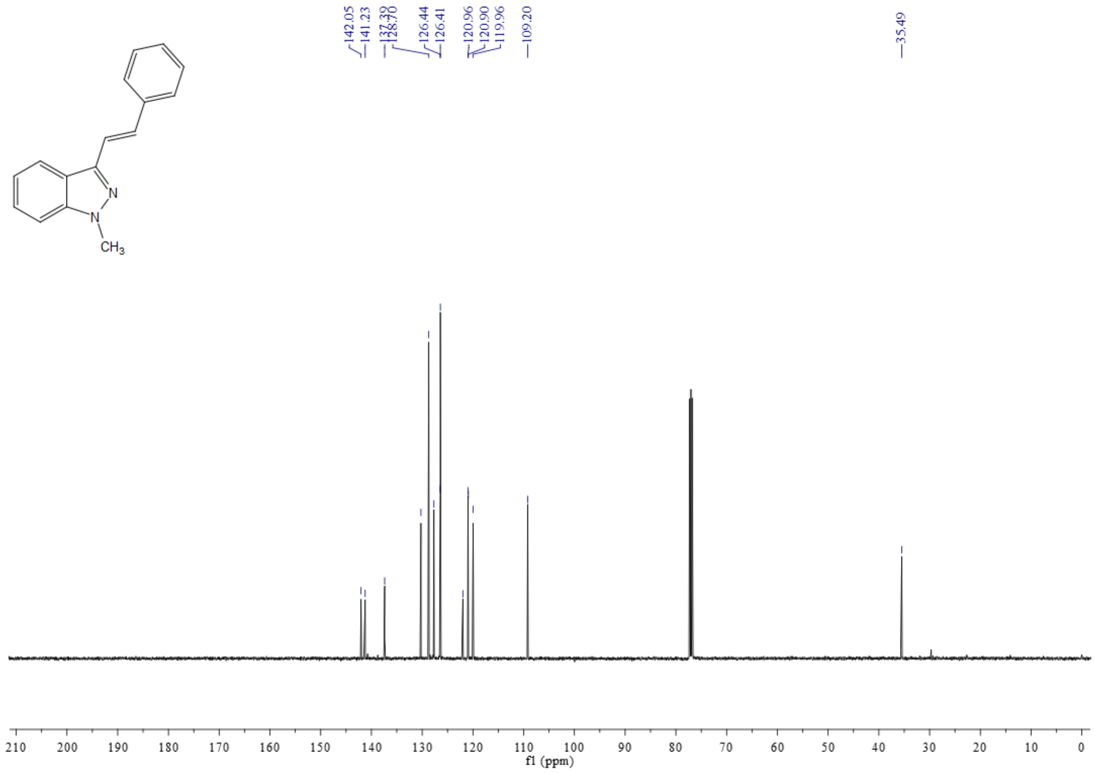

(*E*)-1-Methyl-3-(4-methylstyryl)-1*H*-indazole (3ag).

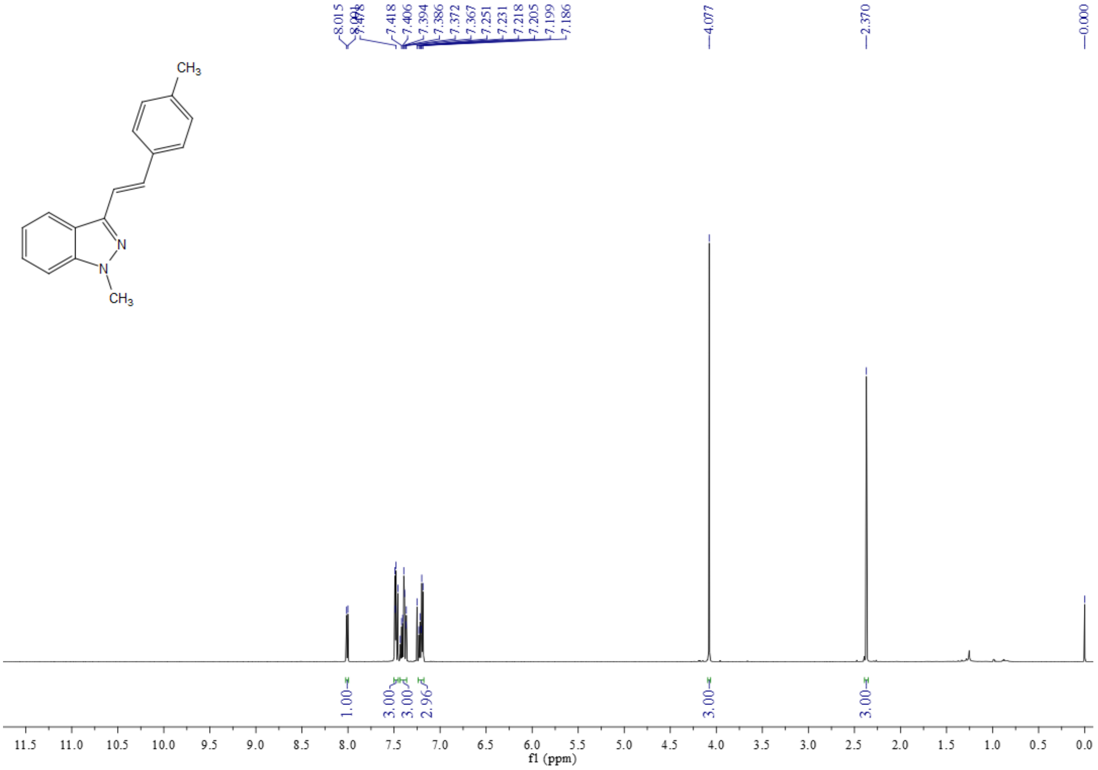

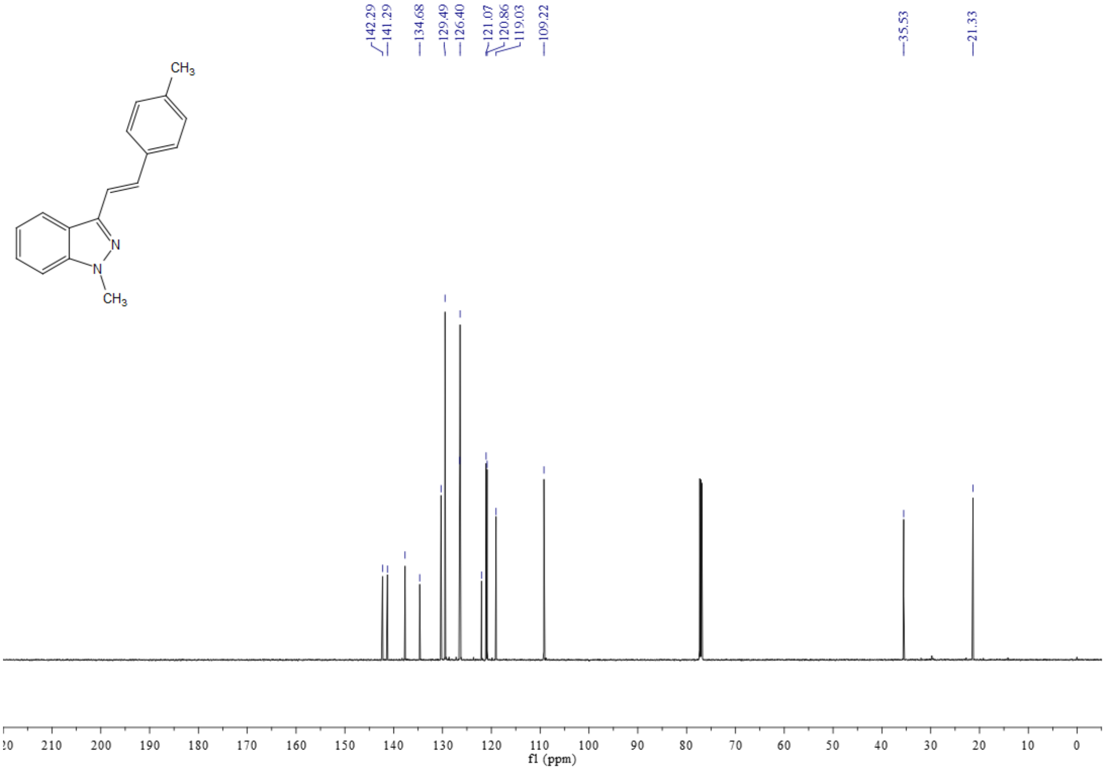

(*E*)-3-(4-Methoxystyryl)-1-methyl-1*H*-indazole (3ah).

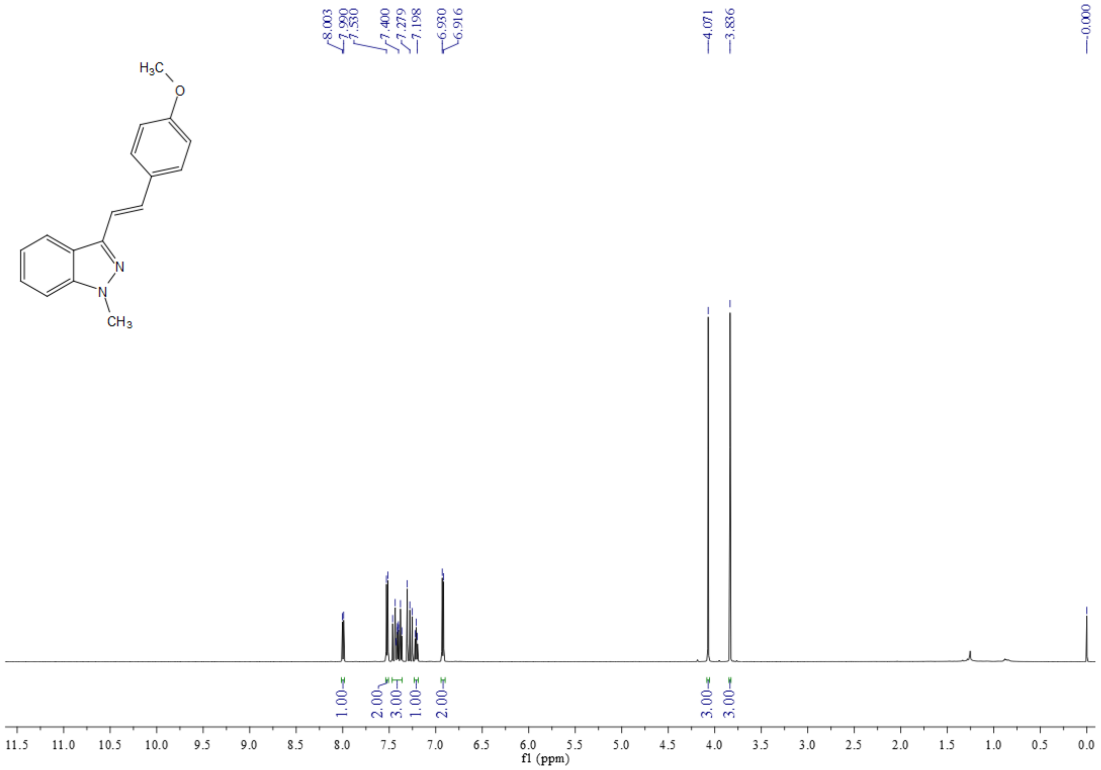

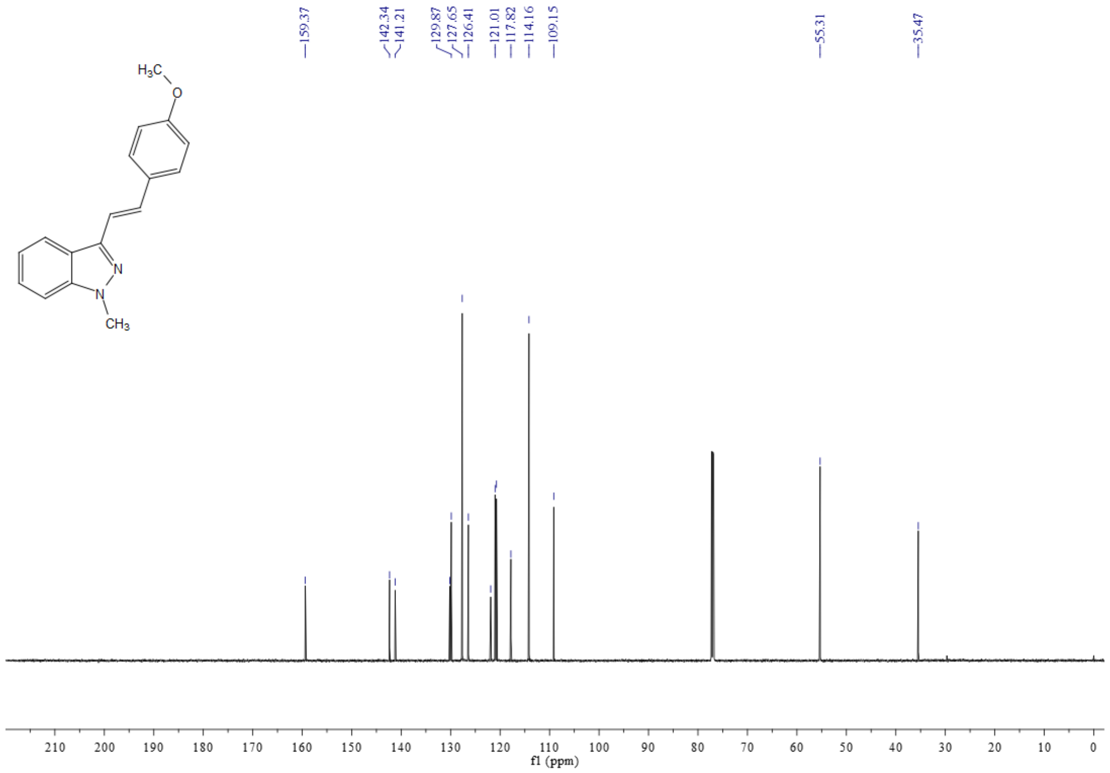

(*E*)-4-(2-(1-Methyl-1*H*-indazol-3-yl)vinyl)phenol (3ai).

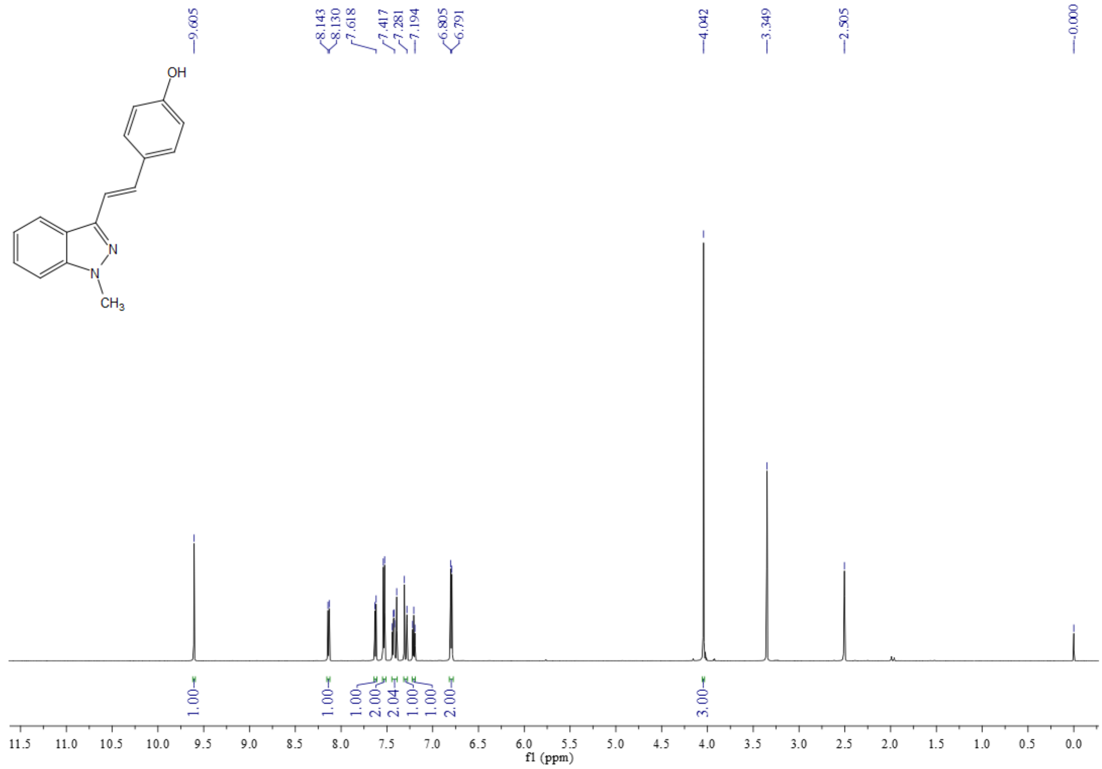

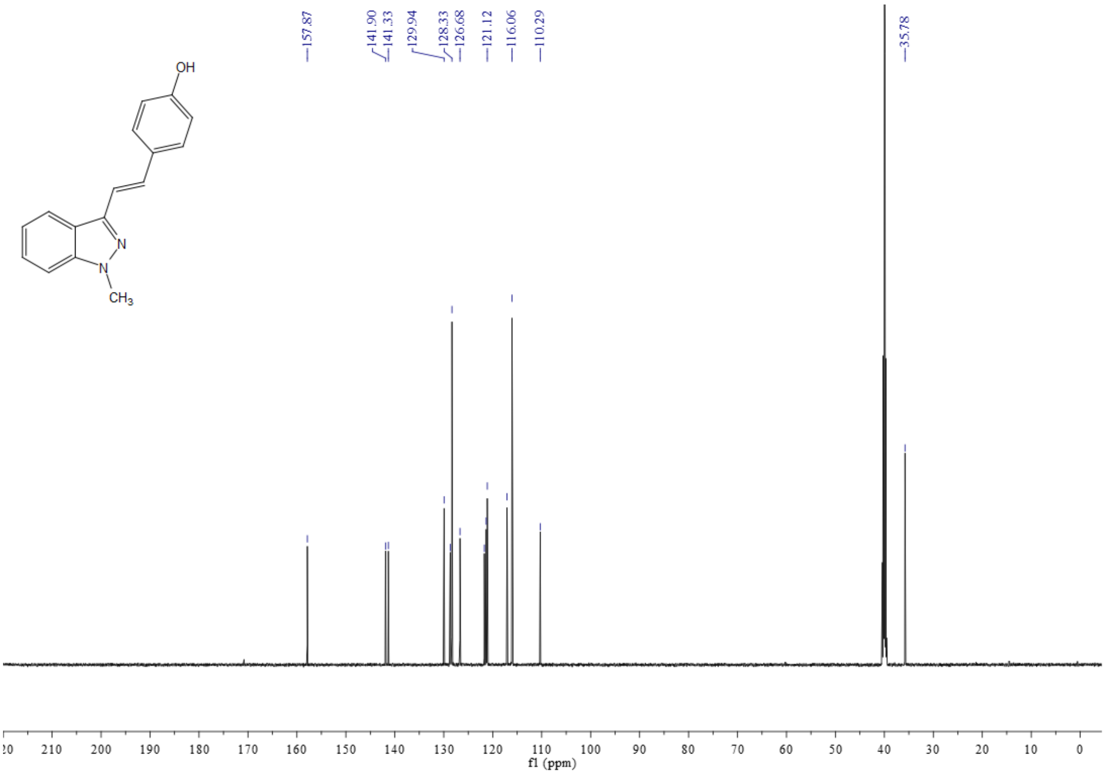

(*E*)-4-(2-(1-Methyl-1*H*-indazol-3-yl)vinyl)benzonitrile (3aj).

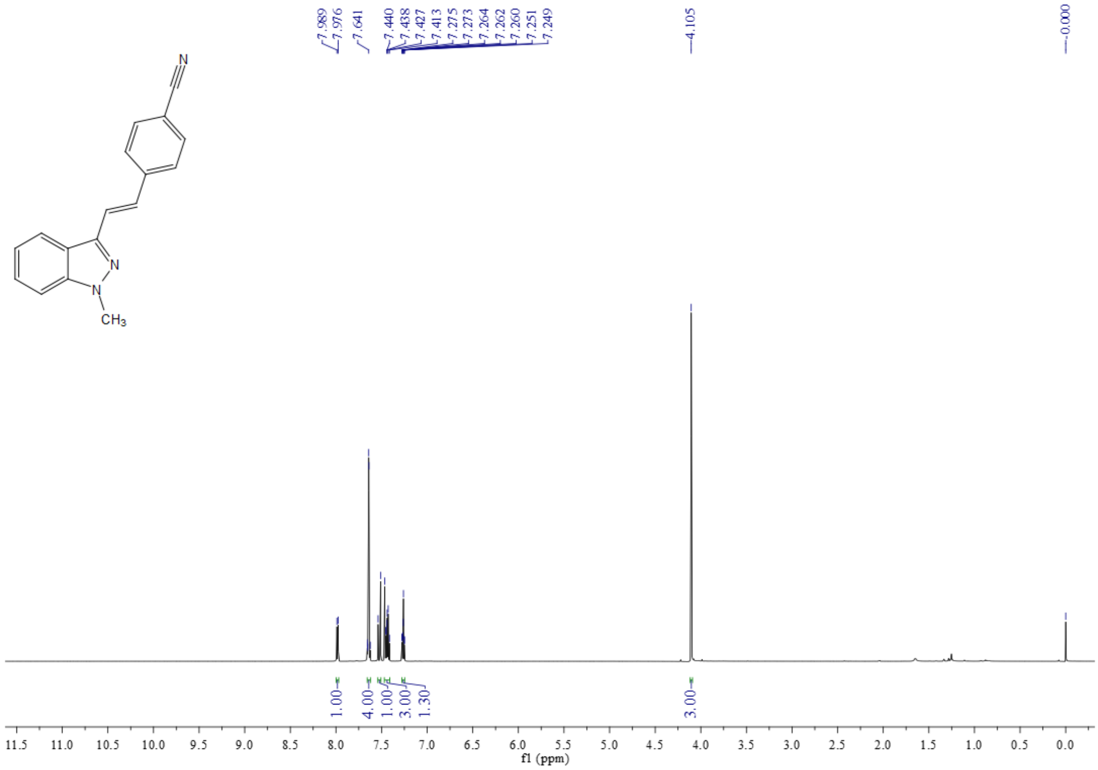

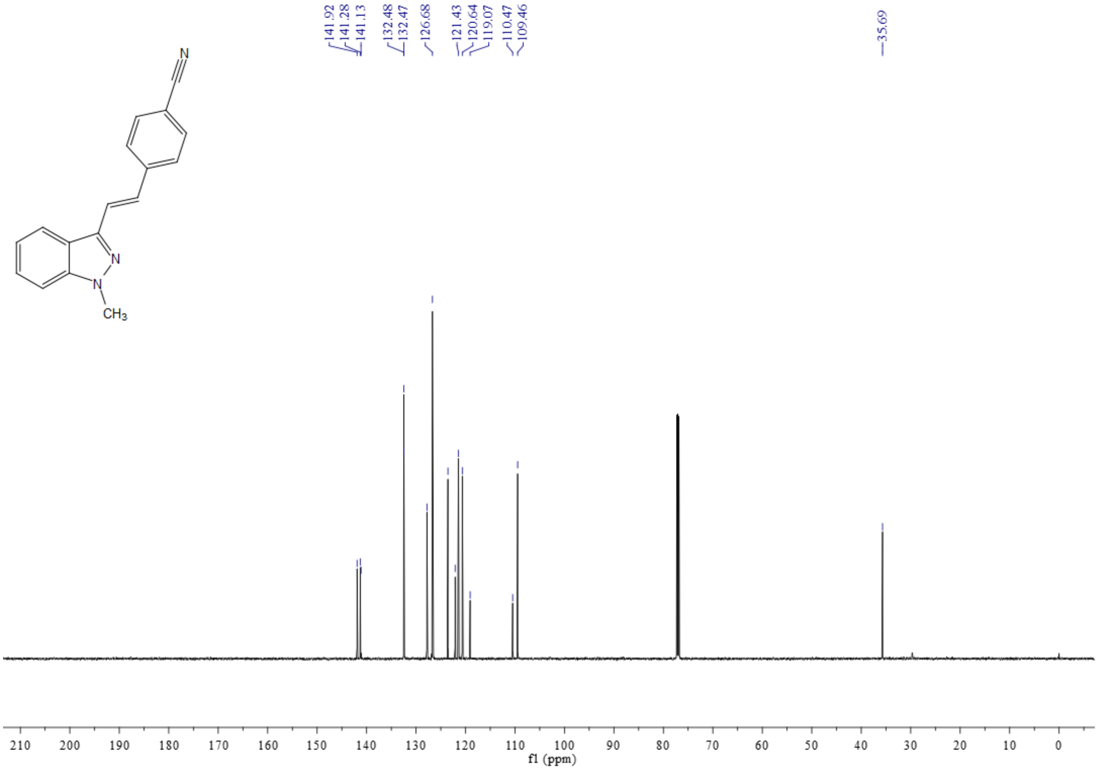

(*E*)-3-(4-Chlorostyryl)-1-methyl-1*H*-indazole (3ak).

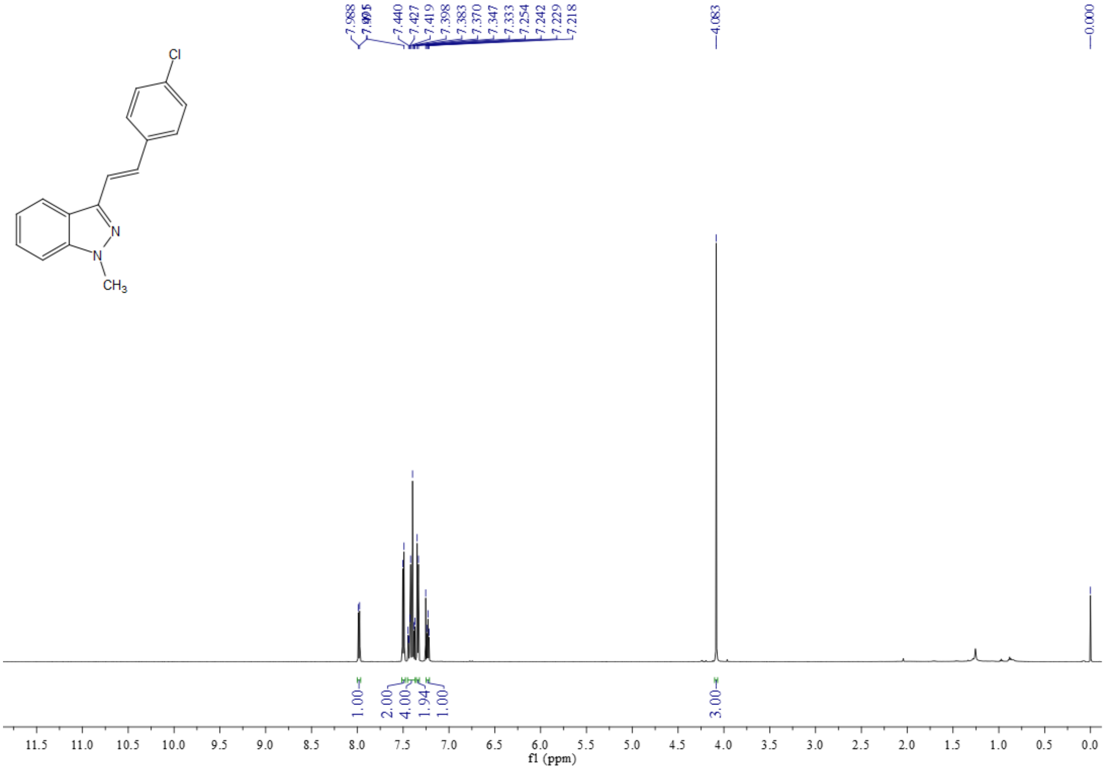

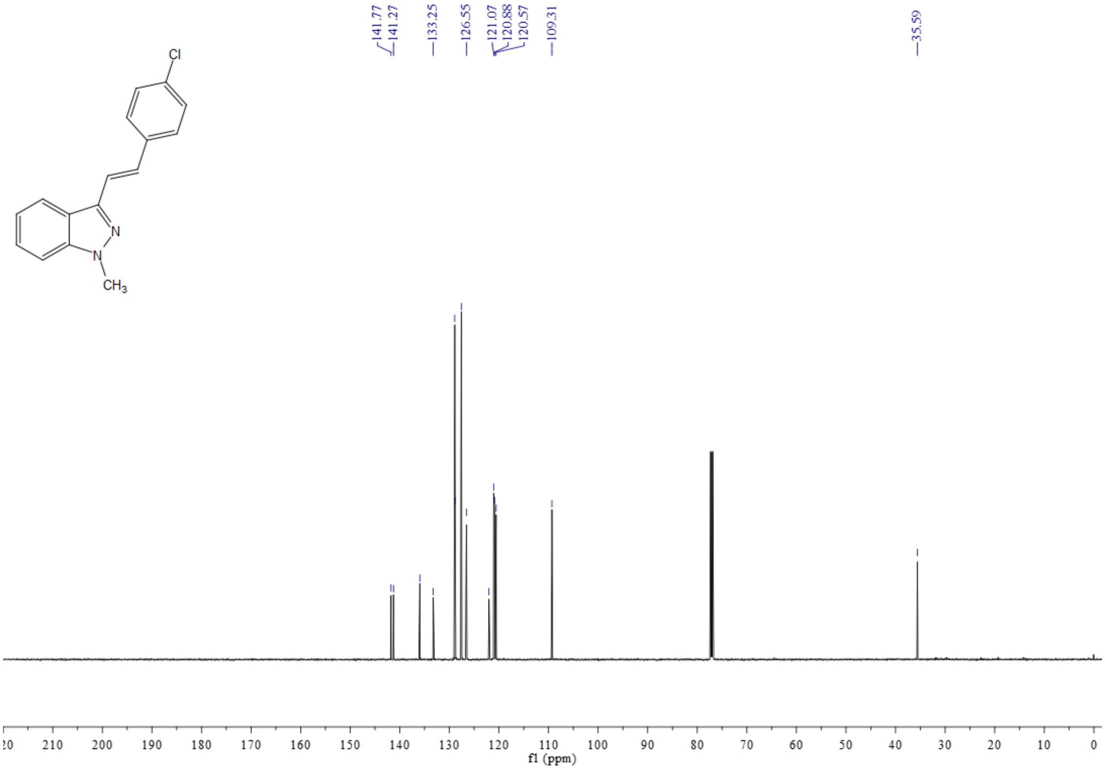

(*E*)-3-(3-Chlorostyryl)-1-methyl-1*H*-indazole (3al).

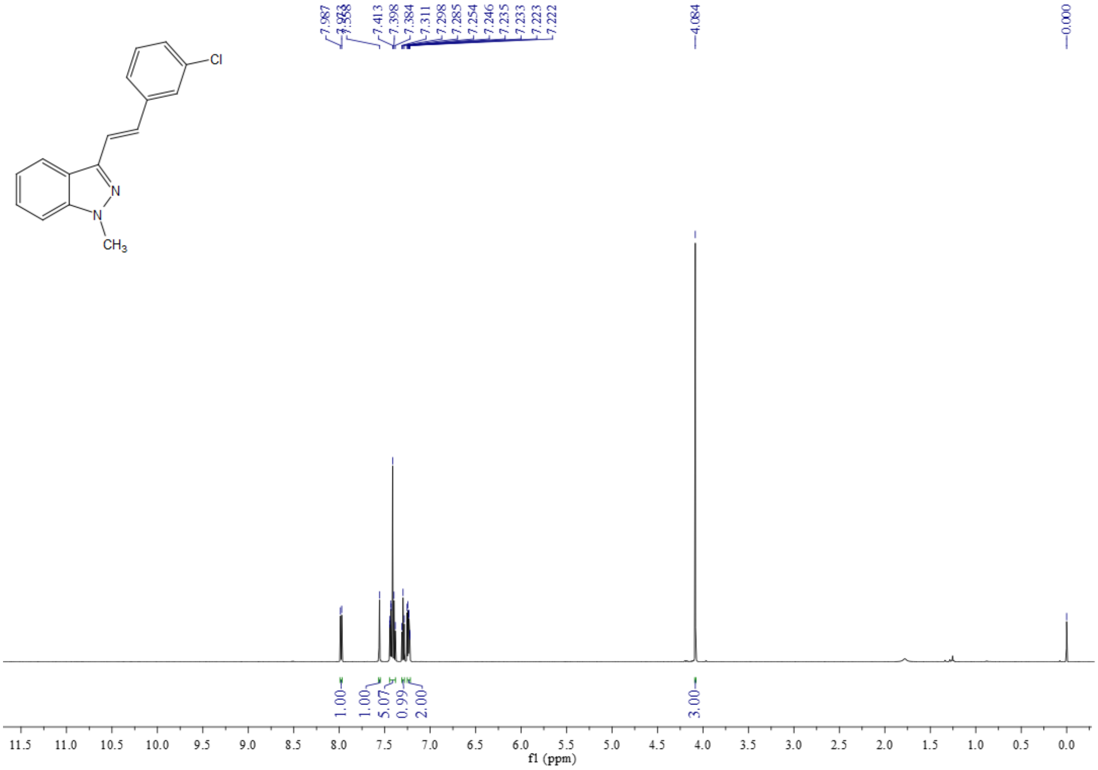

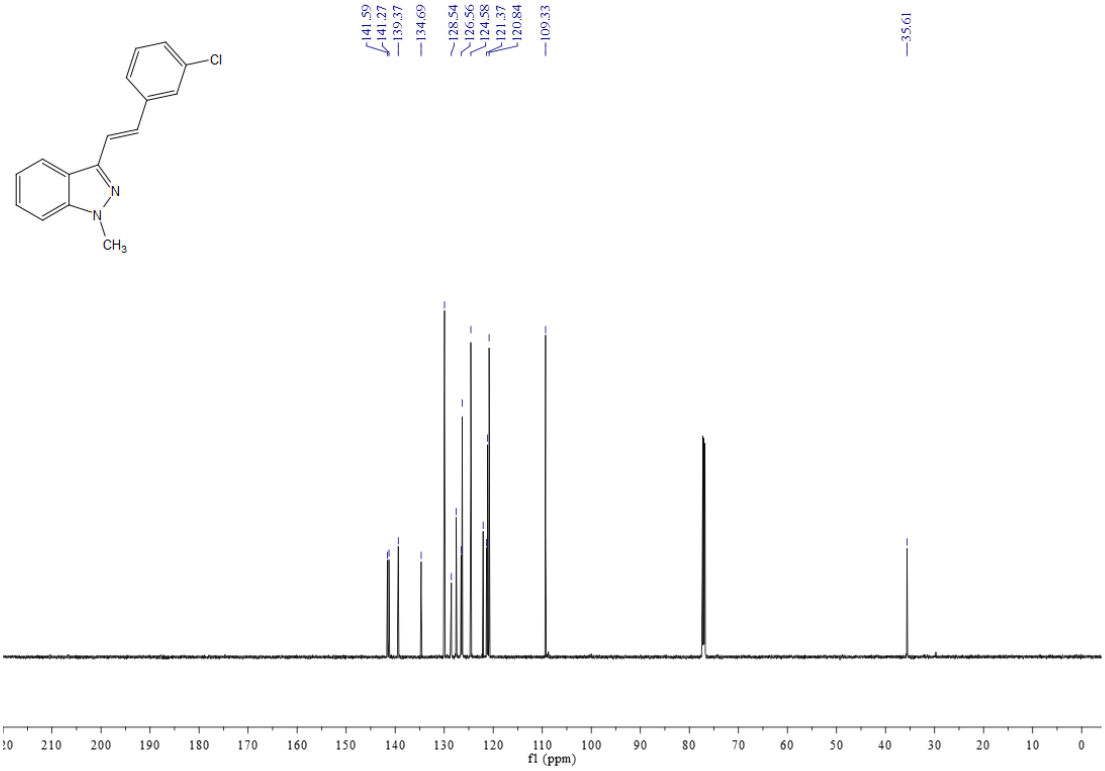

(*E*)-3-(2-Chlorostyryl)-1-methyl-1*H*-indazole (3am).

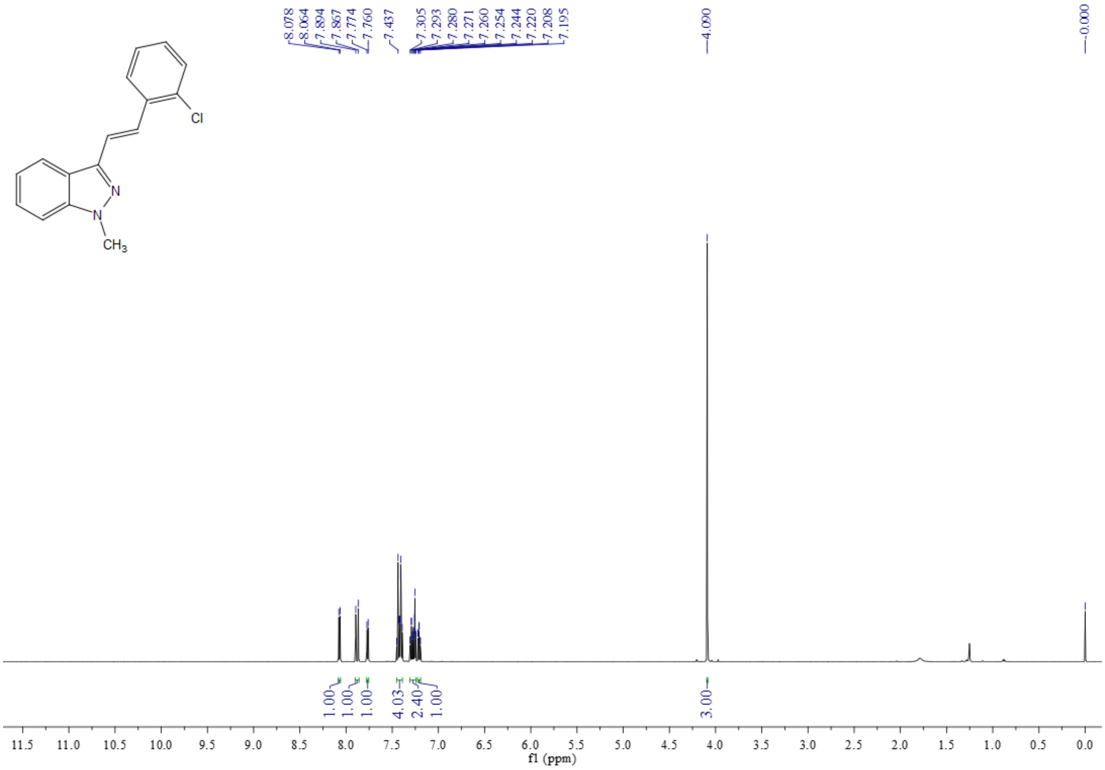

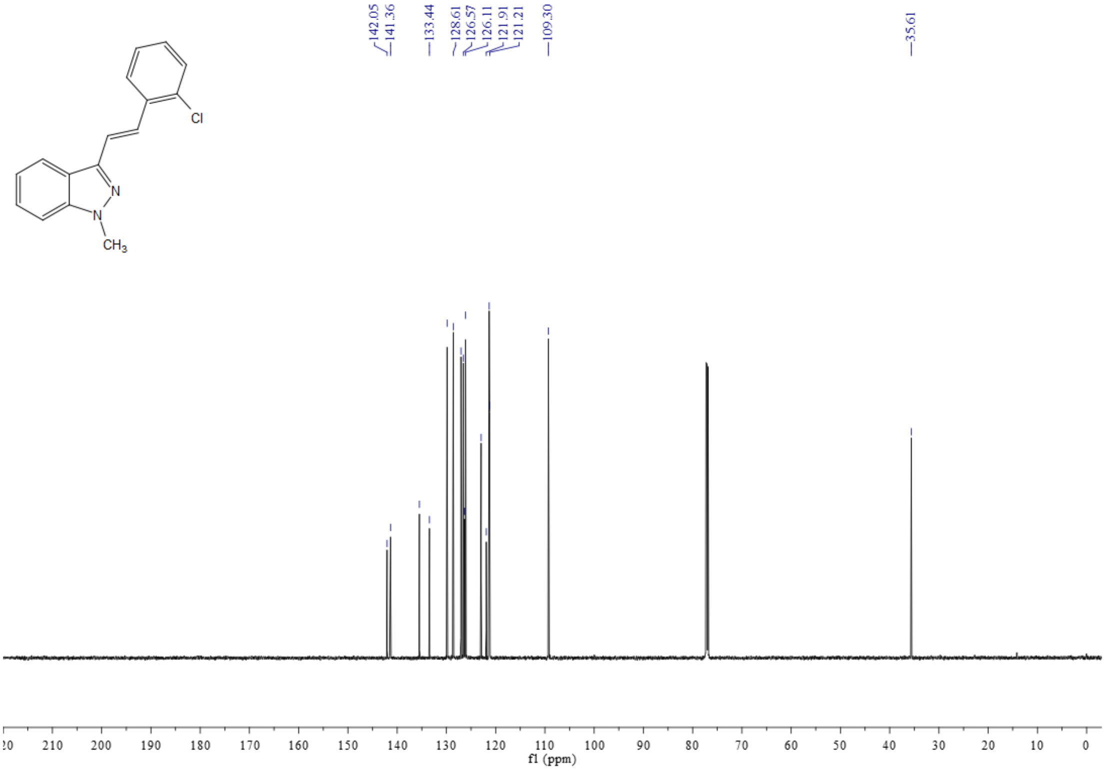

(*E*)-1-Methyl-3-(2-(pyridin-2-yl)vinyl)-1*H*-indazole (3an).

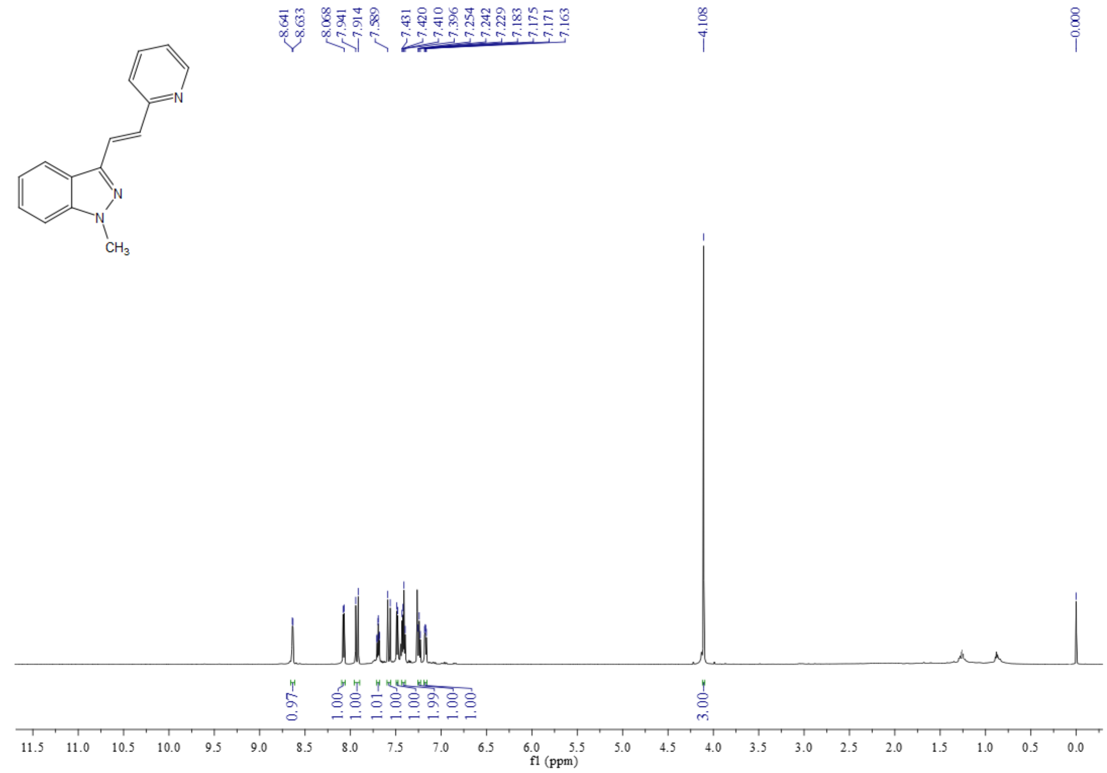

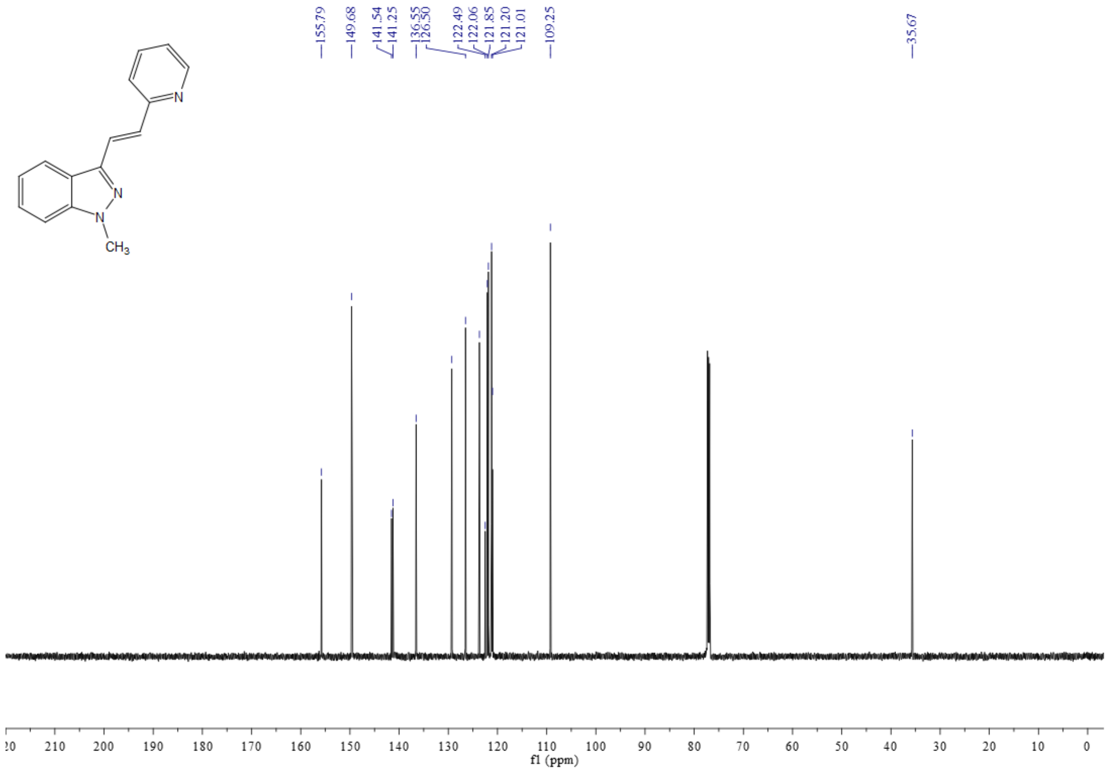

(*E*)-1-Methyl-3-(3-phenylprop-1-en-1-yl)-1*H*-indazole (3ao).

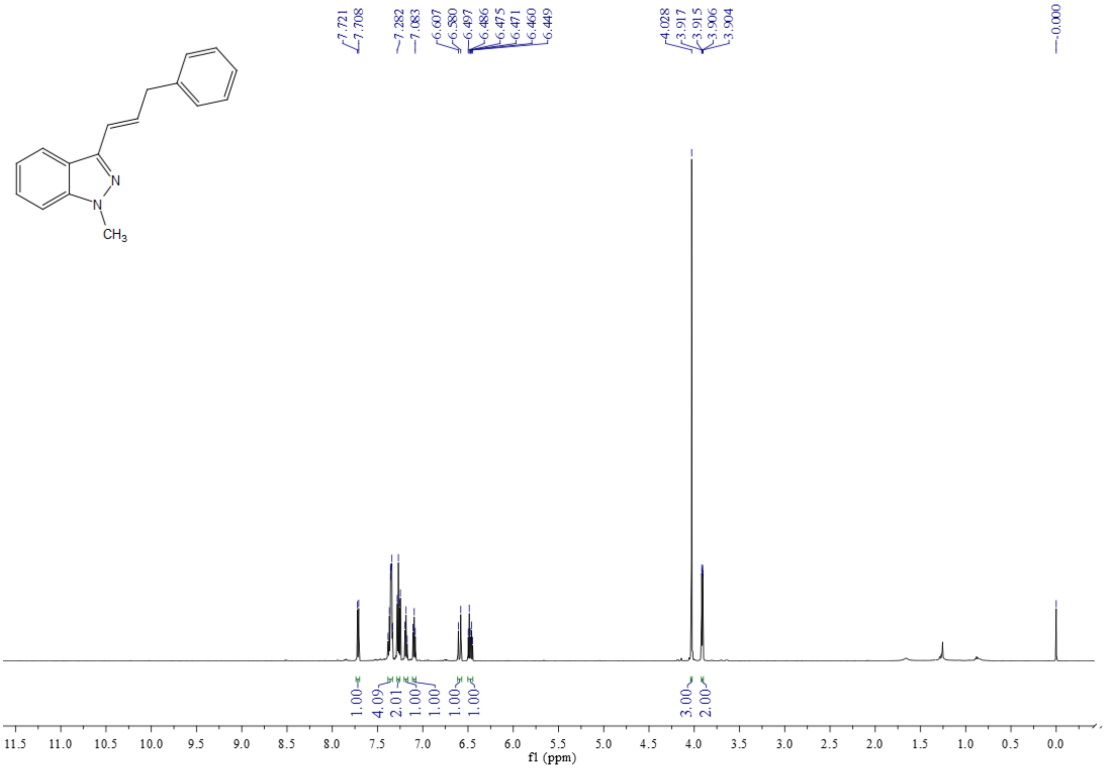

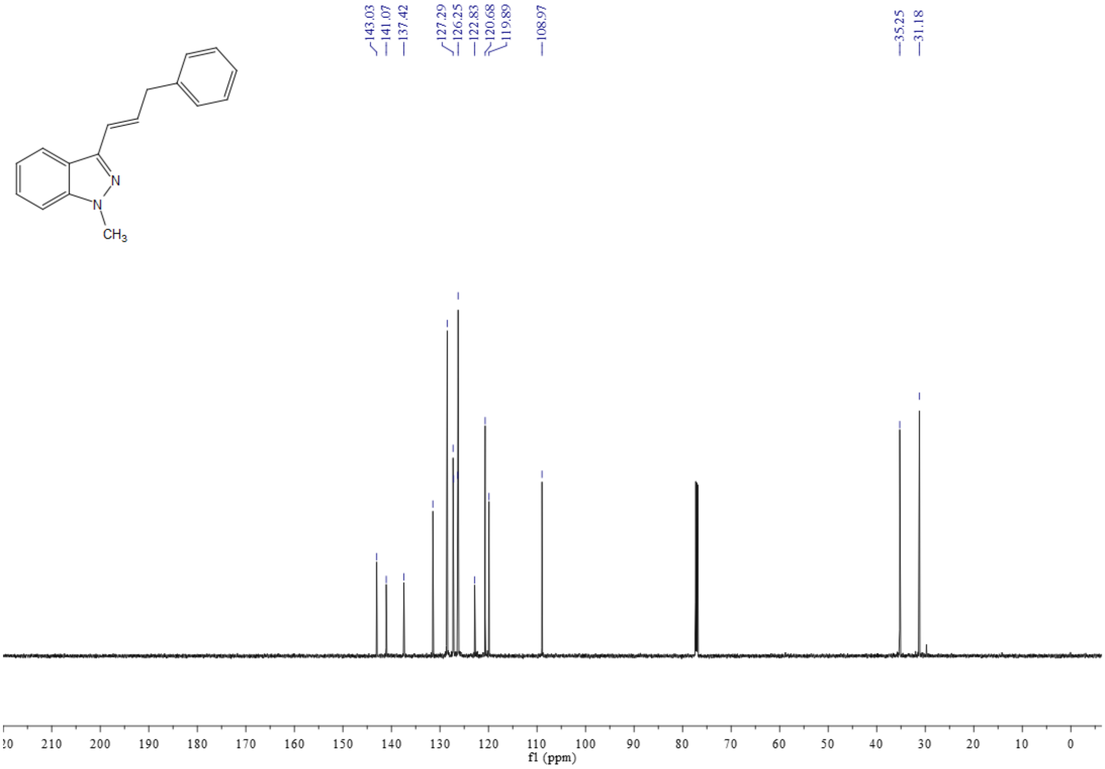

(*E*)-3-(2-(Pyridin-2-yl)vinyl)-1-(tetrahydro-2*H*-pyran-2-yl)-1*H*-indazole (3ln).

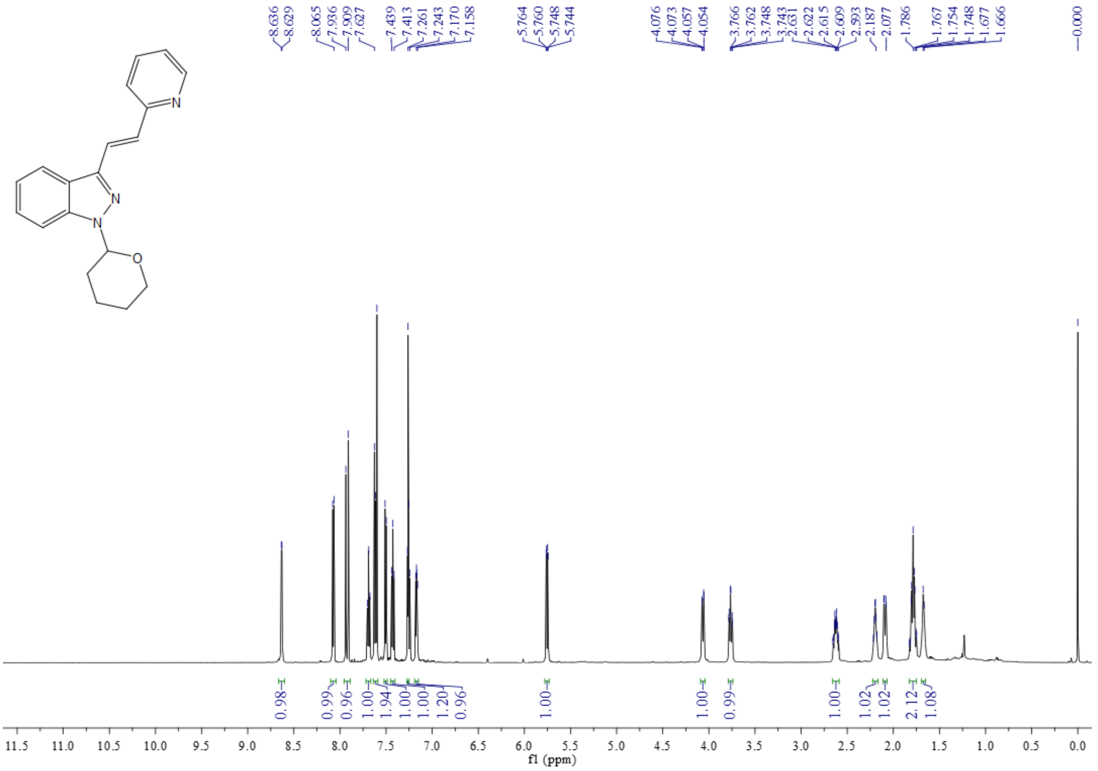

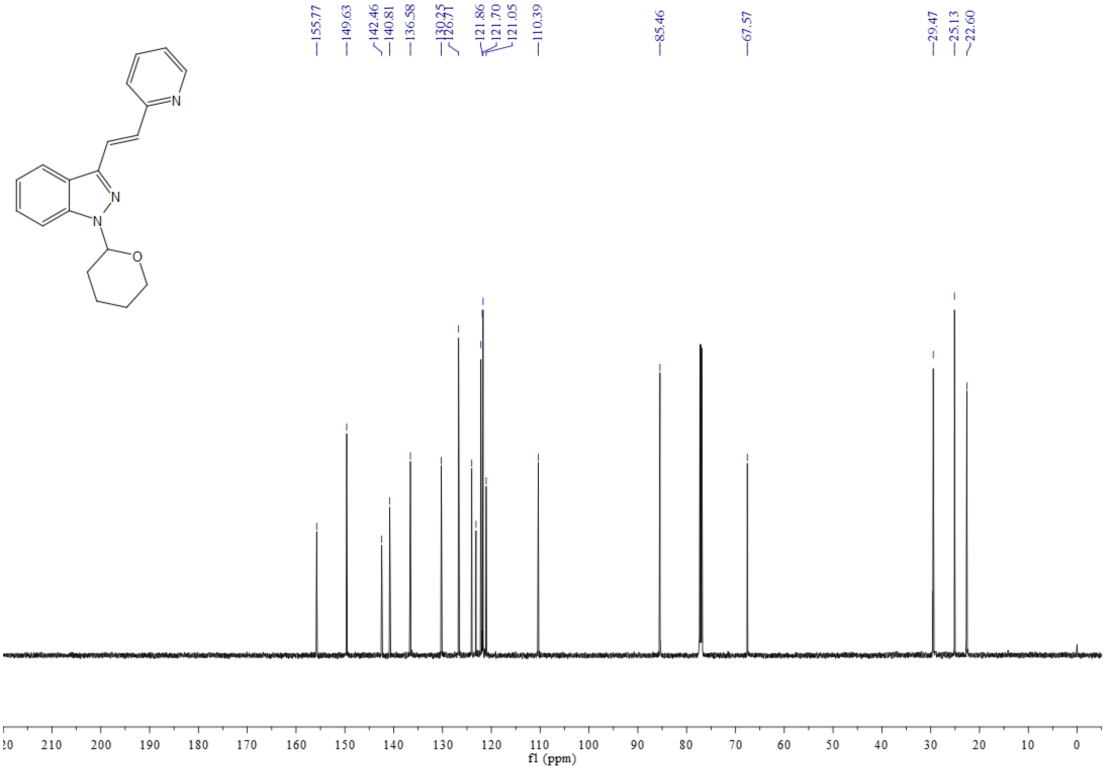

(*E*)-4-(2-(1-Methyl-1*H*-indazol-3-yl)vinyl)phenol (3nf).

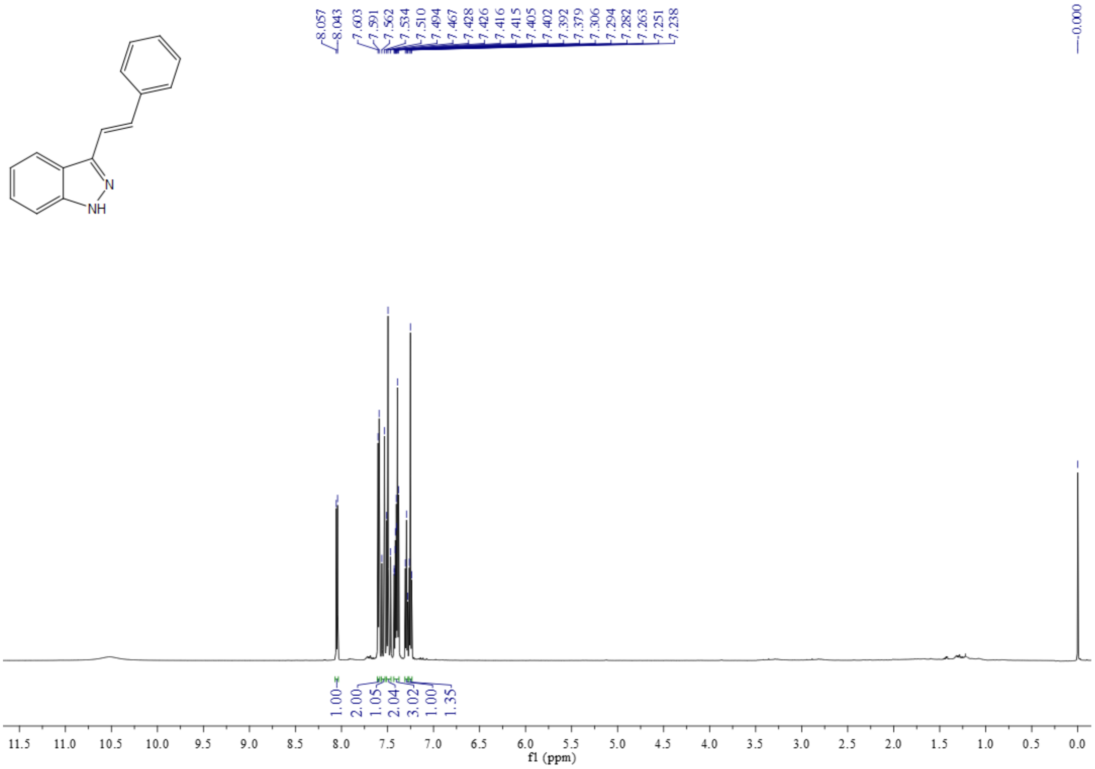

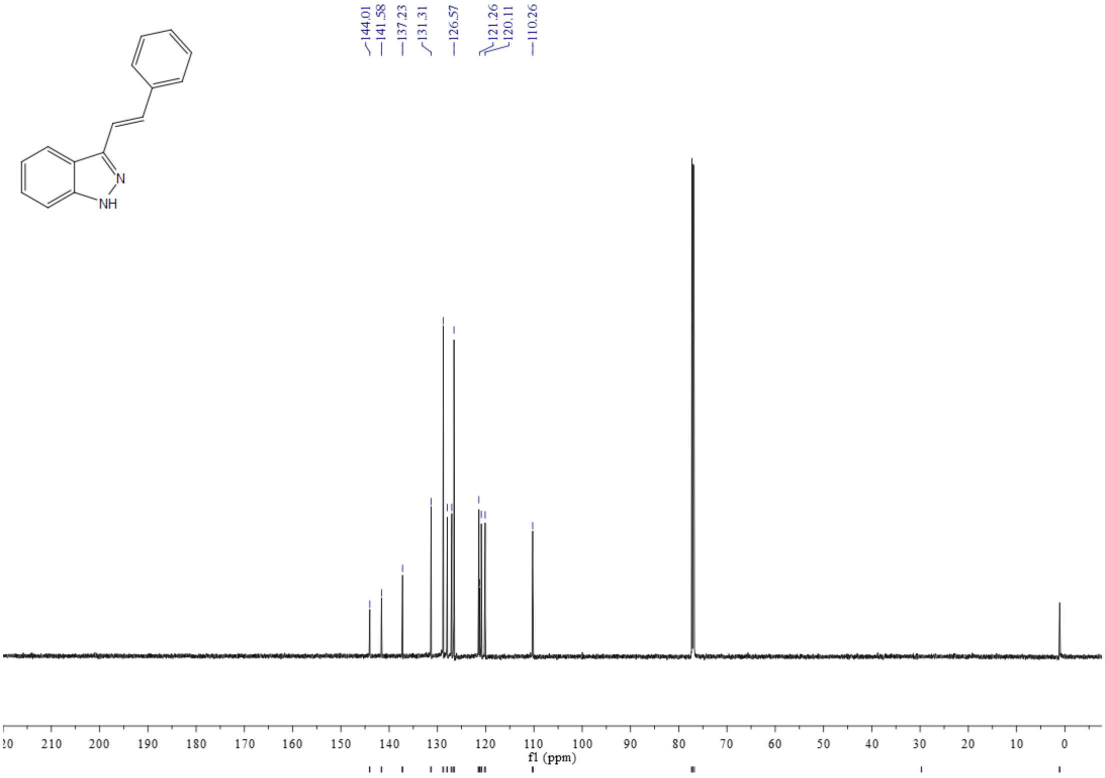

(*E*)-6-Nitro-3-(2-(pyridin-2-yl)vinyl)-1-(tetrahydro-2*H*-pyran-2-yl)-1*H*-indazole (3pn).

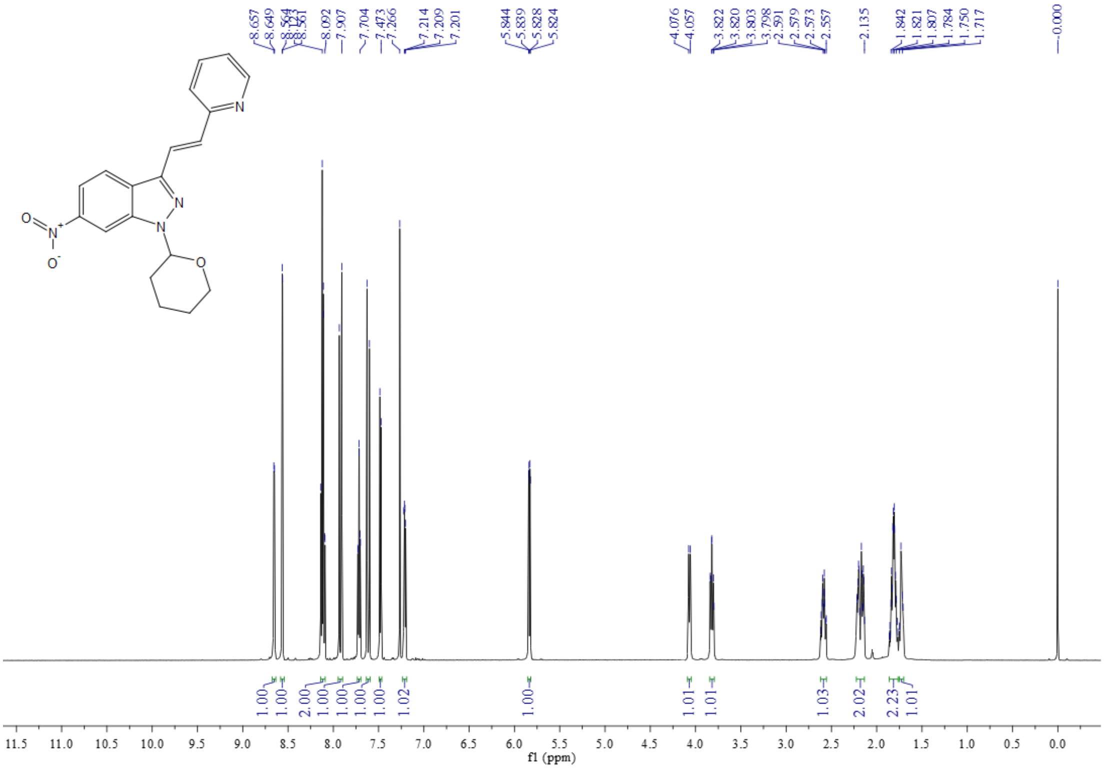

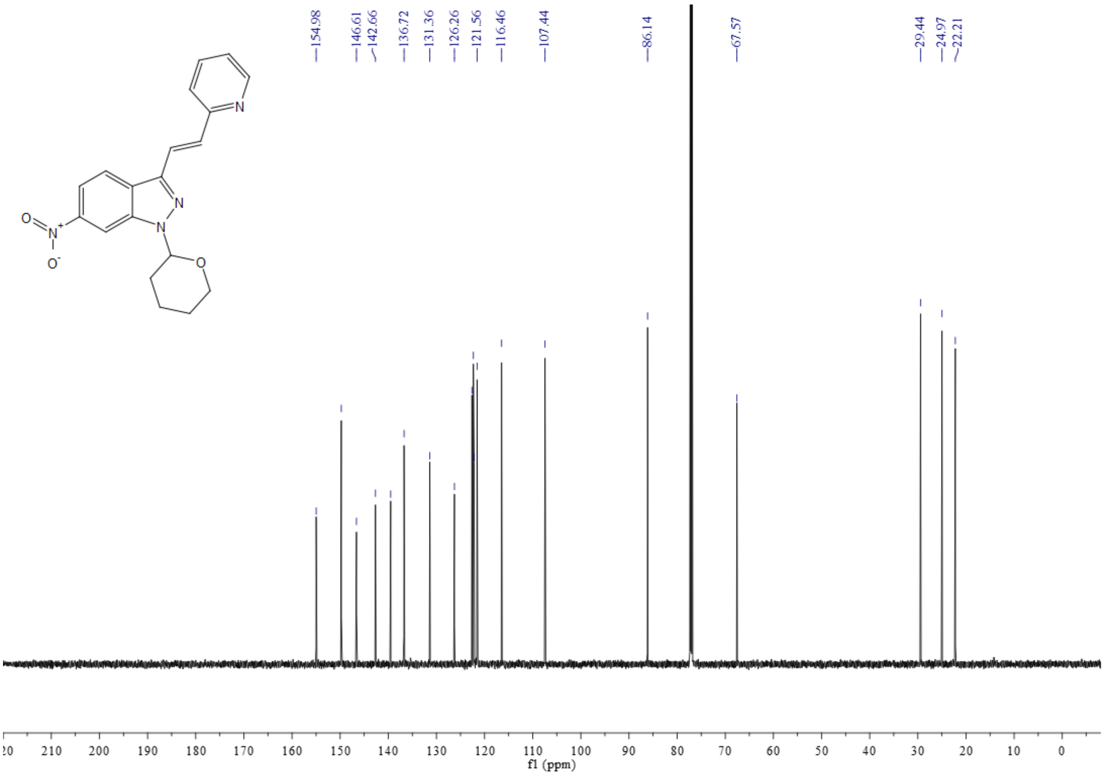

(*E*)-6-Bromo-3-(2-(pyridin-2-yl)vinyl)-1-(tetrahydro-2*H*-pyran-2-yl)-1*H*-indazole (3qn).

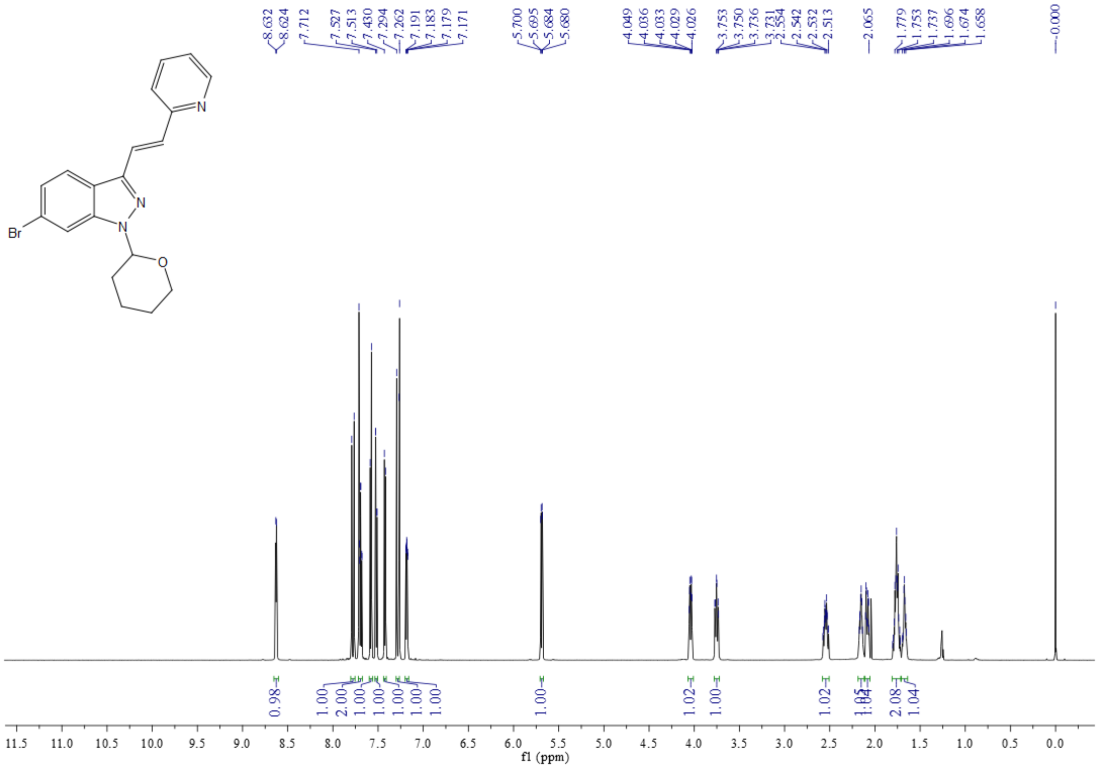

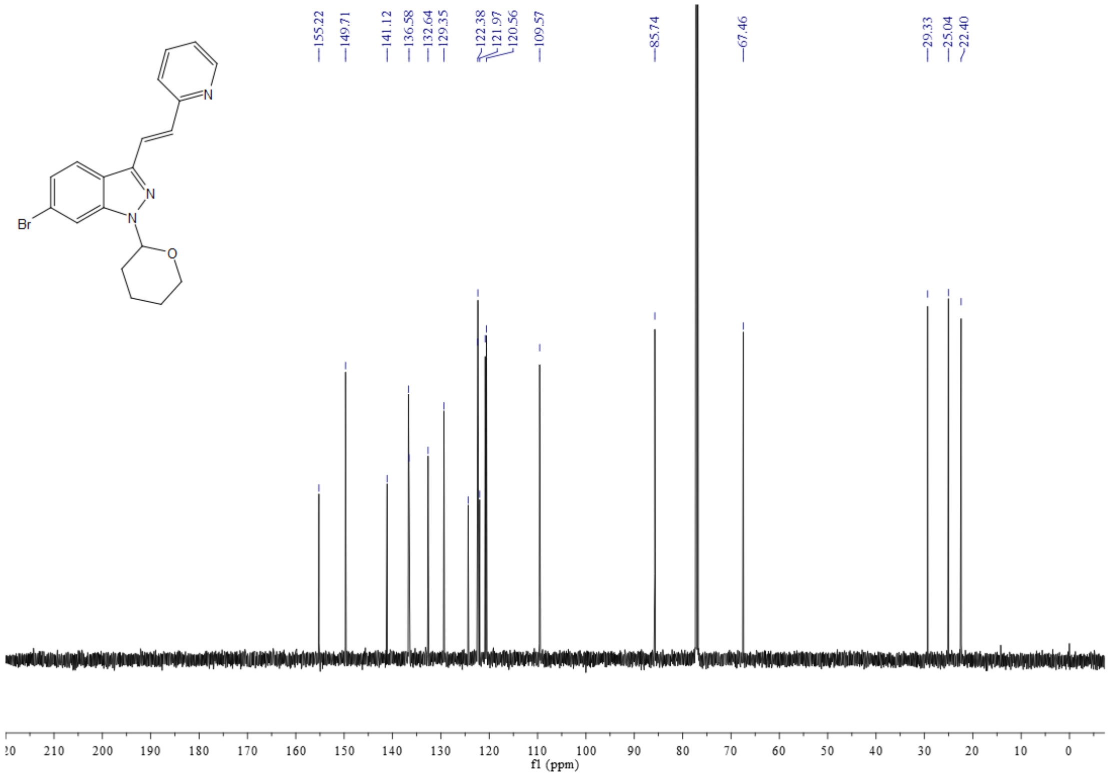

3,6-Dibromo-1-(tetrahydro-2*H*-pyran-2-yl)-1*H*-indazole (3q).

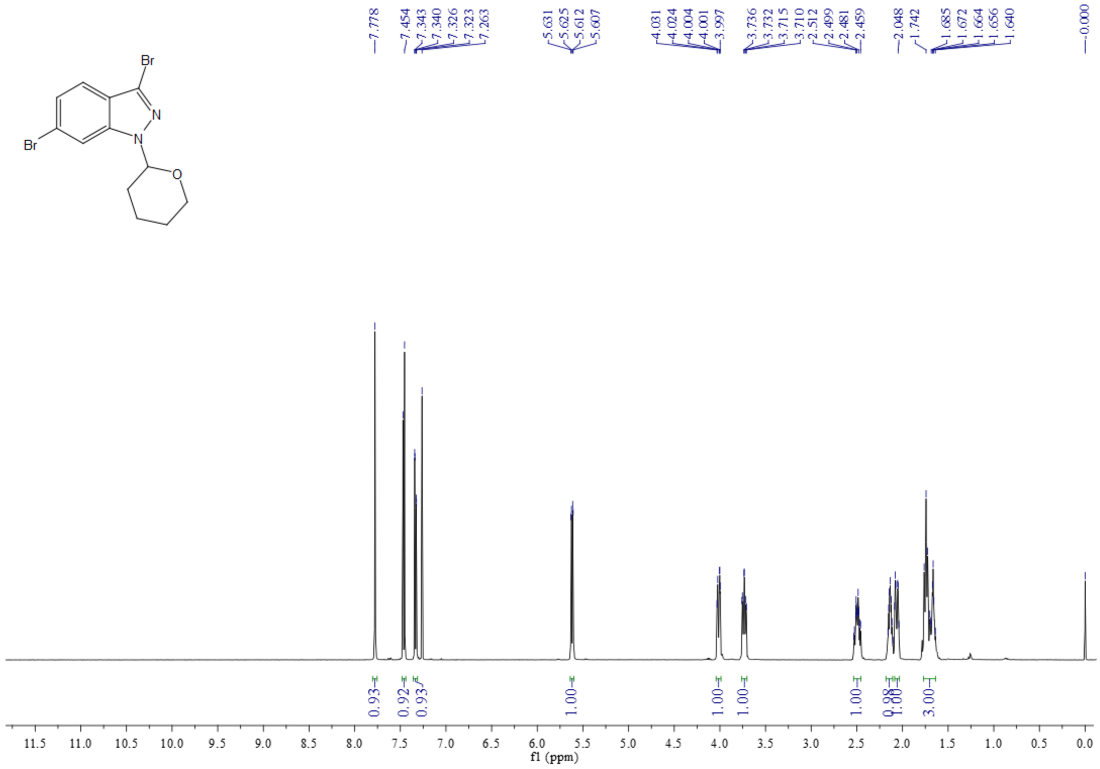

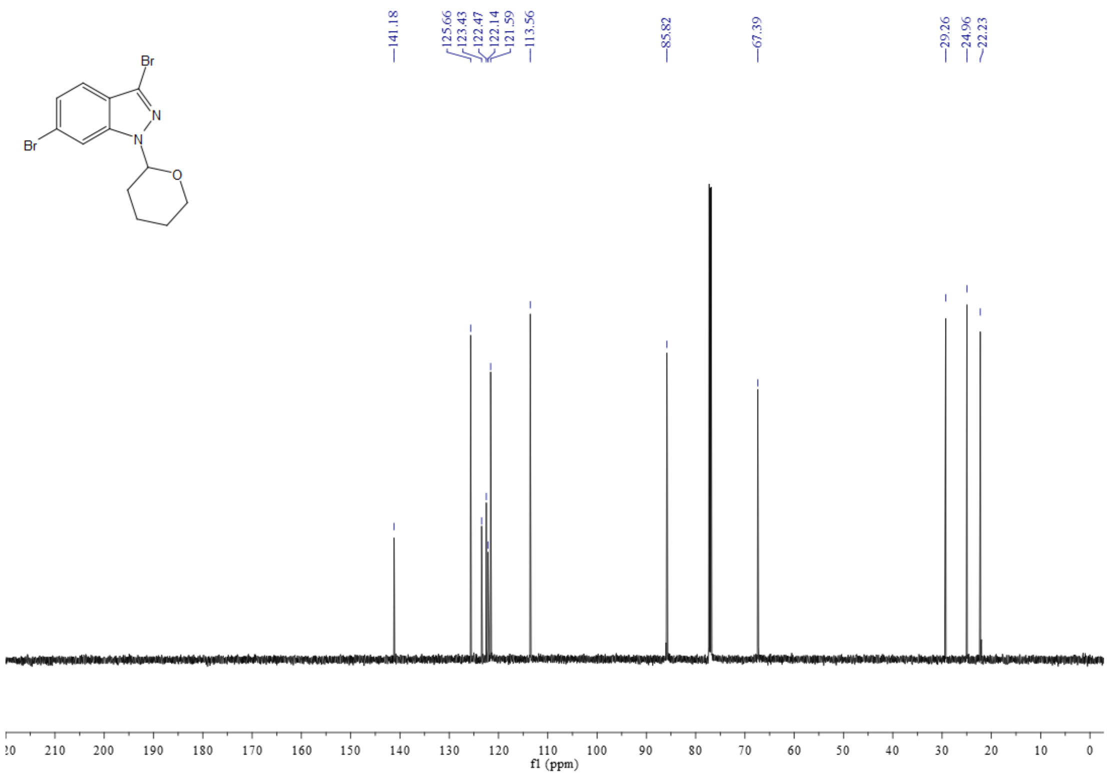

1-Methyl-1*H*-indazole (4a).

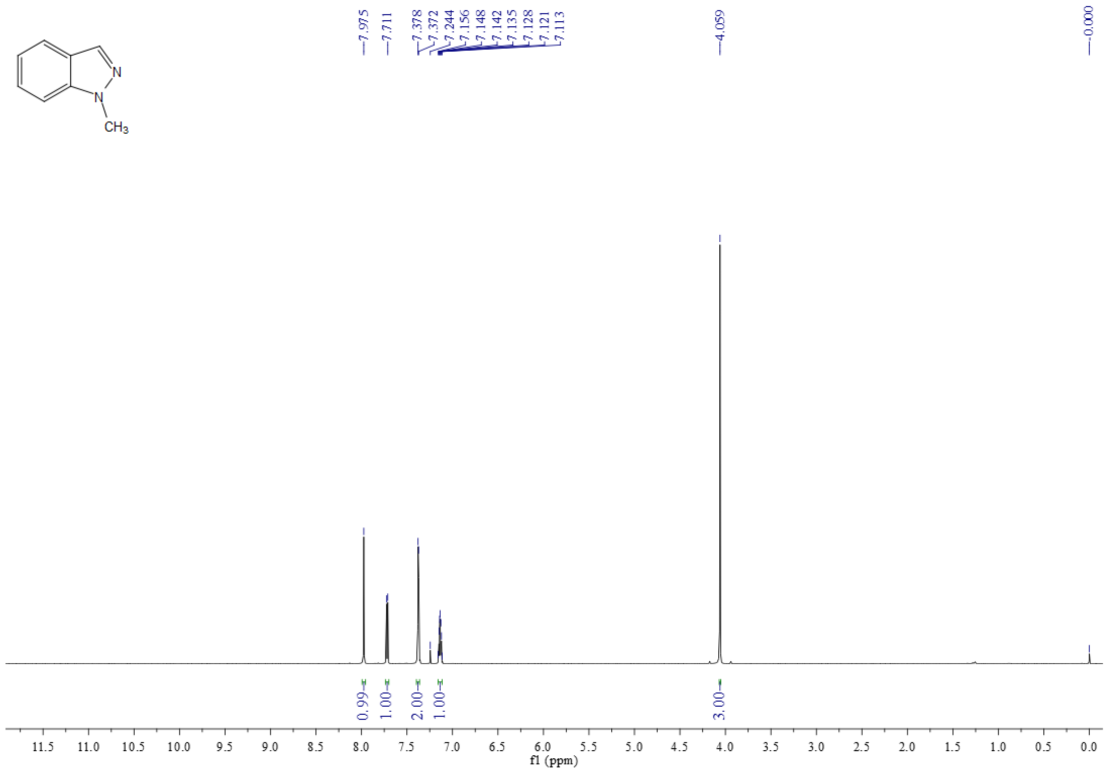

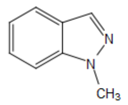

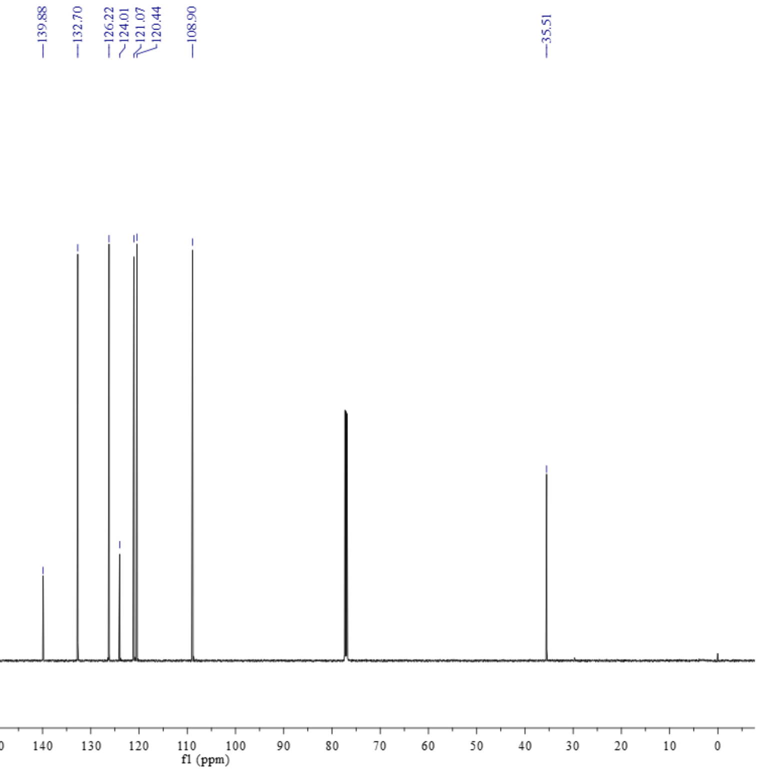
139.88
132.70
126.22
124.01
121.07
120.44
108.90
35.51
210
200
190
180
170
160
150
140
130
120
110
100
90
80
70
60
50
40
30
20
10
0
f1 (ppm)

(*E*)-*N*-Methyl-2-((3-(2-(pyridin-2-yl)vinyl)-1-(tetrahydro-2*H*-pyran-2-yl)-1*H*-indazol-6-yl)thio)benzamide (7).

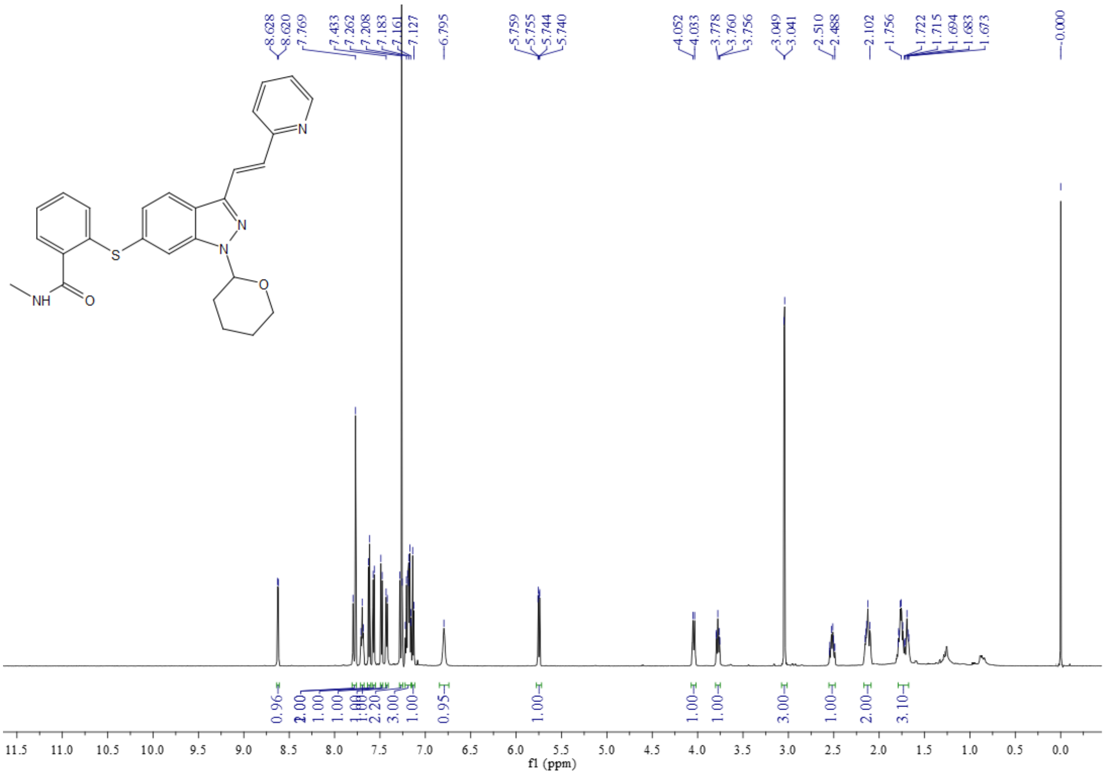

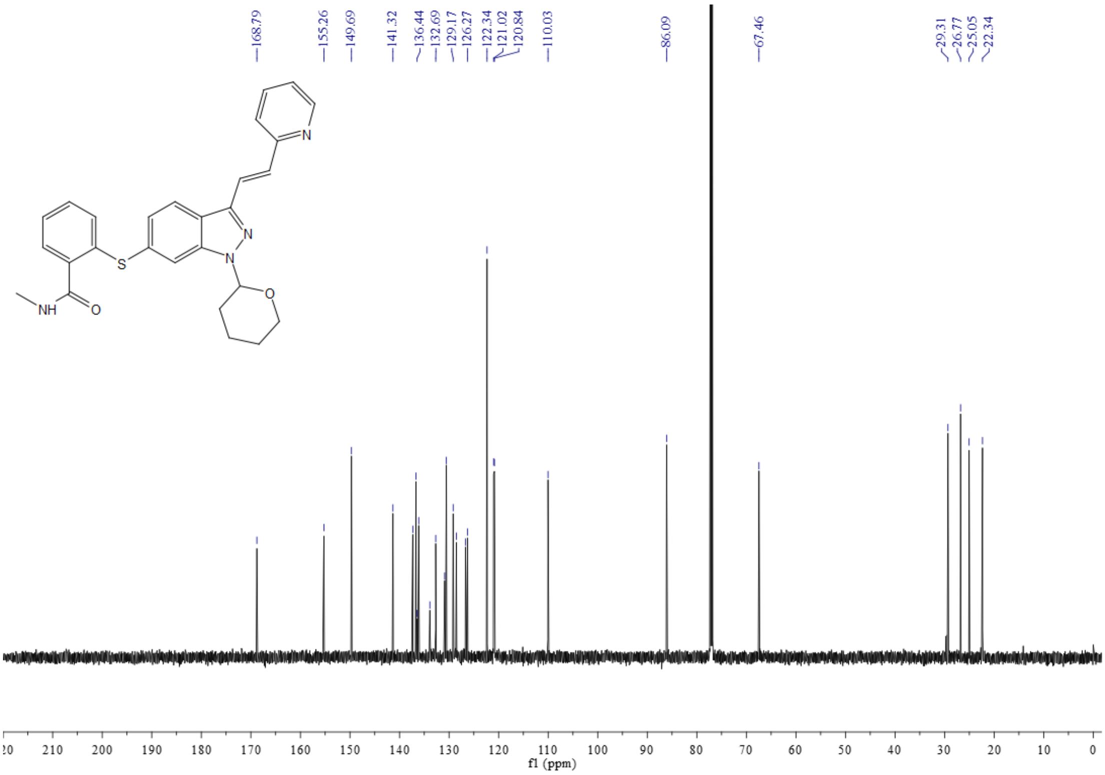

(*E*)-*N*-Methyl-2-((3-(2-(pyridin-2-yl)vinyl)-1*H*-indazol-6-yl)thio)benzamide (axitinib).

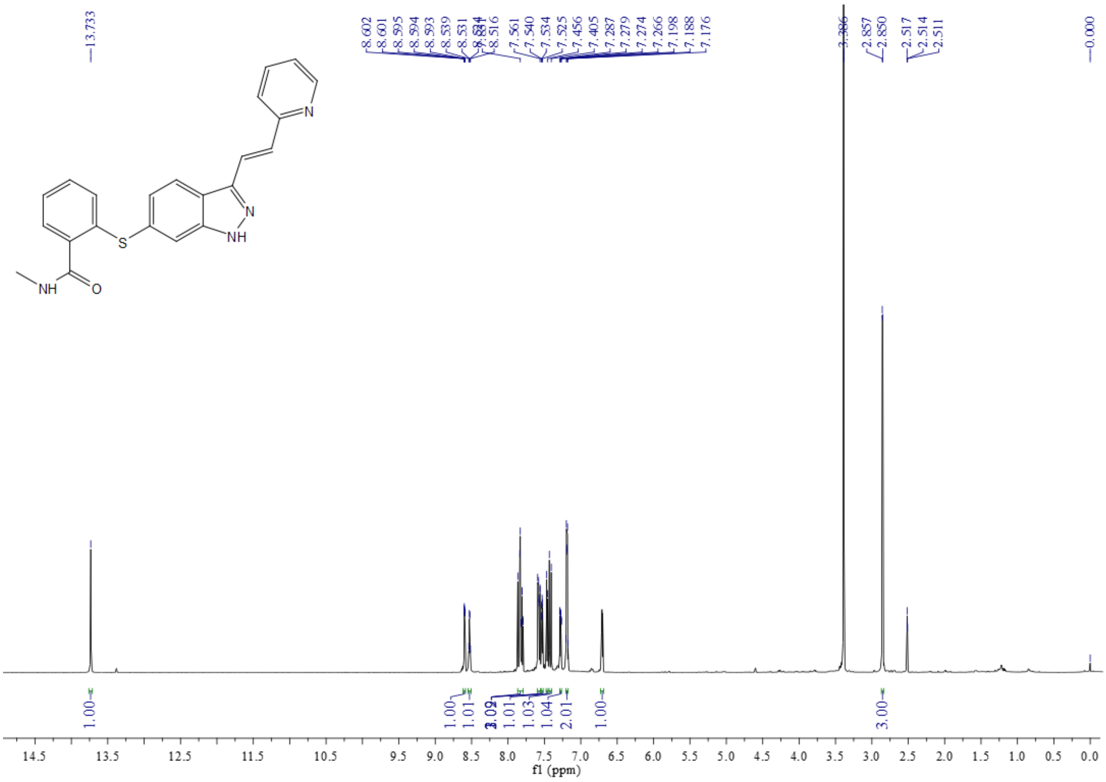

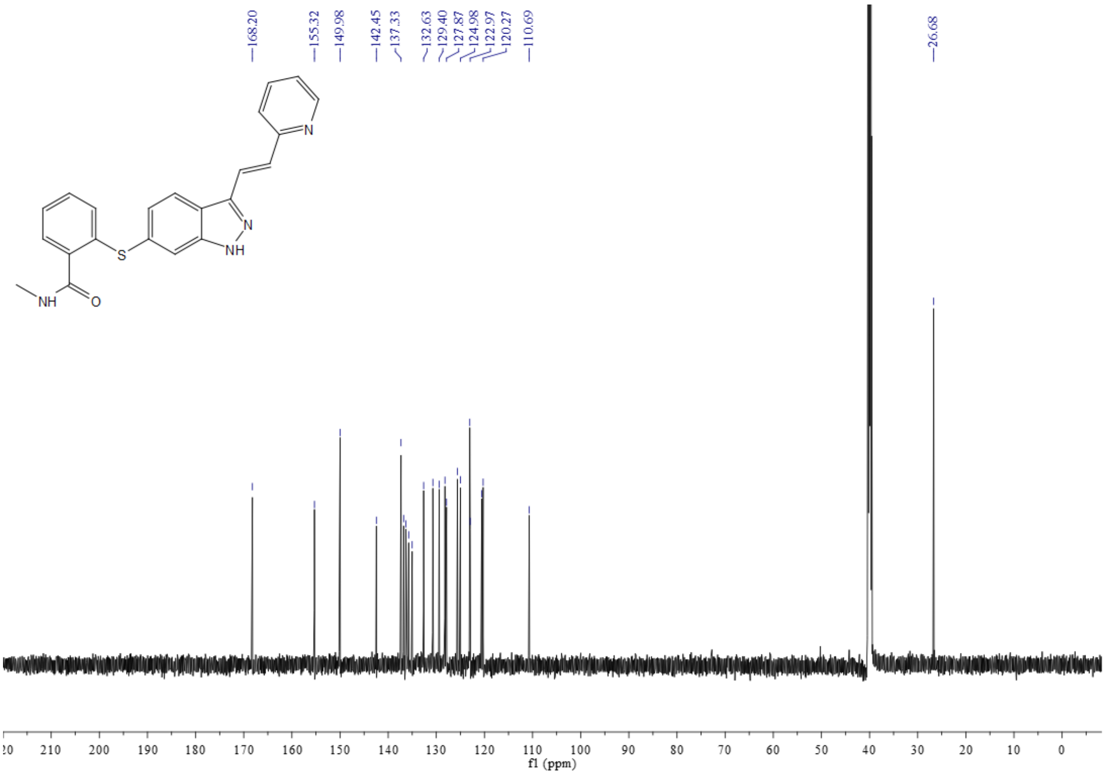

5. References

1. The results of scaling-up experiment.

2. Han, S. J.; Kim, H. T.; Joo, J. M. *J. Org. Chem.* **2016**, *81*, 689–698.

3. Markina, N. A.; A. Dubrovskiy, V.; Larock, R. C. *Org. Biomol. Chem.* **2012**, *10*, 2409–2412.

4. Naas, M.; Kazzouli, S. E.; Essassi, E. M.; Bousmina, M.; Guillaumet, G. *Org. Lett.* **2015**, *17*, 4320–4323.

5. Servi, S.; Akgun, Z. R. *Synth. Commun.* **2002**, *32*, 3399–3405.

6. Ben-Yahia, A.; Naas, M.; Kazzouli, S. E.; Essassi, E. M.; Guillaumet, G. *Eur. J. Org. Chem.* **2012**, *36*, 7075–7081.

7. Zhai, L. H.; Guo, L. H.; Luo, Y. H.; Ling, Y.; Sun, B. W. *Org. Process Res. Dev.* **2015**, *19*, 849–857.

8. Chekal, B. P.; Guinness, S. M.; Lillie, B. M.; McLaughlin, R. W.; Palmer, C. W.; Post, R. J.; Sieser, J. E.; Singer, R. A.; Sluggett, G. W.; Vaidyanathan, R.; Withbroe, G. J. *Org. Process Res. Dev.* **2014**, *18*, 266–274.
